# Unlocking Diabetic Acetone Vapor Detection by A Portable Metal‐Organic Framework‐Based Turn‐On Optical Sensor Device

**DOI:** 10.1002/advs.202305070

**Published:** 2023-11-30

**Authors:** Samraj Mollick, Sujeet Rai, Louis Frentzel‐Beyme, Vishal Kachwal, Lorenzo Donà, Dagmar Schürmann, Bartolomeo Civalleri, Sebastian Henke, Jin‐Chong Tan

**Affiliations:** ^1^ Multifunctional Materials & Composites (MMC) Laboratory Department of Engineering Science University of Oxford Parks Road Oxford OX1 UK; ^2^ Anorganische Chemie Fakultät für Chemie & Chemische Biologie Tec‐hnische Universität Dortmund Otto‐Hahn Straße 6 44227 Dortmund Germany; ^3^ Department of Chemistry University of Turin Via Pietro. Giuria 5 Torino 10125 Italy

**Keywords:** metal–organic frameworks (MOFs), acetone sensing, optical sensors, nanospectroscopy, photoluminescence

## Abstract

Despite exhaled human breath having enabled noninvasive diabetes diagnosis, selective acetone vapor detection by fluorescence approach in the diabetic range (1.8–3.5 ppm) remains a long‐standing challenge. A set of water‐resistant luminescent metal‐organic framework (MOF)‐based composites have been reported for detecting acetone vapor in the diabetic range with a limit of detection of 200 ppb. The luminescent materials possess the ability to selectively detect acetone vapor from a mixture comprising nitrogen, oxygen, carbon dioxide, water vapor, and alcohol vapor, which are prevalent in exhaled breath. It is noteworthy that this is the first luminescent MOF material capable of selectively detecting acetone vapor in the diabetic range via a turn‐on mechanism. The material can be reused within a matter of minutes under ambient conditions. Industrially pertinent electrospun luminescent fibers are likewise fabricated alongside various luminescent films for selective detection of ultratrace quantities of acetone vapor present in the air. Ab initio theoretical calculations combined with in situ synchrotron‐based dosing studies uncovered the material's remarkable hypersensitivity toward acetone vapor. Finally, a freshly designed prototype fluorescence‐based portable optical sensor is utilized as a proof‐of‐concept for the rapid detection of acetone vapor within the diabetic range.

## Graphical abstract

Luminescent composites exhibiting turn‐on acetone detection are demonstrated, with trace detection sensitivity of better than 1 parts‐per‐million (<1 ppm). Furthermore, the sensor material is moisture‐resistant and unaffected by the presence of other volatile organic compounds (VOCs), rendering it attractive for multicyclic applications.

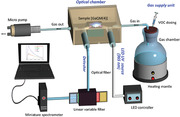

## 1 Introduction

Selective and precise identification of various volatile organic compounds (VOCs) in the vapor phase is of utmost importance in many sectors, including environmental monitoring, food quality control, and industrial safety.^[^ ^1^, ^2^ ^]^ Of late, selective detection and quantification of such VOCs spurred significant scientific attention, as it offers noninvasive means of diagnosing various health conditions in their early stages.^[^ ^3^, ^4^ ^]^ Breath acetone has long been recognized as a biomarker for diabetes, with diabetic patients typically having an elevated level of acetone concentration in their exhaled breath, measures in parts‐per‐million (ppm) typically between 1.8 and 3.5 ppm (vs 0.2–1.8 ppm for healthy individuals).^[^ ^5^, ^6^ ^]^ Portable sensors are considered a potential alternative solution for rapid diagnosis of diabetes in the early stages by breath monitoring compared to currently employed cumbersome gas chromatography‐mass spectrometry techniques.^[^ ^7^, ^8^, ^9^, ^10^ ^]^ Several portable gas sensor devices have undergone extensive research to detect acetone vapor at levels of ppm or parts‐per‐billion (ppb).^[^ ^11^, ^12^ ^]^ The majority of these devices utilize metal oxide semiconductors that rely on chemisorption, redox, and catalysis mechanisms. A major drawback of oxide‐based breath sensors is the requirement for operation at an elevated temperature of ca. 200–400 °C. In this context, fluorescent method for detecting acetone vapor has been identified as one of the most promising techniques due to its high level of sensitivity, noninvasiveness, real‐time monitoring, ease of use, and fast response time. Nevertheless, portable sensor devices based on the fluorescent method are yet to be studied for diabetic acetone vapor detection. The major roadblocks are the extremely poor sensitivity in the diabetic range, the absence of selectivity in the vapor phase, and the water instability of the mainstream luminescent materials.

Metal‐organic frameworks (MOFs) are a class of crystalline porous frameworks that are composed of metal ions or clusters and organic ligands.^[^ ^13^, ^14^, ^15^ ^]^ MOFs have garnered immense interest, particularly in the field of sensing, owing to their reversible and selective adsorption process, which is a consequence of their remarkable control over functional groups and pore size.^[^ ^16^, ^17^, ^18^, ^19^ ^]^ While numerous luminescent MOFs have been reported to selectively detect acetone in the liquid phase, only a limited number of MOF‐based materials can detect acetone in the vapor phase.^[^ ^20^, ^21^, ^22^ ^]^ Unfortunately, none of these materials is suitable for diabetes breath acetone detection by fluorescent method because of their inadequate acetone vapor sensitivity in sub‐ppm levels, lack of selectivity, and inferior water stability, which severely restricts their practical use for monitoring acetone in exhaled breath. Moreover, the majority of the current MOF‐based acetone sensing methods in the vapor phase operate on a “turn‐off” mechanism (luminescent intensity declines upon analytical detection), rendering them vulnerable to erroneous readings caused by ambient factors such as temperature and humidity.^[^ ^23^, ^24^, ^25^, ^26^ ^]^ Therefore, the pressing need for a luminescent MOF‐based material that exhibits vapor phase “turn‐on” sensing behavior (intensity increases on detection) combined with high sensitivity in ppm levels, superior selectivity along with excellent water‐resistive properties, has remained a long‐standing challenge in the field.

Herein, we have judiciously designed a family of exceptional water and photo‐resistant luminescent MOF composites by integrating luminescent metal hydroxyquinolates into the metal azolate framework (MAF)−5 host. The resultant composite can rapidly detect ultratrace quantities of acetone vapor, even at the diabetic range with a detection limit of 200 ppb, through a turn‐on fluorescent method and can be reusable within minutes (<3 min) under ambient conditions. Moreover, the composite can selectively detect sub‐ppm levels of acetone vapor from a mixture of different VOCs under ambient conditions, particularly when subject to a combination of N_2_, O_2_, CO_2_, water vapor, and alcohol, which are common in human breath. To the best of our knowledge, there is no exemplar of any luminescent material that exhibits water stability and selective sensing of acetone vapor within the diabetic range (1.8–3.5 ppm) by turn‐on fluorescent approach. We show that the MAF‐5 compound plays a pivotal role in attaining a high level of sensitivity and selectivity targeting low concentrations of acetone molecules. To demonstrate the practical applicability of these materials, different prototypes were fabricated, including a variety of luminescent membranes and industrially relevant chemically resistant luminescent fibers. We employed in situ synchrotron‐based dosing experiments in conjugation with density functional theory (DFT) calculations to unfold the unusual acetone selectivity and sensitivity of the composite. Finally, we demonstrated a portable optical sensor device based on the fluorescence method matching the detection efficiency of a standard benchtop spectrofluorometer for the rapid detection of acetone vapor in the diabetic range, leveraging the materials' unprecedented sensitivity. It is worth mentioning that the demonstration of a portable sensor device capable of detecting acetone vapor using a fluorescence approach had not been shown prior to this study.

## 2 Results and Discussion

### 2.1 Synthesis and Characterization

**Figure 1:**
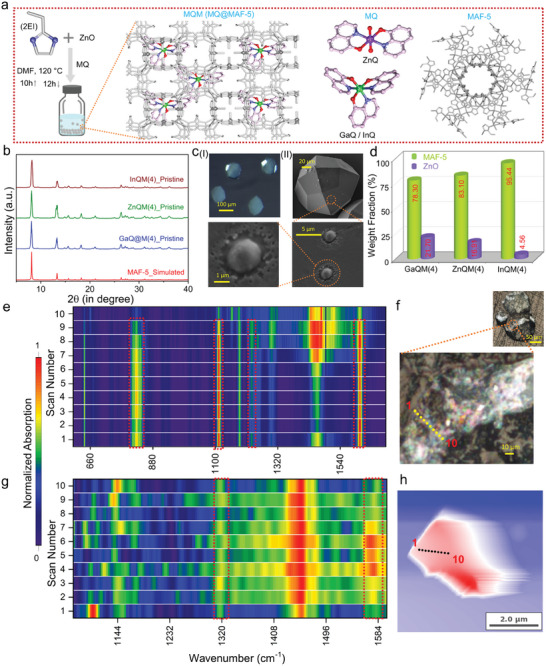
Synthesis and characterizations of composites. a) Schematic illustration of the synthesis of luminescent MQM (MQ@MAF‐5) composites. (Hydrogen atoms are omitted and MAF‐5 is shown as gray color for clarity. Color code: Zn, violet; Ga and In, green; N, blue; C, light pink; O, red). Here MAF‐5 host is denoted as grey color. b) PXRD patterns of simulated MAF‐5, pristine GaQM(4), pristine ZnQM(4) and pristine InQM(4). c) (I) Photograph of GaQM(4) from stereo optical microscope. (II) FESEM micrographs of GaQM(4). d) Bar chart representing the phase fractions of MAF‐5 and ZnO in the MQM(4) materials extracted from the Rietveld fits. e) MicroRaman spectra of a GaQM(4) crystal determined from the highlighted region in (f). Ten spots on the image (f) are related to ten different scans on the crystal. The probe size of microRaman was ≈1.5 µm × 2 µm. g) NanoFTIR absorption spectra of a GaQM(4) crystal taken across the highlighted region in (h). Ten spots on the image (h) are related to ten different scans on the local regions of the single crystal, with a probe size of ≈20 nm. The presence of the shadow in the height image was attributed to the artifacts caused by scanning a “large” micron‐sized crystal.

All of the luminescent composites described in this study were synthesized utilizing solvothermal reaction conditions, as detailed in the Supporting Information (**Figure** **1**a). Three metal hydroxyquinolate guests, namely ZnQ, GaQ, and InQ, were identified and highly water‐stable MAF‐5 was utilized as a host matrix to prepare the various luminescent composites designated as MQM(4) (MQ@MAF‐5), where MQ stands for metal hydroxyquinolate (guest), M represents MAF‐5 (host) and 4 represents the 4 mL guests (MQ) were used during synthesis. ^[^ ^27^ ^]^ Here, MQ was utilized as a luminescent guest while MAF‐5 functions as a highly water‐stable host framework, yielding the water‐resistance luminescent composites.^[^ ^28^ ^]^ A comprehensive structural analysis of the composites was conducted utilizing a range of techniques, including powder X‐ray diffraction (PXRD) with Rietveld refinement, scanning electron microscopy (SEM), Fourier transform infrared spectroscopy (FT‐IR), Raman microspectroscopy (microRaman), and near‐field infrared nanospectroscopy (nanoFTIR). The formation of the high degree of purely crystalline and bulk phase of the composite was validated by PXRD studies (Figure 1b). The unaltered PXRD patterns demonstrate that the composites preserve their crystallinity and structural integrity even after long‐term water immersion, revealing their remarkable water‐resistant features (Figure S1, Supporting Information). As shown in Figure S2 (Supporting Information), the nitrogen sorption isotherms at 77 K revealed a relatively lower adsorption uptake (thus reduced surface area and pore volume) in the composite materials when compared to the pristine MAF‐5. This finding supports the successful integration of the luminous guests into the MOF matrix. Notably, ^1^H nuclear magnetic resonance (NMR) spectroscopy of acid‐digested MQM samples indicates an extremely low loading (<<1 mol%, below the detection limit of ^1^H NMR spectroscopy) of the metal hydroxyquinolate guests in the composites (Figure S3, Supporting Information).

**Figure 2:**
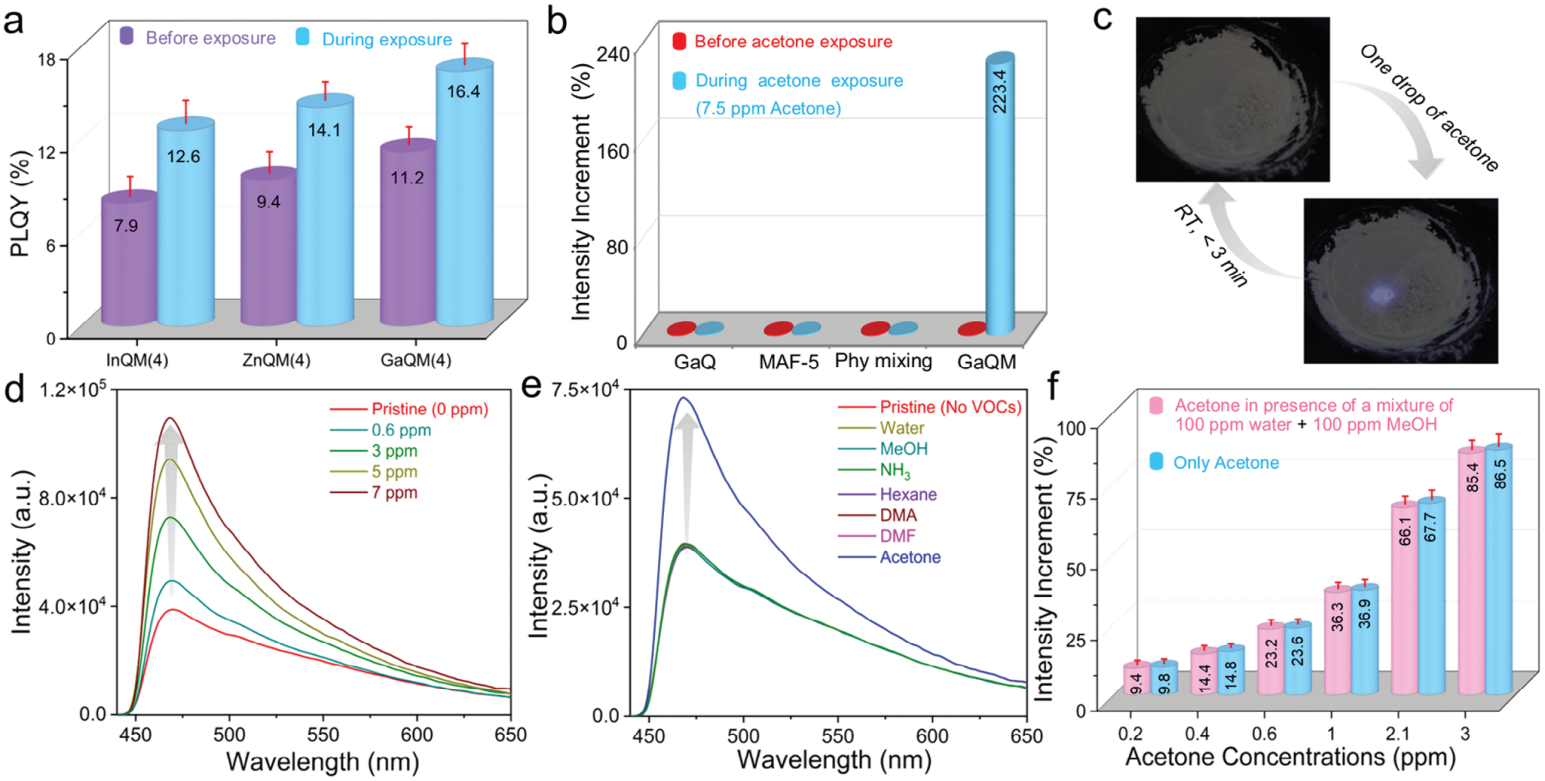
Luminescent sensing of powder sample. a) Photoluminescence quantum yield (PLQY) of various MQM(4) composites before and during exposure to the acetone vapor. b) Intensity increments in emission property of different materials before and during acetone vapor exposure. 7.5 ppm of acetone vapor was used for testing each sample. c) Photographs of powder phase of optochemically‐active GaQM(4) demonstrating reversible acetone sensing ability. d) Turn‐on emission at 468 nm (excitation at 400 nm) of GaQM(4) in the presence of various acetone vapor concentrations in the diabetic range. e) Turn‐on emission spectra of GaQM(4) for acetone vapor selectivity in the mixture containing other VOCs of concentrations of 3 ppm each. Abbreviation of solvents: MeOH, methanol; DMF, N, N‐dimethylformamide; DMA, N, N‐dimethylacetamide f) Acetone detection sensitivity by GaQM(4) composite within the diabetic range from a mixture comprising 100 ppm of water vapor and 100 ppm methanol vapor.

The morphological features of the composite were investigated through SEM analyses (Figure S4, Supporting Information). The SEM micrographs divulged that the size of the composite's single crystal spanned from 70 to 120 µm, and submicron‐sized (≈2 µm) spherical particles were also observed on the crystal surface of the composite (Figure 1c). High‐resolution synchrotron PXRD data revealed that the small spherical particles are ZnO nanoparticles left over from MQM synthesis (Figures S5–S7 and Tables S1–S4, Supporting Information). Quantitative phase analysis via the Rietveld method revealed ZnO weight fractions of 21.70(14)%, 16.91(15)%, and 4.56(14)% for GaQM(4), ZnQM(4), and InQM(4), respectively, neglecting the presence of MQ guests due to their extremely low loading (Figure 1d; Table S1, Supporting Information). Bulk powder samples were examined using FTIR and Raman measurements, which did not manifest any distinct peaks for the MQ guests due to their minute loading (Figure S8, Supporting Information). Consequently, to ensure a comprehensive characterization of the guest molecules at the sub‐micron level, different spectroscopic local‐scale probing techniques, such as microRaman and nanoFTIR were conducted (Figure 1e‐h; Figures S9–S14, Supporting Information).^[^ ^29^, ^30^ ^]^ To gauge the locations of the guests in the crystals, we probed different local regions of the crystals using microRaman and observed specific regions (highlighted portions) displaying characteristic bands for the guests, thus affirming their embedment within the host framework in the composite (Figure 1e,f; Figures S9–S11, Supporting Information). To gain more profound insights into the local regions of the composite, we conducted scattering‐type scanning near‐field optical microscopy (s‐SNOM) imaging and nanoFTIR characterization of a single composite crystal (Figure 1g,h, Figures S12–S14, Supporting Information). We have measured the local IR spectra of a 20 nm spot on the fragment of GaQM(4) crystals.^[^ ^29^ ^]^ The signature vibrational bands of the GaQ also appear in the nanoFTIR spectra (highlighted area), providing compelling evidence for the guests' encapsulation into the frameworks (Figure 1g,h; Figure S12, Supporting Information). The optical properties of the composites were further investigated. The UV–vis diffuse reflectance spectroscopy (DRS) spectrum of the composite materials exhibits distinct absorption peaks corresponding to the confined guests, also providing evidence for their existence within the composite materials (Figure S15, Supporting Information). MQM(4) composites exhibit sky blue color with emission at 468 nm upon excitation at 400 nm. Despite the higher loading of impurities, such as ZnO nanoparticles, the GaQM(4) composite exhibited a greater photoluminescence quantum yield (PLQY) in comparison to the ZnQM(4) and InQM(4) composites although ZnO is not taking part in the PLQY (**Figure** **2**a; Table S5, Supporting Information).

### 2.2 Acetone Vapor Detection in the Diabetic Range

The composite's remarkable resistance to hydrolysis and luminescent characteristics motivated us to investigate its potential for gas phase sensing applications. As a preliminary screening test, GaQM(4) composite powder was subjected to acetone vapor, resulting in a rapid enhancement of the powder's luminescent intensity (Figure S16, Supporting Information). Enthused by this observation, we sought to investigate the extensive vapor phase acetone sensing behavior of luminescent GaQM(4) composite using a standard fluorescence spectrometer (FS‐5 Edinburgh Instruments) equipped with a customized gas flow cell.^[^ ^21^, ^31^ ^]^ The acetone sensing experiments were first conducted separately using the powder sample of hosts (pristine MAF‐5), guests (GaQ and ZnQ), and physical mixing of hosts and guests as well as for composites (Figure 2b). No increment of luminescent intensity was observed for GaQ, MAF‐5, or the physical mixture of GaQ and MAF‐5, indicating there is no discernible sensing of acetone vapor (Figure S17, Supporting Information). Nonetheless, the composites demonstrate prompt turn‐on sensing, implying that composite materials possess exceptional sensitivity to acetone vapor (Figure 2c,d). This experimental finding indicates the important role played by the MAF‐5 framework in the composites used for acetone vapor detection. Material can detect up to ≈200 ppb of acetone, as verified by the FS‐5 instrument (Figure S18, Supporting Information). This detection threshold is much lower than the diabetic point (1800 ppb) and significantly surpasses the detection limit of other luminescent MOF‐based materials reported to date (Table S6, Supporting Information).^[^ ^21^, ^32^, ^33^, ^34^ ^]^ Intriguingly, it can also self‐activate within a mere three‐minute interval at room temperature, thereby conferring a notable benefit for expeditious deployment as a reusable sensor (Figure 2c; Figure S19, Supporting Information).

Achieving selectivity for acetone vapor over other competing VOCs in gas phase sensing experiments based on the fluorescent method has been a long‐standing challenge. The composite's selectivity toward acetone was assessed through its exposure to water vapor and a range of interfering VOC mixtures. The GaQM(4) composite demonstrated remarkable selectivity toward acetone in the diabetic range while exhibiting no response to other interfering vapors (Figure 2e; Table S7, Supporting Information).^[^ ^11^, ^35^ ^]^ It is noteworthy to mention that there are currently no luminescent materials documented in the literature that demonstrate high selectivity toward acetone vapor (Tables S6 and S8, Supporting Information). Furthermore, selective acetone vapor detection from a mixture of nitrogen (N_2_), oxygen (O_2_), carbon dioxide (CO_2_), water vapor, and alcohol vapor, is relevant for breath monitoring, and no luminescent materials reported can yet detect trace amounts of acetone in such environment (Figures S20–S21, Supporting Information). Notably, the composite material is capable of detecting a minute amount of acetone vapor (<ppm) within the given setting, while retaining its innate sensitivity (Figure 2f; Table S9, Supporting Information). This observation highlights the exceptional potential of the composite for detecting acetone in breath. Furthermore, the detection experiments were carried out utilizing ZnQM(4) and InQM(4) composites, and it was observed that these composites also demonstrate remarkable sensitivity and selectivity toward acetone vapor (Figures S22 and S23 and Tables S10 and S11, Supporting Information). However, the overall performance efficacy in the diabetic range is relatively lower than that of the GaQM(4). The luminescent composites embedded with MQ demonstrated a restricted sensitivity to acetone vapor, the absence of any discernible selectivity, and operate through a turn‐off mechanism.^[^ ^21^ ^]^ In contrast, in this work, all of the luminescent composites exhibit an enhanced sensitivity to acetone vapor along with excellent selectivity through a turn‐on mechanism. These excellent properties were achieved in the luminescent composites by virtue of the distinctive surface features of the MAF‐5 host.

### 2.3 Films And Fibers‐Based Detection in Diabetic Range

**Figure 3:**
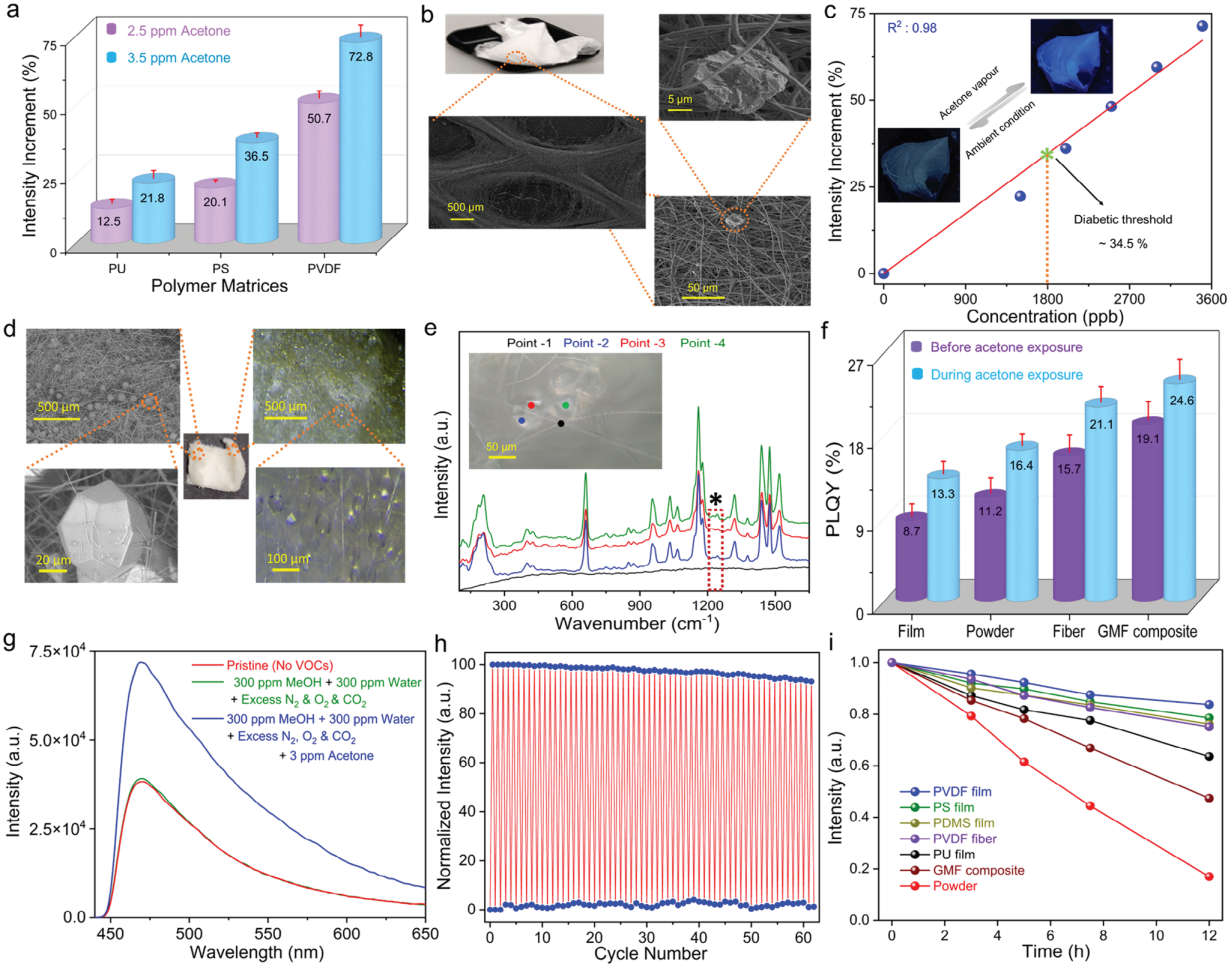
Luminescent sensing of films and fibers. a) Acetone sensitivity by different films such as PU, PS, and PVDF in the diabetic regions where 2.5 wt.% of GaQM(4) was used as a filler in different polymer matrices. b) FESEM micrographs of GaQM(4)/PVDF fibers. c) The calibration profile of acetone sensing in the diabetic range by GaQM(4)/PVDF fibers. The inset photos exhibit the reversibility of acetone sensing. The diabetic threshold was considered at a concentration of 1800 ppb. d) SEM (left) and optical micrograph (right) of the GaQM(4)/GMF composite. e) MicroRaman spectra of a GaQM(4)/GMF composite across the highlighted region in the inset image, where point‐1 was measured on the pristine glass microfiber while other points were measured on the crystals. The asterisk marks the signature band of metal hydroxyquinolate. Note that the probe size of microRaman was ≈1.5 µm × 2 µm. f) PLQY of different materials measured before and during acetone vapor exposure. g) Turn‐on emission spectra of GaQM(4)/GMF composite for acetone selectivity test in the presence of a mixture comprising excess water vapor, alcohol vapor (methanol), N_2_, O_2_, and CO_2_. h) Reusability test of GaQM(4)/GMF composite toward acetone sensing under cyclic tests. i) Photostability test of different luminescent materials subject to UV irradiation.

In pursuit of real‐world applications, different prototypes, including luminescent films/fibers were fabricated utilizing a broad range of polymer matrices, such as polystyrene (PS), polyurethane (PU), and polyvinylidene difluoride (PVDF). We have fabricated multiple luminescent films with these polymer matrices containing varying amounts of GaQM(4) composite while maintaining a consistent film thickness of ca. 160 µm. It is noteworthy that a filler content as low as 5 wt.% has been found to be adequate for detecting ultra‐trace amounts of acetone (Figure S24, Supporting Information). The present investigation reveals that the PVDF films exhibit a notably elevated sensitivity to acetone in comparison to alternative polymer matrices (**Figure** **3**a; Figure S25 and Table S12, Supporting Information). However, the overall performance efficacy in terms of sensitivity decreases when compared to the powder phase, although it is advantageous for practical applications owing to its ease of handling. Due to the unsuitability of powder samples for real‐world applications, we proceeded to fabricate (porous) fibrous samples as they present several advantages (dense) over films in the context of sensing experiments, including high sensitivity and quick response time (Figure 3b; Figures S26–S29, Supporting Information).^[^ ^36^, ^37^ ^]^ The augmentation of luminescent properties in PVDF films does not significantly surpass a filler loading of 5 wt.% (Figure S24, Supporting Information). Hence, in this study, we maintain a consistent filler loading of 5 wt.% into the electrospun PVDF fibers. This choice also presents economic benefits by avoiding the use of a higher filler loading. Additionally, the fabrication of fiber results in an enhancement of the photoluminescent quantum yield (PLQY) of the composite, as compared to both films and pristine powder. The GaQM(4)/PVDF fiber is able to detect selective trace quantities of acetone vapor from the diabetic range (the diabetic threshold is at an intensity increment of 34.5%), thereby demonstrating its potential for practical implementation (Figure 3c; Table S13 and Figure S30, Supporting Information). In order to better determine implications toward real‐world application, we synthesized a luminescent composite utilizing a chemically resistant glass microfiber (GMF) mat (Figure 3d; Figures S31–S37, Supporting Information).^[^ ^38^ ^]^ The microRaman technique was employed to successfully characterize the trace quantity of metal hydroxyquinolate guest in the GaQM(4) composite, as evidenced by the observations made in the luminescent GaQM(4)/GMF composites (Figure 3e; Figure S35, Supporting Information). Distinct local regions of solitary crystals (inset image of 3e), specifically point 2 (red) and point 3 (blue) were examined to identify the presence of guests in the GaQM(4)/GMF composite. The highlighted Raman band observed in the microRaman spectra serves as the distinctive signature for the guest metal hydroxyquinolate. The luminescent characteristics of the GaQM(4)/GMF composite were enhanced in comparison to the pristine powder, films, and fiber phase, thereby conferring advantages for sensitivity testing experiments (Figure 3f, Supporting Information). Similar to the PVDF fibers, the GaQM(4)/GMF composite also exhibits heightened sensitivity and exceptional selectivity toward trace quantities of acetone vapor in the diabetic range (Figure S36, Supporting Information). The GaQM(4)/GMF composite exhibits exceptional sensitivity in detecting minute levels of acetone vapor, even when present alongside a gas mixture comprising substantial amounts of N_2_, O_2_, CO_2_, water vapor, and alcohol vapor, which are commonly found in exhaled breath (Figure 3g). This experiment suggests that the materials possess the potential to be utilized for monitoring low‐centration of acetone levels in exhaled breath. Reusability is one of the most desirable properties of any adsorbent for practical application. To assess the reusability of our materials, we conducted multiple consecutive test cycles (62 cycles) to evaluate the efficacy of our developed GaQM(4)/GMF fiber composite in detecting trace amounts of acetone vapor (Figure 3h). The acetone sensitivity was retained even after being reused for over fifty consecutive cycles, rendering it appropriate for long‐term breath monitoring. To the best of our knowledge, no luminous material based on MOFs has been reported that can detect VOCs through a turn‐on fluorescence approach over such a large number of cycles. Insufficient water stability and photostability are the two major roadblocks of any luminescent materials toward widespread use in sensing applications. The deterioration or loss of sensitivity of the sensing material over time can cause unreliable sensor readouts, leading to incorrect conclusions and potentially detrimental consequences. GaQM(4)/GMF composite displays comparable sensitivity even after being immersed in water for several days (Figure S37, Supporting Information), suggesting that the resultant composite materials are resilient even in challenging conditions such as those encountered during respiration. A photostability test was conducted for a prolonged duration using a high‐intensity UV light source (150 W) emitting at 365 nm. The inherent fluorescent intensity of the luminescent films and fibers containing GaQM(4) composite exhibits greater resilience to photodegradation compared with their powdered counterparts, due to shielding by polymer matrices (Figure 3i). We found that the photoresistance of PVDF electrospun fibers is relatively lower than PVDF films, potentially due to the presence of substantial porosities within the fiber structure. Taking into account all of its impressive sensing performance qualities, GaQM(4) demonstrates the potential to serve as a reversible luminescent adsorbent for noninvasive diagnosis of diabetes by accurately detecting acetone at ppb‐ppm concentrations in breath samples.

### 2.4 Sensing Mechanism

**Figure 4:**
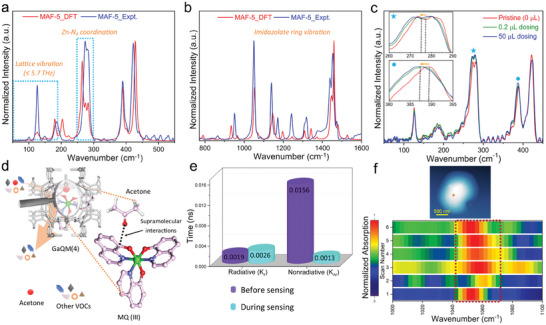
Acetone vapor sensing mechanism. a) Far‐infrared spectrum measured with synchrotron radiation (SR) compared with DFT simulated spectrum. b) Mid‐infrared spectrum measured with synchrotron radiation (SR) compared with DFT simulated spectrum. The IR spectra computed at the PBEsol0‐3c level of theory are scaled by an empirical factor of 0.96 for a better comparison with the experimental findings. c) Different concentrations of acetone gas dosing experiments with SR‐FTIR of GaQM(4). Inset shows the red shifts of the highlighted frequencies. d) Schematic depiction of the interactions between acetone molecule and GaQM(4) composite obtained from DFT calculation of acetone and gallium hydroxyquinolate. e) Radiative and nonradiative decay parameters before and during acetone exposure to the GaQM(4) composite powder. f) NanoFTIR absorption spectra were taken on the highlighted region (six sets of consecutive scans on the same spot) in the image (GaQM(4) crystal) before, during, and after exposure to acetone vapor. Scan‐1 and scan‐2 were measured before and during exposure, while scans 3 to 6 were collected after exposure. The probe size of the nanoFTIR was ≈20 nm.

To gain a detailed understanding of the turn‐on sensing behavior and high selectivity of the material, we conducted comprehensive density functional theory (DFT) calculations for the pristine MAF‐5 framework and synchrotron‐radiation‐based in situ far‐IR dosing experiments (**Figure** **4**; Table S14 and Figure S38, Supporting Information). The predicted IR spectra show a good resemblance with the experimental spectra, as illustrated in Figure 4a,b. In order to account for the “nanocrystal effect”, a common approach was taken whereby a bulk shift of the simulated peaks to lower frequencies was applied (scaling factor 0.96). This is due to the fact that the strengths of real bonds, albeit only slightly, are decreased from those of idealized crystal structures.^[^ ^39^ ^]^ Periodic DFT simulations have been performed with a development version of the ab initio CRYSTAL17^[^ ^40^ ^]^ code by means of the “cost‐effective” PBEsol0‐3c ^[^ ^41^ ^]^ method. The PBEsol0‐3c hybrid composite method has been shown to accurately and efficiently describe inorganic systems and micro‐mesoporous material,^[^ ^42^ ^]^ including MOFs for which structural, vibrational, electronic, and adsorption properties have been predicted with excellent results.^[^ ^43^ ^]^ It is noteworthy that, for the first time, we have successfully identified and assigned all the vibrational modes of the MAF‐5 structure through DFT calculations (Figure 4a,b; Table S14, Supporting Information). The conducted in situ SR far‐infrared dosing investigations have demonstrated that the GaQM(4) composite's Zn‐N vibrational band (277 cm^−1^) in the terahertz region (<8.3THz) experiences a redshift upon exposure of acetone vapor (Figure 4c; Figure S39, Supporting Information). Conversely, the Zn‐N vibrational band of the pristine MAF‐5 remained unaffected (Figure S40, Supporting Information). The red shifting of the vibrational bands of the composite indicates the reduction of the strength of the Zn‐N coordination interactions within the composite, indirectly demonstrating that the acetone molecules strongly interact with Ga(III) hydroxyquinolate in the composites. Further, conventional FTIR measurements demonstrated the distinctive vibrational bands of acetone molecules undergoing a redshift upon exposure to the GaQM(4) composite, indicating that the carbonyl group of acetone interacts significantly with the composite (Figure S41, Supporting Information). Such acetone and metal hydroxyquinolate interactions were further supported by the DFT calculations of acetone and GaQ complex. The DFT calculation reveals the acetone molecules primarily interact with the highest occupied molecular orbital (HOMO) of the GaQ complex (Figure S42, Supporting Information).^[^ ^44^, ^45^ ^]^ Therefore, the combination of DFT and in situ synchrotron‐based far‐IR dosing experiments has implicitly substantiated that the composite undergoes strong interaction with the acetone molecule that restricts the molecular motions of the metal hydroxyquinolate (Figure 4d).

As a consequence of this, detrimental trap states are reduced significantly (>90%) in acetone exposed phase (nonradiative decay; *K*_nr_, 0.0013 ns^−1^) compared to bare materials (*K*_nr_, 0.0156 ns^−1^), and generated excitons are promoted to the radiative path (*K*_r_:*K*_nr_ increases from 0.12 to 2) to improve emission behavior (PLQY increased from 11.2% to 16.4%) (Figure 3e; Figure S43 and Tables S15 and S16, Supporting Information). The distinctive selectivity characteristics of this composite have also been assessed by treating it with different relatively lower boiling‐point VOCs in the dosing experiments. Methanol and dichloromethane (DCM) were respectively employed as the exemplar polar and relatively less polar VOCs. The absence of any discernible shifts in the vibrational bands within the terahertz region in the presence of methanol and DCM suggests a lack of interaction between the composite and these VOCs (Figure S44, Supporting Information). The study revealed a notable alteration of the vibrational bands in the terahertz region subsequent to the introduction of a trace quantity of acetone vapor, thereby validating the exceptional selectivity of the composite toward acetone vapors. The metal hydroxyquinolate is well‐distributed throughout the material, resulting in a strong interaction between the acetone molecules, which is believed to be responsible for the remarkable sensitivity observed in the diabetic range. This is due to the guests being loaded at a significantly low level (Figure S3, Supporting Information). The achievement of exceptional selectivity can be attributed to the distinctive pore surface features of the MAF‐5 frameworks. In addition, nanoFTIR as a nanoscale analytical technique was employed to ascertain the reversibility of the sensing behavior in the local area of a single crystal.^[^ ^31^ ^]^ Repeated measurements were taken at the same location on the crystal prior to, during, and after exposure to acetone gas. The characteristic band of MAF‐5 at 1053 cm^−1^ exhibited a blue‐shift after exposure to acetone vapor, indicating a significant interaction between the imidazolate ring in the frameworks and the acetone vapor (Figure 4f; Figure S45, Supporting Information). The observation of the prompt reversion of the vibrational bands to their original state over time demonstrated the reversibility of the interaction with the host frameworks. Thus, this finding has demonstrated that acetone detection is reversible in nature at a single crystal level.

### 2.5 Portable Turn‐On Optical Sensor Device

**Figure 5:**
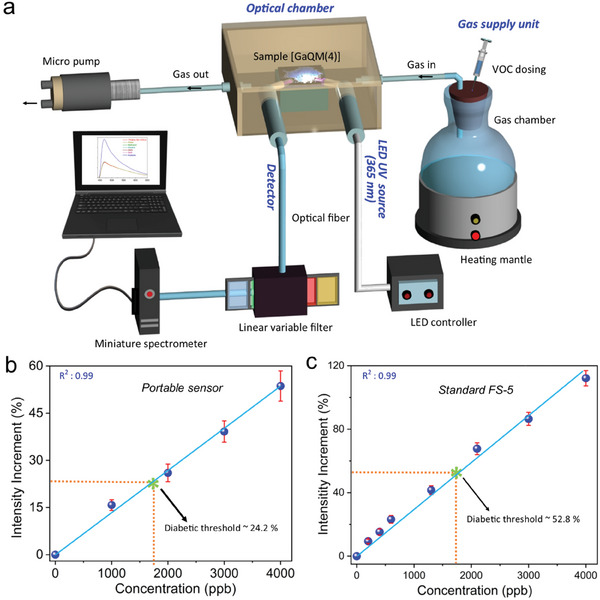
Portable optical sensor device. a) Schematic of the custom‐designed portable optical sensor device. Calibration curve obtained from the acetone vapor sensing experiments using powder samples of GaQM(4) in the diabetic range, b) from the portable sensor device, c) from the industrial standard FS‐5 spectrofluorometer. The diabetic threshold in both graphs was considered at a concentration of 1800 ppb.

Finally, as a proof‐of‐concept, we have designed a turn‐on optical sensor device based on fluorescence method for the detection of trace quantities of acetone from the air utilizing fluorescent method (**Figure** **5**). The prototype portable sensor device consisted of four major components, which are referred to as a gas supply unit, an optical chamber unit (sample chamber), an LED source unit, and a detector unit (Figure 5a; Figure S46, Supporting Information). To improve portability, we note that the future prototype could be miniaturized further by integrating low‐power readout circuits and mini displays, microcontrollers, and wireless communication units (e.g., Bluetooth and Zigbee). Herein, we employed the GaQM(4) powder sample for testing acetone vapor sensing despite its lower luminous properties, this is because we found the powder samples exhibited the greatest sensitivity for the current setup compared to other forms (such as film, fiber, and GaQM(4)/GMP composite). We have contrasted the calibration profiles of the industry standard FS‐5 (spectrofluorometer in laboratories) with the portable optical sensor device for detecting acetone vapor in the diabetic range of interest (Table S17, Supporting Information). The *R*‐square of the calibration curves for both portable sensors device and FS‐5 is 0.99, demonstrating that the newly developed portable device is equally sensitive as the industrial standard FS‐5 instrument. The linear increase in intensity variation is observed with an increase in acetone gas concentration (turn‐on), where the diabetic points are recorded at 24.2% and 52.8% for the portable sensor and standard FS‐5, respectively (Figure 5b,c). This report is noteworthy as it marks the first successful recording of acetone vapor in the diabetic range using a prototype portable optical sensor device by turn‐on fluorescence method. The similar calibration profile of portable sensor device and standard FS‐5 in the diabetic range demonstrates that the prototype device has the capability to swiftly monitor diabetes through the detection of breath acetone. The long‐term durability of the portable sensor device has also been evaluated, a critical factor for its practical use. Even after a period of 18 months, we confirmed that the portable sensor device continues to generate a calibration curve that matches the initial readings (Figure S47, Supporting Information), boding well for real‐world applications under ambient conditions.

## 3 Conclusion

We have rationally designed three luminescent composites that are highly resistant to water and UV light by encapsulating luminescent metal hydroxyquinolates into porous MAF‐5. These newly developed composites demonstrate remarkable sensitivity and selectivity for detecting ultra‐trace acetone vapor, even in the diabetic range, amidst competition from a combination of other VOCs and an excess of water vapor. This finding is a significant advancement from the currently available luminescent materials, which rely solely on turn‐off acetone detection, as our materials offer rapid turn‐on acetone detection to circumvent spurious responses. A combination of computational studies and synchrotron radiation‐based in situ gas dosing experiments uncover the unprecedented acetone sensitivity of the composite. On the account of the extremely high sensitivity of the materials, a one‐of‐a‐kind portable turn‐on optical sensor device has been designed based on fluorescent approach that is as efficient as an industrial standard FS‐5 instrument for acetone vapor detection in the diabetic range. Given the ultra‐trace acetone sensitivity and outstanding selectivity along with a new portable optical sensor device, we anticipate that our findings will leapfrog fluorescent technology for noninvasive diagnosis of diabetes through breath monitoring.

## Conflict of Interest

The authors declare no conflict of interest.

## Author Contributions

S.M. conceived, designed, and developed the project under the supervision of J.C.T.. S.M. conducted all the material synthesis and characterizations. V.K. fabricated the fibers. S.M. analyzed all the data with the help of V.K. and S.R.. S.R. designed the portable sensor under the supervision of J.C.T.. L.F‐B. performed the Rietveld refinement & NMR experiments, and D.S. performed the gas adsorption measurements, both under the supervision of S.H.. L.D. performed the DFT calculations under the supervision of B.C.. S.M. wrote the original draft of the manuscript with input from J.C.T.. All the authors contributed to the final version of the manuscript.

## Supporting information

Supporting Information

## Data Availability

The data that support the findings of this study are available in the supplementary material of this article.
