# Supplementary material for: Unlocking Diabetic Acetone Vapor Detection by A Portable Metal‐Organic Framework‐Based Turn‐On Optical Sensor Device
Source: Adv Sci (Weinh). 2023 Nov 30;11(4):2305070. doi: 10.1002/advs.202305070 (PMC10811499; doi:10.1002/advs.202305070)
Supplement: Supplementary file 1 — Supporting Information [file ADVS-11-2305070-s001.pdf]

ADVANCED SCIENCE
Open Access

# Supporting Information

for *Adv. Sci.*, DOI 10.1002/advs.202305070

Unlocking Diabetic Acetone Vapor Detection by A Portable Metal-Organic Framework-Based Turn-On Optical Sensor Device

*Samraj Mollick, Sujeet Rai, Louis Frentzel-Beyme, Vishal Kachwal, Lorenzo Donà, Dagmar Schürmann, Bartolomeo Civalleri, Sebastian Henke and Jin-Chong Tan**

# Supplementary Information

## Unlocking diabetic acetone vapor detection by a portable metal-organic framework-based turn-on optical sensor device

Samraj Mollick[a], Sujeet Rai[a], Louis Frentzel-Beyme[b], Vishal Kachwal[a], Lorenzo Donà[c], Dagmar Schürmann[b], Bartolomeo Civalleri[c], Sebastian Henke[b], Jin-Chong Tan[a]*

[a]Multifunctional Materials & Composites (MMC) Laboratory, Department of Engineering Science, University of Oxford, Parks Road, Oxford OX1, United Kingdom.

[b]Anorganische Chemie, Fakultät für Chemie & Chemische Biologie, Technische Universität Dortmund, Otto-Hahn Straße 6, 44227 Dortmund, Germany.

[c]Dipartimento di Chimica Università di Torino Via P. Giuria 5, Torino 10125, Italy.

*Corresponding author's e-mail: jin-chong.tan@eng.ox.ac.uk

# Table of Contents

Materials S-2

Materials synthesis S-2 to S-4

Materials characterization and physical measurement techniques S-4 to S-8

Structural characterization of composites S-9 to S-21

Optical characterization S-22 to S-35

Films & fiber phase sensing S-36 to S-47

Vibrational bands assignment of MAF-5 S-48 to S-52

SR-FTIR Measurements S-52 to S-55

Density functional theory (DFT) calculations S-56

Sensing mechanism S-57 to S-60

Portable Sensor device S-60 to S-61

References S-62

# Materials

All the reagents, solvents, and photochromic guests were commercially sourced and used as received. All of the commercially available materials were purchased from Sigma-Aldrich. Fisher Scientific, Fluorochem, and Alfa Aesar depending on their availability.

# Methods

### Materials Synthesis

**MAF-5.** The preparation of a pristine MAF-5 host involves a facile one-step solvothermal reaction. 3 mmol of ethyl imidazole (2EI) was solubilized in 2 mL of *N*,*N*-dimethylformamide (DMF) solvent while 1 mmol of ZnO nanoparticles (size ~20 nm) was mixed with 8 mL of DMF solution. Subsequently, the two solutions were combined and subjected to sonication for a few minutes before being placed in a capped vial and heated to 120 °C for 10 hours, followed by slow cooling for an additional 12 hours. Finally, the mother liquor was decanted, and the products were further washed at least five times with a copious amount of DMF and methanol to remove the excess reactants and dried at 70 °C overnight.

**MQ.** ZnQ was synthesized in 4 mL of DMF by reacting 1:2 molar ratio of $Zn(NO_3)_2{\cdot}6H_2O$, (0.5 mmol) and 8-hydroxyquinoline (8HQ), (1.0 mmol). The stock solution of ZnQ was synthesized by using 5 mmol of $Zn(NO_3)_2{\cdot}6H_2O$ and 10 mmol of 8HQ reacting in 20 mL DMF solutions. GaQ and InQ were synthesized by reacting 1:3 molar ratio of Ga(III) and In(III), (0.5 mmol), and 8-hydroxyquinoline (8HQ), (1.5 mmol) in 4 mL of DMF. The stock solutions of GaQ and InQ were synthesized by reacting 15 mmol of 8HQ with 5 mmol of $Ga(NO_3)_3.{\cdot}H_2O$ and $In(NO_3)_3{\cdot}H_2O$ respectively in 20 mL DMF solutions.

**MQ@MAF-5 (MQM).** The preparation of the luminescent MQM composites involved a one-step solvothermal reaction step where the total solvent of the reaction mixture was fixed to 10 mL. Briefly, 3 mmol of ethyl imidazole (2EI) was solubilized in 6 mL of DMF solvent and added freshly prepared stock solutions of metal hydroxyquinolate to make a total of 10 mL. Finally, 1 mmol of ZnO nanoparticles were added to the mixture and the mixture was sonicated for a few minutes before being placed in a capped vial and heated to 120 °C for 10 hours, followed by slow cooling for an additional 12 hours. Finally, the mother liquor was decanted,

and the products were further washed at least six times with a copious amount of DMF and methanol to ensure that the excess luminescent guest are not left adhered to the MOF surface. Note that the washing step was carried out with simultaneous sonication (2 minutes per solvent, then centrifugation) to further expedite the removal of external guest species. Composites with different concentrations of MQ guests loading were also prepared by following the aforementioned protocol using different amounts of stock solutions of guests where the total reaction mixture remained fixed at 10 mL. When the guests (MQ) stock solutions were 0.5 mL, 1 mL, 2 ml, 4 mL, and 6 mL; the resultant luminescent materials are denoted as MQM(0.5), MQM(1), MQM(2), MQM(4) and MQM(6), respectively.

**Fabrication of thin films.** A variety of photochromic thin films were fabricated by utilizing a wide range of polymer matrices such as polyvinylidene difluoride (PVDF), polystyrene (PS), and polyurethane (PU),. Initially, polymer matrices were prepared using DMF as a solvent for PVDF, PU, and PS matrices. However, since PDMS polymer already exists in liquid state, solvents were not required for its matrices. First, luminescent composites were dispersed homogeneously into different polymer matrices by a combination of sonication (30 min) and magnetic stirring (24 hours). Next, homogeneously mixed polymer-nanocomposites were cast onto a glass substrate by using the doctor blade technique. The thin-film membranes possessed a thickness of approximately 160 µm, with a uniform casting speed of 12 mm/s employed for all films.

**Fabrication of fibers.** The fibers were generated using the electrospinning technique, which involved several steps. Initially, an electrospinning polymer solution was prepared by homogeneously blending of GaQM grounded powder with 13.7 wt.% of PVDF polymer solutions. To create the 13.7 wt.% of PVDF polymer solutions, the PVDF powder was dissolved in a 3:1 solution mixture of DMA and acetone. To fabricate fibers containing a 2.5 wt.% filler loading, 2.5 wt.% of the GaQM grounded powder was utilized compared to the total quantity of polymer utilized. The electrospinning parameters are given in the section below.

**Fabrication of GaQM/GMF composite:** A set of unique luminescent GaQM/GMF composites was synthesized utilizing two different synthesis methods: solvothermal *in situ* growth synthesis at high temperatures and exchange methods (by post-synthetic guest immersion) at room temperature. The solvothermal *in situ* growth synthesis of GaQM/GMF composites was carried out using the same reaction protocol as the solvothermal reaction method, where a pristine mat of glass microfiber (GMF) filters (Whatman Grade GF/D) was

immersed into the reactants mixture and agitated for several minutes before being placed into a capped vial and heated to 120 °C for 10 hours, followed by slow cooling for an additional 12 hours. Finally, the product was washed thoroughly with copious amounts of DMF and methanol solvents until the complete removal of luminescent guests from the surface of the GaQM/GMF composites was achieved. The synthesis protocol for the exchanged GaQM/GMF composites involved a simple *ex situ* exchange of methanolic solution of GaQM ground powder onto pristine mats of GMF at room temperature for 24 hours in stirring conditions.

## Materials characterization and physical measurement techniques

**Powder X-ray diffraction (PXRD).** Powder X-ray diffraction (PXRD) were performed on a Rigaku MiniFlex diffractometer with a Cu Kα source (1.541 Å) at a scan speed of 0.1°/min and a step size of 0.01° in 2$\theta$.

**Attenuated total reflectance Fourier transform infrared spectroscopy (ATR-FTIR).** ATR-FTIR spectra were acquired at room temperature with a Nicolet iS10 FTIR spectrometer with an ATR sample holder.

**Near-field nanospectroscopy.** The AFM height topography and infrared nanospectroscopy (nanoFTIR) were determined using the s-SNOM instrument (Neaspec GmbH), utilizing a platinum-coated AFM probe (Arrow-NCPt, tip radius < 25 nm, 285 kHz) under the tapping mode illuminated by a broadband mid-infrared (MIR) laser source (Toptica). To suppress background contributions, the signal was modulated at the second harmonic for nanoFTIR absorption spectra. Each spectrum was obtained from averaging over 12 individual measurements with an integration time of 14 s, and subsequently normalized to the spectrum of the silicon substrate. In order to eliminate the instrument noise, we removed 200 $cm^{-1}$ of data from both sides of each nanoFTIR plot during the plotting process.

**Raman spectroscopy.** Raman spectra were recorded using a MultiRam FT-Raman spectrometer (Bruker).

**Confocal Raman microscopy (microRaman).** Raman spectroscopy measurements were performed using a confocal Bruker Senterra Raman microscope. The spectra were collected by

setting the following measurements conditions: 20×, laser 532 nm with grating 1200, resolution 3-5 $cm^{-1}$, laser power 25 mW, integration time 5000 ms, scan area 1.5 µm × 2 µm.

**Scanning electron microscopy (SEM):** Crystals and fibers morphologies were examined using a field-emission scanning electron microscope (FESEM $LYRA_3$ GM TESCAN) and Hitachi scanning electron microscope (TM3030 Plus 0865).

**Diffuse reflectance spectroscopy (DRS).** A 2600 UV-Vis spectrophotometer (Shimadzu) was used to measure the absorption spectra.

**Solid-state photoluminescence spectra.** An FS-5 spectrofluorometer (Edinburgh Instruments) was used to characterize the steady-state emission, excitation spectra, quantum yield (QY), and all the vapor phase sensing experiments.

**Gas phase sensing experiments.** The gas phase sensing experiments were recorded employing the FS-5 spectrofluorometer (Edinburgh Instruments) equipped with a custom-built flow cell module. The inlet to the sample cell was connected with a pipe to a sealed 2 L round bottom flask, heated to 50 °C, while the outlet was connected to a Hamilton syringe. Acetone was injected into the flask with a 10 µL Hamilton syringe, and slow pumping of the syringe initiated the gas flow through the cell.

**Nuclear magnetic resonance (NMR) spectroscopy.** $^{1}H$ NMR spectroscopy was performed on digested MQM(4) samples with Bruker DPX-300, DPX 500 or Agilent DD2 500 spectrometers. The solid samples were digested in a mixture of deuterated dimethylsulfoxide (DMSO-$d_6$, 0.5 mL) and DCl/$D_2O$ (35 wt%, one drop, <0.1 mL) prior to the measurement. The data were processed with the MestReNova (v14.2.0) software. Data were referenced to the residual proton signal of DMSO and chemical shifts are given relative to tetramethylsilane.

**Quantitative phase analysis of MQM(4) samples:** PXRD data collection was performed at DELTA (Dortmund, Germany) with a wavelength of 0.4592 Å utilizing a MAR345 image plate detector. The materials were finely ground and filled into borosilicate capillaries (outer diameter 0.5 mm). Data integration was performed with the DAWN software package[1, 2]. Dual-phase Rietveld fits were performed with TOPAS Academic v6 [3]. Crystallographic data of ZnO (COD ID 1011259, http://qiserver.ugr.es/cod/1011259.html) and MAF-5 (CCDC deposition number 2115207) [4] from the literature were used as starting parameters for the Rietveld refinement. Only the background function, a zero error, the cell parameters, scale

factors and peak parameters (TCHZ peak profile) have been refined. Atomic coordinates and displacement parameters were fixed. H atoms have been removed from the structure of MAF-5 prior to the refinement. The high background level at low 2$\theta$ angles originates from the air scattering of the primary X-ray beam.

**Nitrogen sorption study:** The $N_2$ adsorption and desorption isotherms were measured on a Quantachrome autosorb iQ model 7 instrument. Prior to the measurement, the samples were activated at 90 °C under dynamic vacuum (p ≈ $10^{-5}$ kPa) for 10 hours. During the measurement, the samples were cooled down to 77.35 K using liquid nitrogen. The BET surface areas and total pore volumes were analyzed using Quantachrome ASiQWIN (ver. 5.21) software.

**Electrospinning:** Electrospinning was carried out at 10.5 kV using a DC high-voltage generator. The PVDF solution was supplied to a G19 needle emitter (nozzle) with a blunt tip *via* a syringe pump at a processing rate of 8-20 mL/min. The distance from the collector (aluminium foil or diamond-shaped mesh) to the tip was 16 cm.

**Synchrotron radiation (SR) infrared spectroscopy.** SR FTIR spectra were recorded at the Multimode InfraRed Imaging and Microspectroscopy (MIRIAM) Beamline B22 in the Diamond Light Source (Oxfordshire, UK). IR spectroscopy was performed in vacuum *via* a Bruker Vertex 80 V Fourier Transform IR (FTIR) with an Attenuated Total Reflection (ATR) accessory (Bruker Optics, Germany). The mid-IR spectra were collected using a standard DLaDTGS detector. For the far-infrared spectral range below 700 $cm^{-1}$, a bolometer cooled by liquid helium was used for the detection of terahertz signals. All spectra were acquired with a resolution of 4 $cm^{-1}$ and a scanner velocity of 20 kHz. Pre-processing of spectral data was performed using the OPUS software version 7.2 (Bruker Optics). "Concave rubberband correction" from the OPUS software was applied with 4000 points followed by max-min normalization in the range of 50-680 $cm^{-1}$.

**Transmission FTIR for in-situ acetone gas dosing:** The transmission FTIR spectra were collected using the same interferometer at B22 MIRIAM. In the dosing experiments, a Harrick gas flow cell was used, where the sample was drop casted onto a high-density polyethylene (HDPE) window from a dispersion in methanol. An Al spacer was used between two windows to allow for gas flow through the cell with a chamber volume of 50 µL [5]. The gas flow (5 $cm^3$/min) was achieved with a mass flow controller dosed with liquid acetone in dry carrier nitrogen gas using an SGE 25 µL Hamilton syringe. Between measurements, the sample cell

was flushed with $N_2$ gas at 100 $cm^3$/min to remove acetone from the system. Background spectra were collected by measuring the empty flow cell with dry carrier gas.

**Portable optical sensor:** Optical portable sensor comprised of gas supply unit, optical chamber unit, LED source unit and detector unit. Gas chamber unit comprised glass chamber and heater unit connected with power supply to heat the glass chamber at 50 °C to vaporise the acetone liquid to gas phase. The optical chamber unit comprised of sample on the top of the black substrate, a light emitting diode (LED) port, a detector port, an inlet connected with the gas chamber and an exhaust port to the micro-pump to let the acetone gas out from the chamber with a gas flow rate of 433 $cm^3$ $min^{-1}$. Micropump operated by Arduino Mega 2560, which provide the power supply $V_{dc}$=4-5 volt. The LED (365 nm) source was controlled by LDC-1 single channel LED controller through the optical fibre (QD 400-2-SR-BX). In addition, detector port connected with one end of the optical fibre (EOS-A698777-5) and other end connected with the inlet of a linear variable filter (FHS-LVF) with glass filter (LVF-H). The output signal was carried by optical fibre to the Ocean Insight FLAME-T-UV-VIS-ES miniature spectrometer (200-850 nm). The spectrometer was connected to a computer running the OceanView software for data logging and analysis.

**Density functional theory (DFT) calculations of MAF-5.**

DFT calculations were carried out with the cost-effective PBEsol0-3c method[6] recently developed by some of us and implemented in a development version of the CRYSTAL17 code[7] in its massively parallel version (MPPcrystal)[8] on the ARCHER2 UK National Supercomputing Facility.

In the PBEsol0-3c method, the total energy computed with the PBEsol0 hybrid functional combined with a double-Zeta quality Gaussian basis set, is augmented with two semi-empirical corrections to remove the Basis Set Superposition Error (BSSE) through the geometrical CounterPoise (gCP)[9, 10] approach and to properly describe weak interactions *via* the D3 scheme[11, 12] in its Becke-Johnson rational damping variant.

For the numerical evaluation of the exchange-correlation term a (75,974) pruned grid was employed, corresponding to the XLGRID keyword as used by the CRYSTAL code. Default convergence criteria for geometry optimization and harmonic frequencies calculation were employed. The tolerances for one- and two-electron integrals calculation were set to $10^{-7}$, $10^{-7}$ for the Coulomb and to $10^{-7}$, $10^{-7}$, $10^{-25}$ for the exchange series, respectively. The shrinking factors for the diagonalization of the Kohn-Sham matrix in the reciprocal space were set to 2 for the Monkhorst-Pack net and to 2 for the Gilat net, respectively. A full relaxation of both lattice parameters and atomic positions was performed and the crystal symmetry was maintained during the optimization process. Successively, on the optimized geometry, the harmonic frequencies were computed in the Gamma point using the three-point formula and the IR intensity calculated with the Berry phase approach.

**Theoretical Calculation of interaction between acetone and gallium(III) hydroxyquinolate:** The optimised ground state geometries of the bare and acetone-loaded gallium(III) hydroxyquinolate (acetone$\subset$GaQ system) were obtained by employing density functional theory (DFT) with the Gaussian 09 software package. In this investigation, the B3LYP functional and the LANL2DZ basis set were utilised.

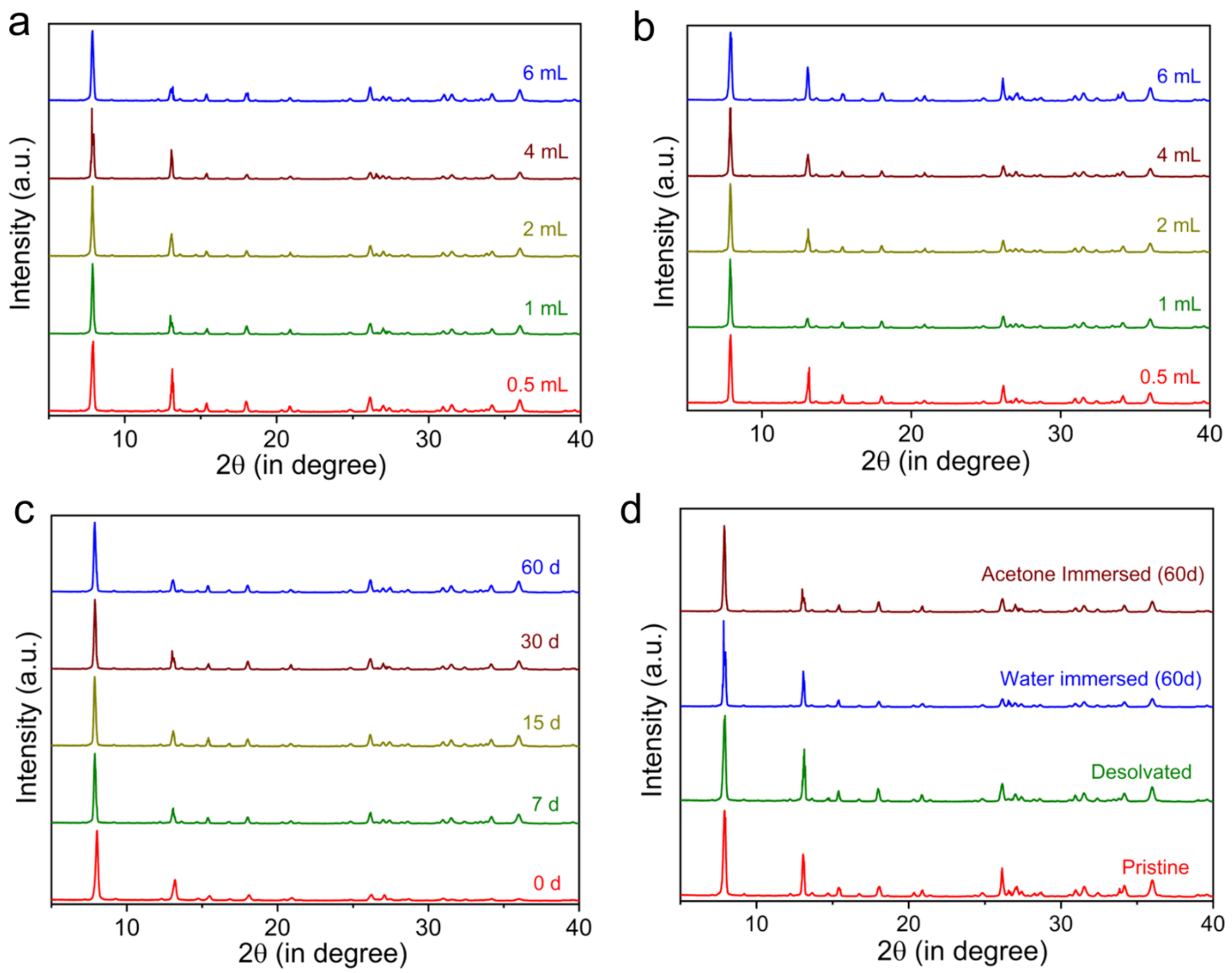


**Fig. S1.** PXRD patterns of (a) GaQM composites with different loading of GaQ guests, **(b)** ZnQM with different loading of ZnQ guests, **(c)** GaQM(4) composite powder sample after immersing in water for certain time, and **(d)** GaQM(4) after treating in different conditions.

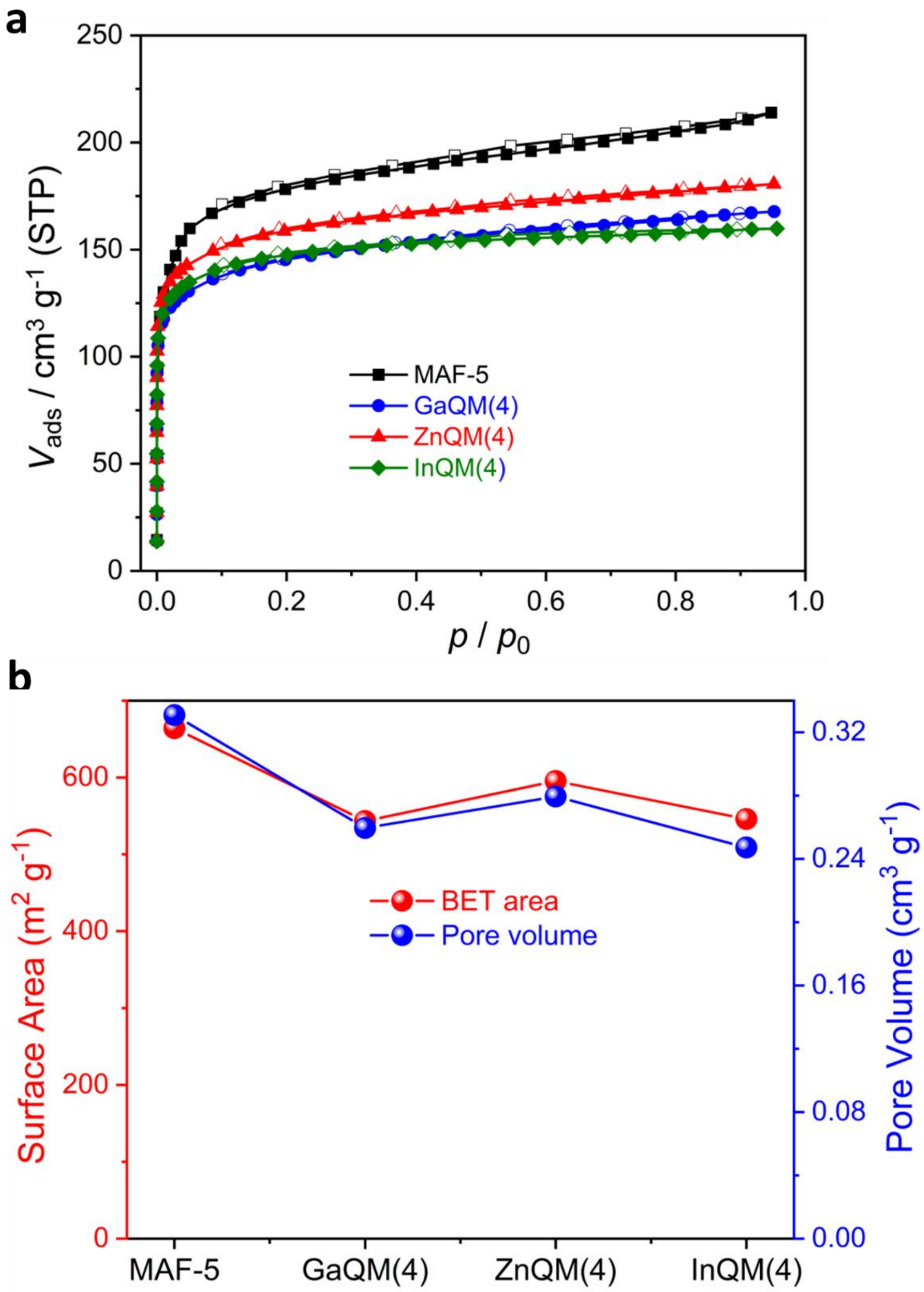


**Fig. S2.** $N_2$ sorption study at 77 K for MAF-5, GaQM(4), ZnQM(4) and InQM(4). **(a)** Sorption isotherm. Adsorption and desorption branches are shown as close and open symbols, respectively. **(b)** Comparison of BET areas and pore volumes derived from the isotherm data plotted in a.

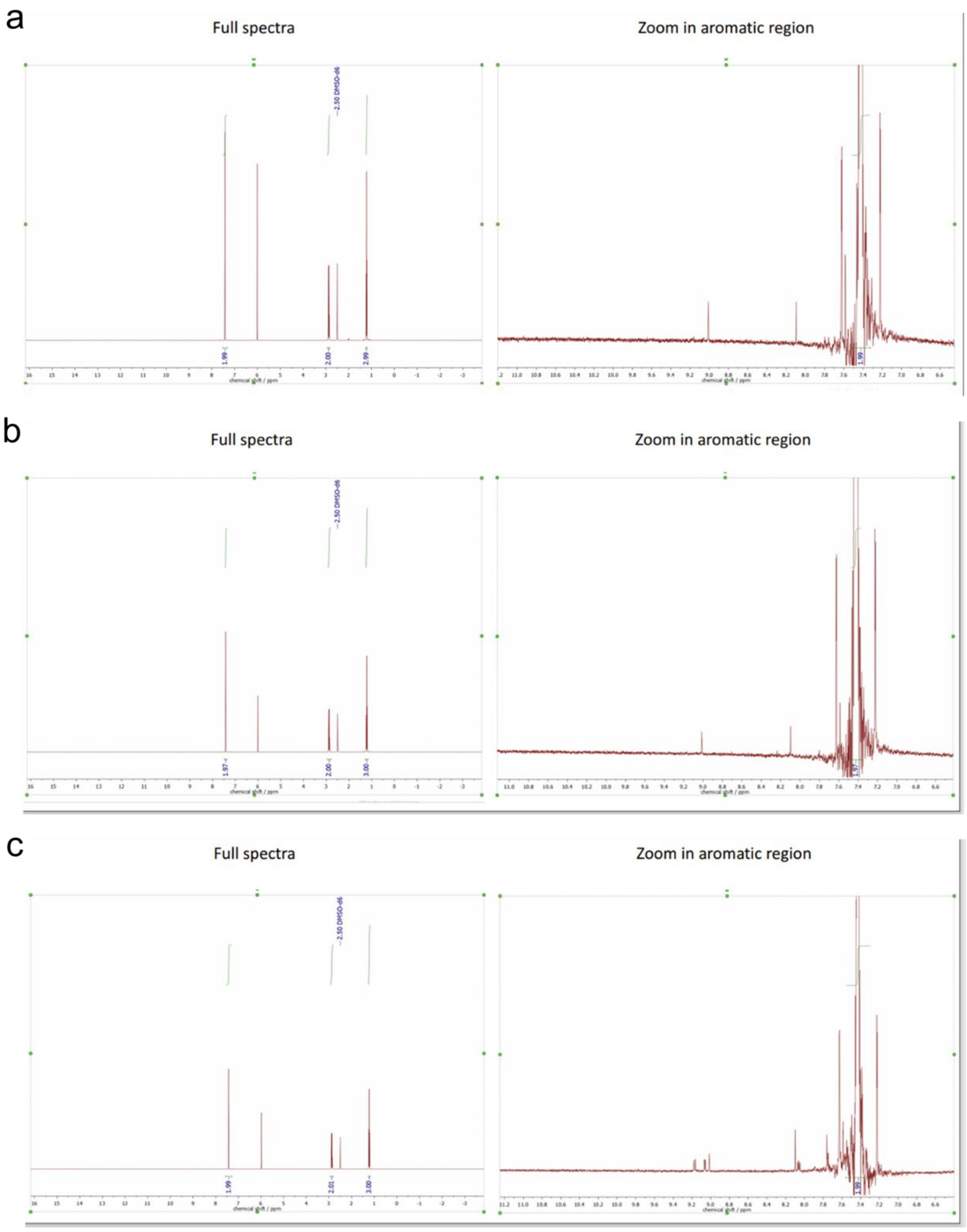


**Fig. S3. (a)** $^{1}H$ NMR spectrum of acid digested ZnQM(4). **(b)** $^{1}H$ NMR spectrum of acid digested GaQM(4) **(c)** $^{1}H$ NMR spectrum of acid digested InQM(4). No characteristics peaks for guests (8-hydroxyquinoline) were noticeable in the full range of $^{1}H$ NMR spectra. However, very weak signals which could be ascribed to the luminescent MQ guest are observable in the range from 7.7 to 9.2 ppm of the spectrum of acid digested InQM(4). The intensity of these signals, however, is even lower than the $^{13}C$ satellite peaks from the aromatic proton signal of 2EI (the linker of MAF-5) located at about 7.4 ppm, indicating an extremely low loading (<1 mol%) of the guests in MAF-5.

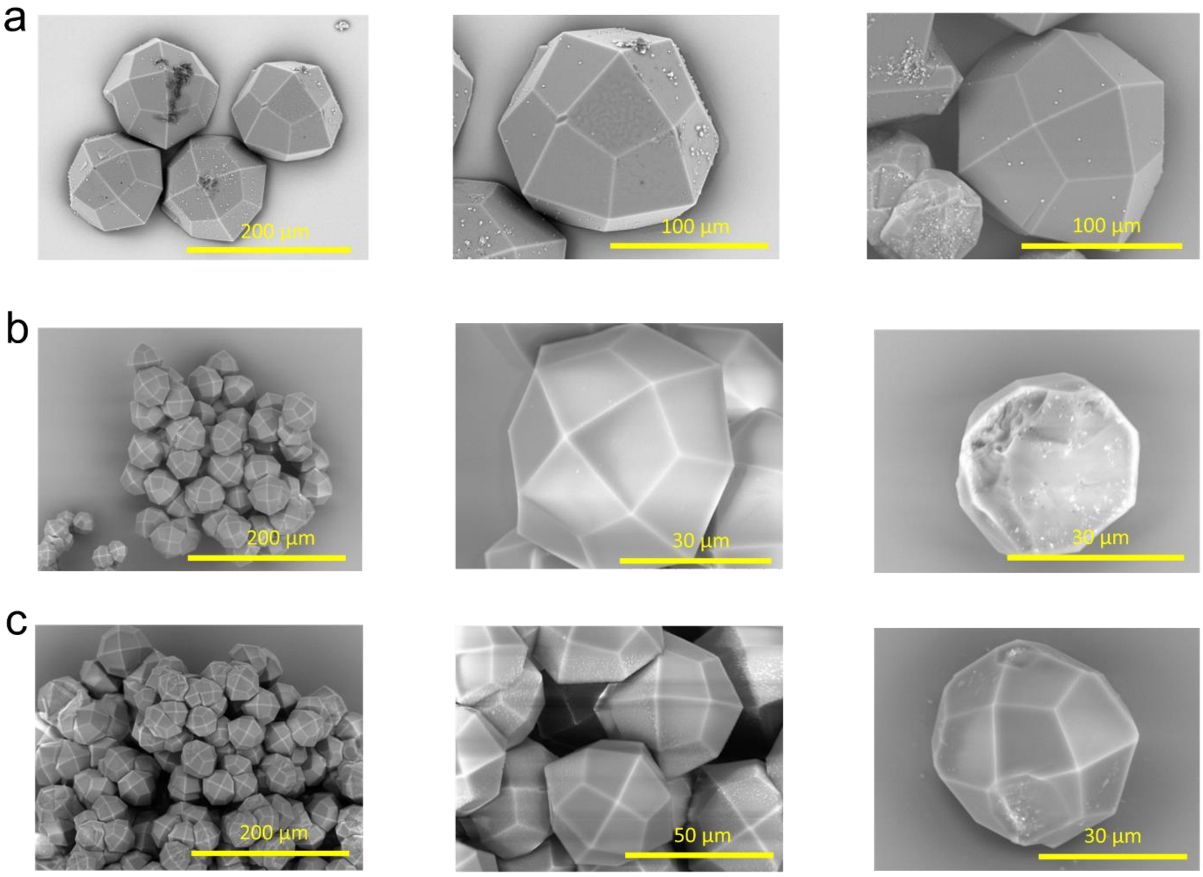


**Fig. S4.** SEM micrographs of **(a)** GaQM(4), **(b)** ZnQM(4), and **(c)** InQM(4).

**Table S1.** Results of the quantitative phase analysis performed by a dual-phase Rietveld fit to high-resolution PXRD data.

| Sample | Fraction of MAF-5 / wt% | Fraction of ZnO / wt% |
|---|---|---|
| GaQM(4) | 78.30(14) | 21.70(14) |
| ZnQM(4) | 83.10(15) | 16.91(15) |
| InQM(4) | 95.44(14) | 4.56(14) |

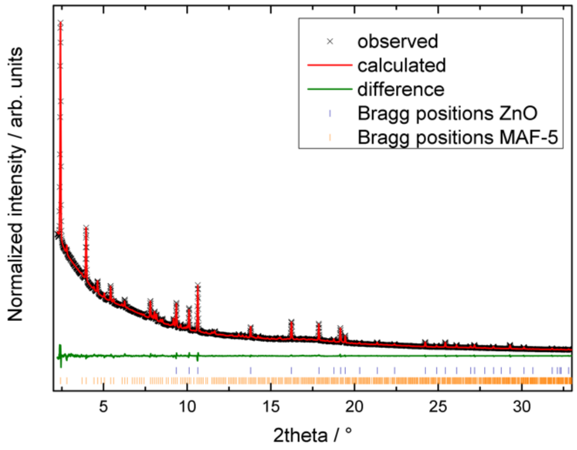


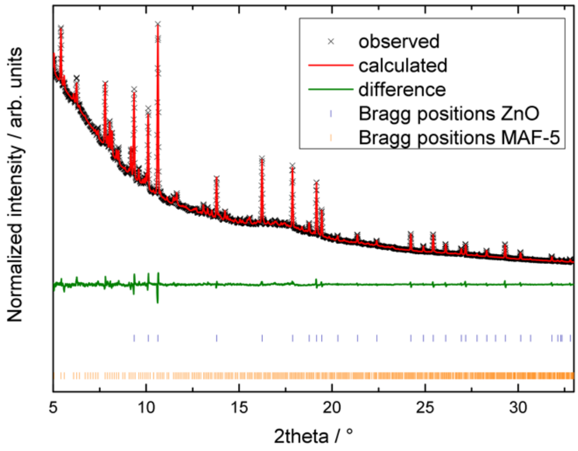


**Fig. S5.** Dual-phase Rietveld fit to the PXRD pattern of GaQM(4) The full pattern is shown on the left and a zoom into the region from 5° to 33° 2theta (λ = 0.4592 Å) on the right.

**Table S2.** Crystallographic parameters of the dual-phase Rietveld fit to the diffraction pattern of GaQM(4)

| | GaQM(4) | |
|---|---|---|
| | MAF-5 | ZnO |
| crystal system | cubic | hexagonal |
| space group | $Ia\bar{3}d$ | $P6_3mc$ |
| $a$ / Å | 26.5792(10) | 3.25178(4) |
| $b$ / Å | 26.5792(10) | 3.25178(4) |
| $c$ / Å | 26.5792(10) | 5.2090(10) |
| $\alpha$ / ° | 90 | 90 |
| $\beta$ / ° | 90 | 90 |
| $\gamma$ / ° | 90 | 120 |
| $R_{exp}$ / % | 3.35 | |
| $R_{wp}$ / % | 1.38 | |
| gof | 0.41 | |

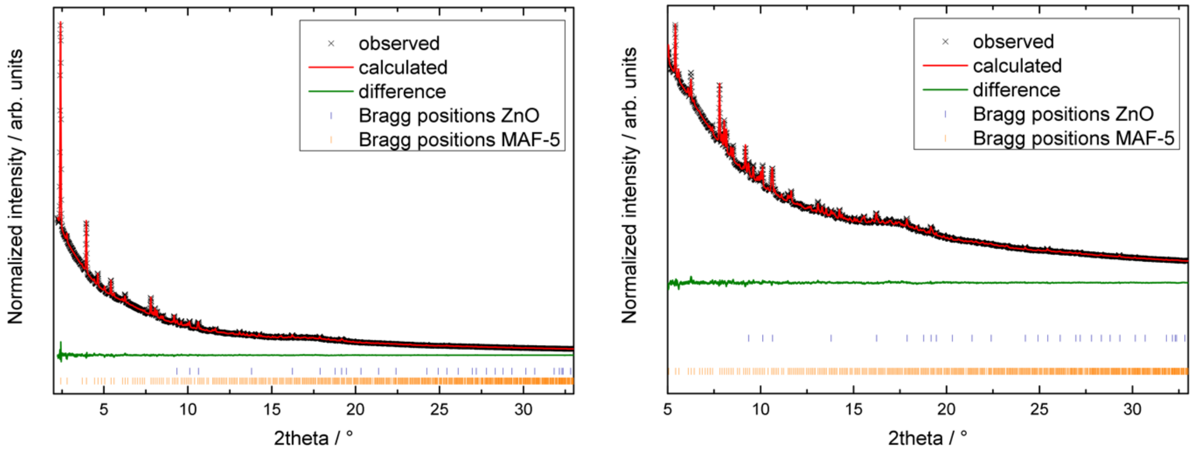


**Fig. S6.** Dual-phase Rietveld fit to the PXRD pattern of InQM(4). The full pattern is shown on the left and a zoom into the region from 5° to 33° 2theta (λ = 0.4592 Å) on the right.

**Table S3.** Crystallographic parameters of the dual-phase Rietveld fit to the diffraction pattern of InQM(4).

| | InQM(4) | |
|---|---|---|
| | MAF-5 | ZnO |
| crystal system | cubic | hexagonal |
| space group | $Ia\bar{3}d$ | $P6_3mc$ |
| $a$ / Å | 26.5597(7) | 3.25071(17) |
| $b$ / Å | 26.5597(7) | 3.25071(17) |
| $c$ / Å | 26.5597(7) | 5.2090(5) |
| $\alpha$ / ° | 90 | 90 |
| $\beta$ / ° | 90 | 90 |
| $\gamma$ / ° | 90 | 120 |
| $R_{exp}$ / % | 3.41 | |
| $R_{wp}$ / % | 0.94 | |
| gof | 0.28 | |

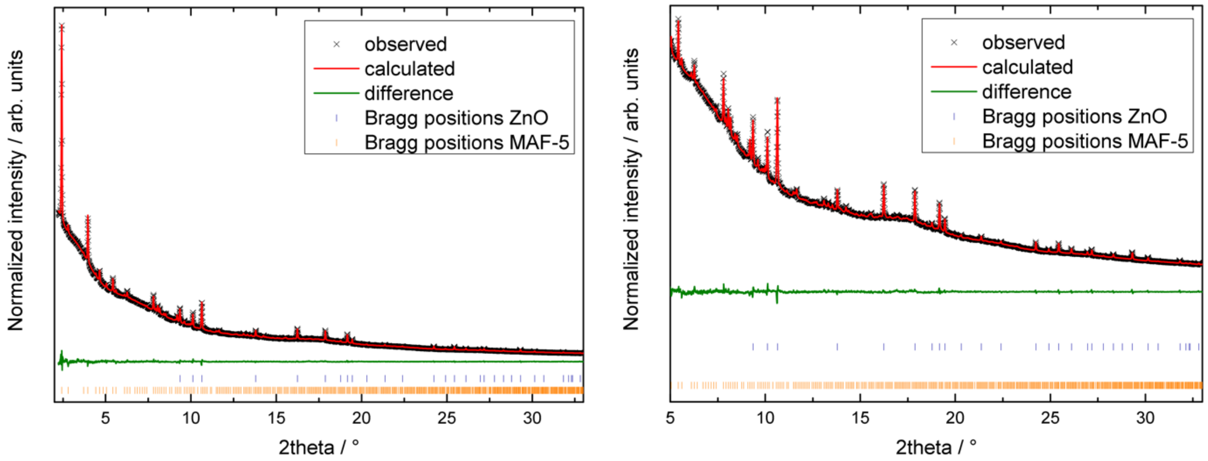


**Fig. S7.** Dual-phase Rietveld fit to the PXRD pattern of ZnQM(4). The full pattern is shown on the left and a zoom into the region from 5° to 33° 2theta (λ = 0.4592 Å) on the right.

**Table S4.** Crystallographic parameters of the dual-phase Rietveld fit to the diffraction pattern of ZnQM(4).

| | ZnQM(4) | |
|---|---|---|
| | MAF-5 | ZnO |
| crystal system | cubic | hexagonal |
| space group | $Ia\bar{3}d$ | $P6_3mc$ |
| $a$ / Å | 26.5704(12) | 3.25106(6) |
| $b$ / Å | 26.5704(12) | 3.25106(6) |
| $c$ / Å | 26.5704(12) | 5.20838(15) |
| $\alpha$ / ° | 90 | 90 |
| $\beta$ / ° | 90 | 90 |
| $\gamma$ / ° | 90 | 120 |
| $R_{exp}$ / % | 3.16 | |
| $R_{wp}$ / % | 0.99 | |
| gof | 0.31 | |

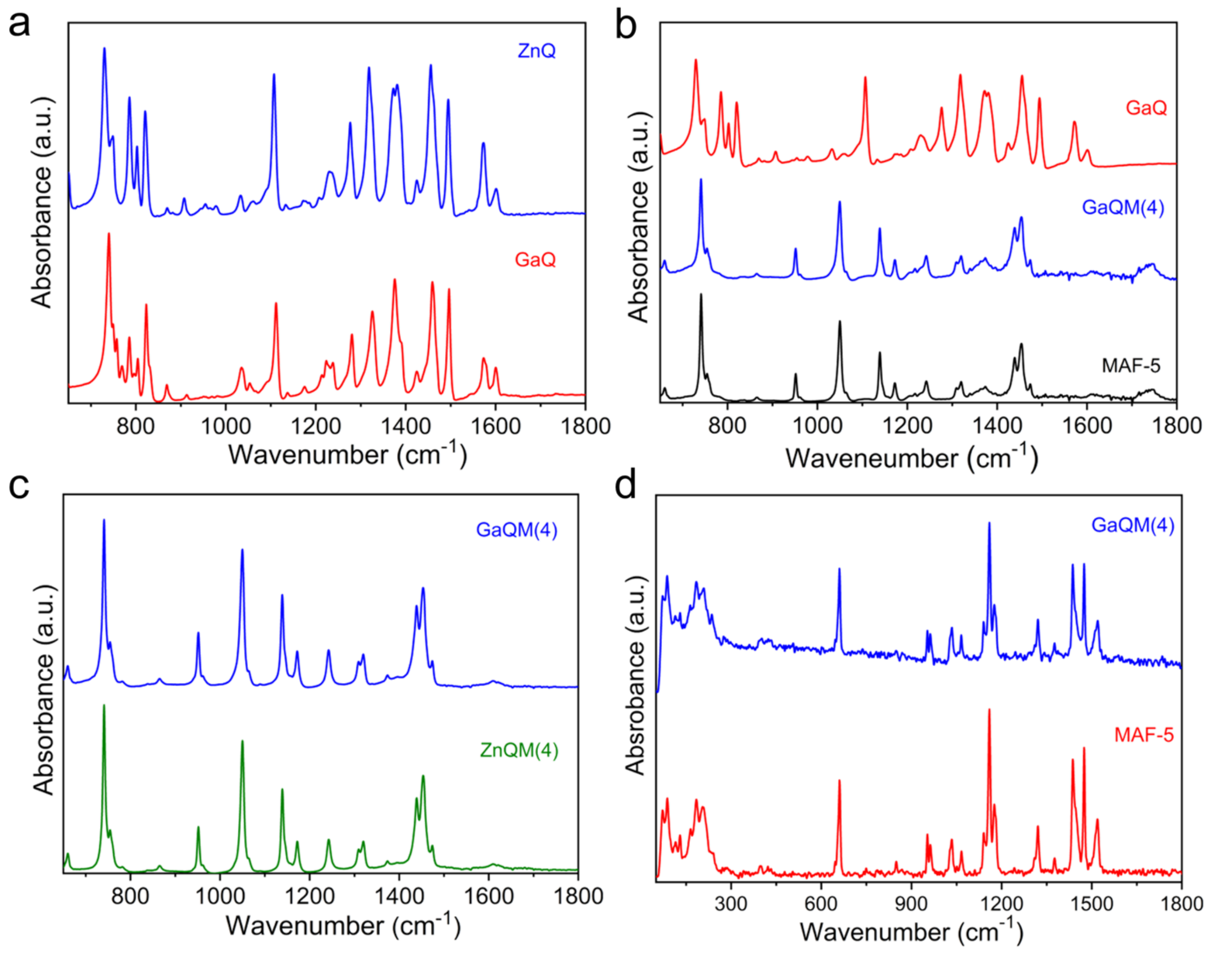


**Fig. S8.** Powder sample characterization. **(a)** FTIR spectra of ZnQ and GaQ. Similar bands in the FTIR spectra demonstrate the identical vibrational bands in both the metal hydroxyquinolate guests. **(b)** FTIR spectra of GaQ, GaQM(4) and MAF-5. **(c)** FTIR spectra of GaQM(4) and ZnQM(4). **(d)** Raman spectra of MAF-5 and GaQM(4). No signature bands for the guests (GaQ and ZnQ) were appear in the composites spectra in both FTIR and Raman spectra confirming extremely low loading (< 1 wt%) of the guests in the composites.

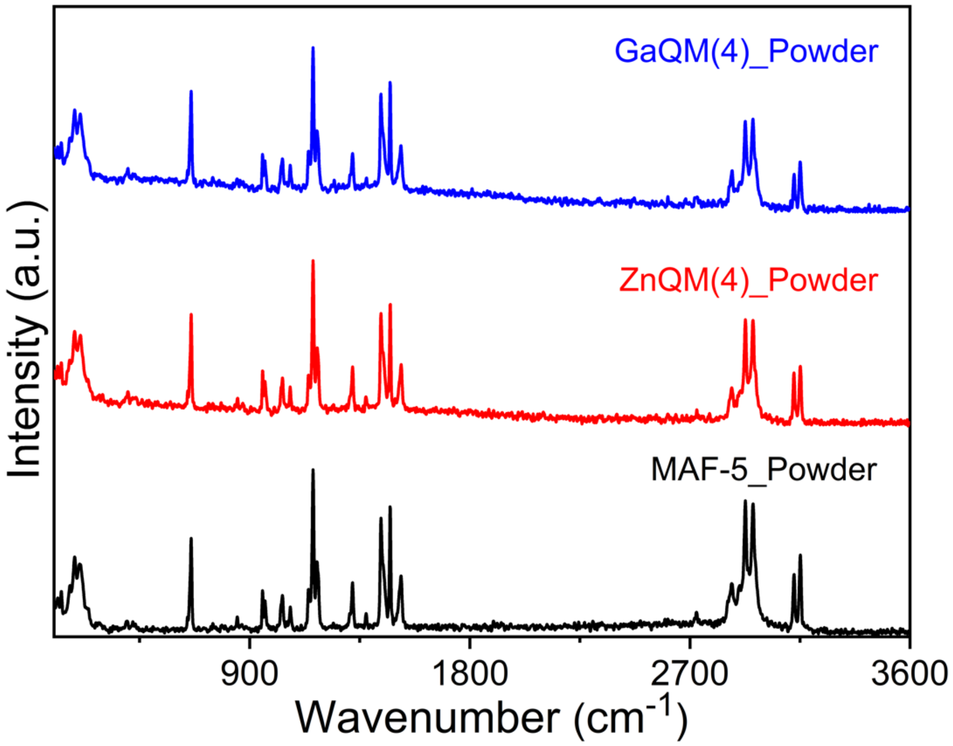


**Fig. S9.** Raman spectra of powder samples of pristine MAF-5, ZnQM(4) composite and GaQM(4) composites. The Raman bands of the composites exhibit a resemblance to the bands of the MAF-5 host. The Raman spectra of the bulk powder phase did not exhibit any discernible signature Raman bands for the guests.

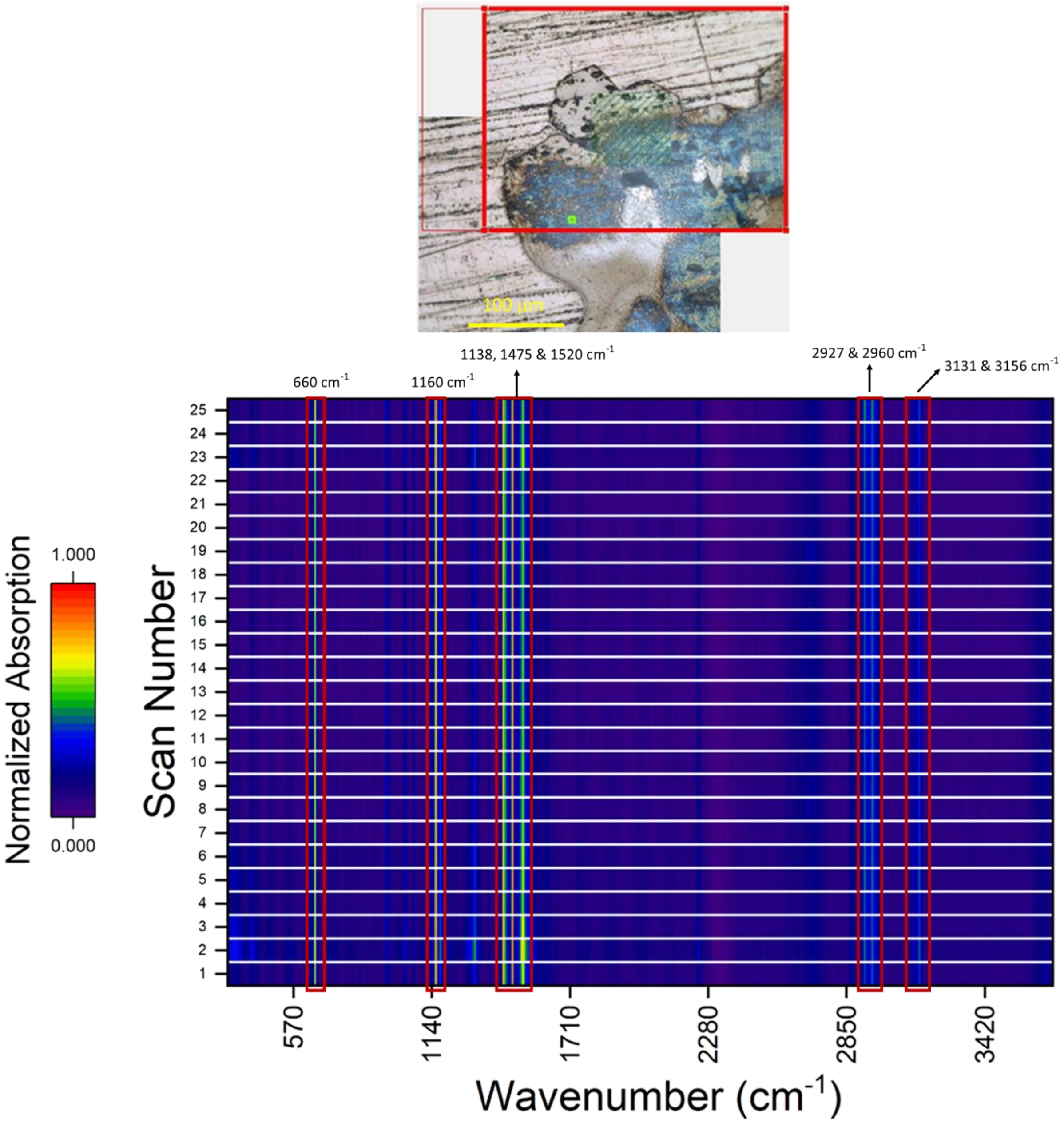


**Fig. S10.** MicroRaman spectra of a GaQM(4) crystals taken across the green highlighted region (line scans in square area) in the image (top). The highlighted portions in the spectra are the signature Raman peaks for the host MAF-5.

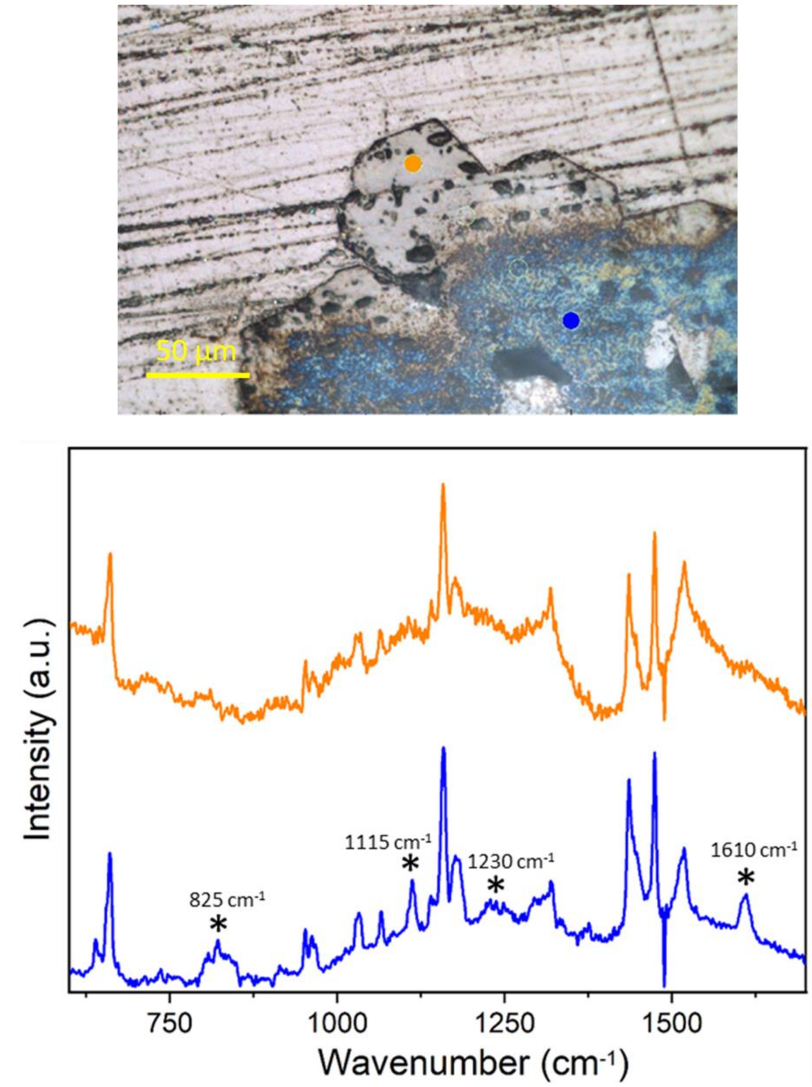


**Fig. S11.** MicroRaman spectra of a GaQM(4) crystals taken in two different spots in the highlighted area. The signature Raman bands of GaQ guests are visible in the microRaman spectrum of the color portions of epoxy mounted crystals while no bands are noticed in the colorless portions. The color portions of the GaQM(4) crystals contained luminescent GQ guests while no guests are present in the colorless portions of the composites crystals.

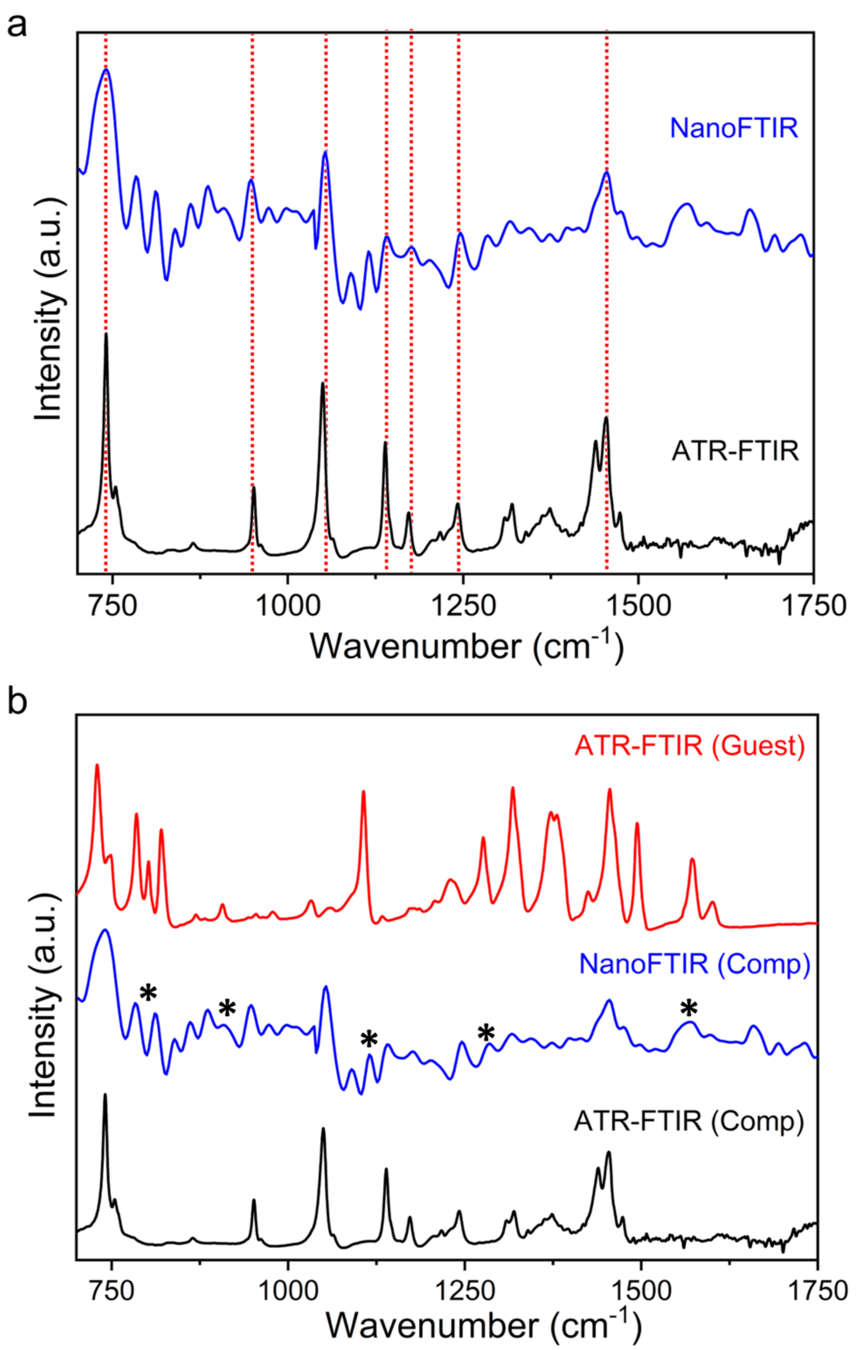


**Fig. S12. (a)** Comparison of vibrational bands for GaQM(4) obtained from conventional ATR-FTIR measurement and nanoFTIR. The presence of signature vibrational peaks for the MAF-5 host corresponds to 741 $cm^{-1}$, 952 $cm^{-1}$, 1050 $cm^{-1}$, 1242 $cm^{-1}$, 1453 $cm^{-1}$, confirming the retention of the host framework at the local region of the crystals. **(b)** FTIR comparison between GaQM(4) (comp) and guests. (*) represents the characteristics signature bands of guest molecules which are absent in the conventional ATR-FTIR measurement.

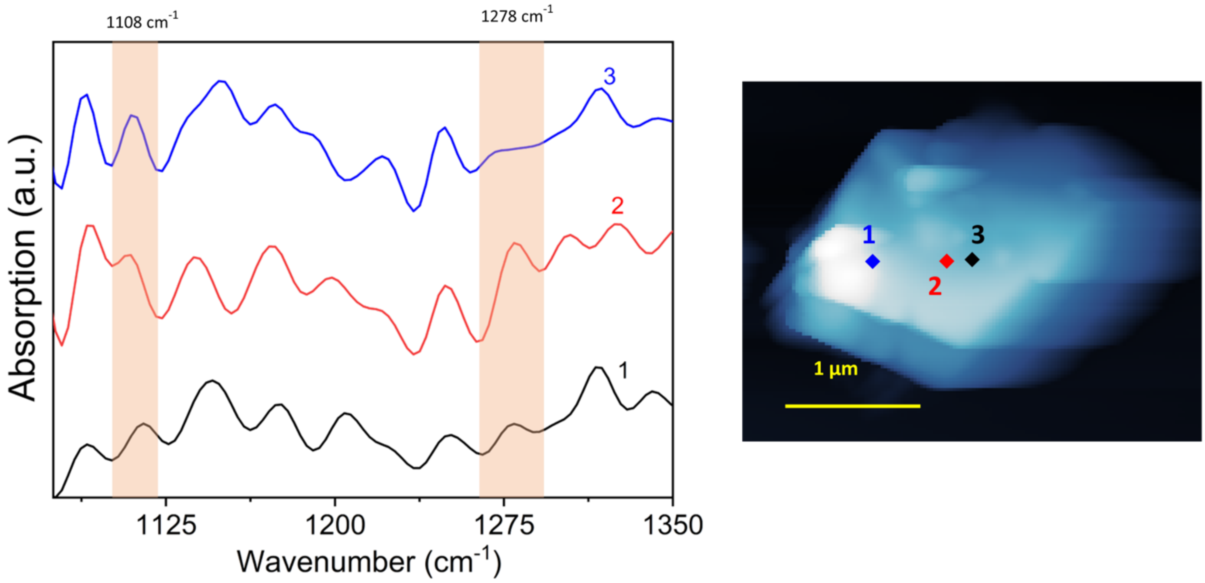


**Fig. S13.** NanoFTIR absorption spectra of a pristine GaQM(4) crystal taken across the highlighted regions in the image. The signature peaks of the GaQ guests are highlighted in the spectra.

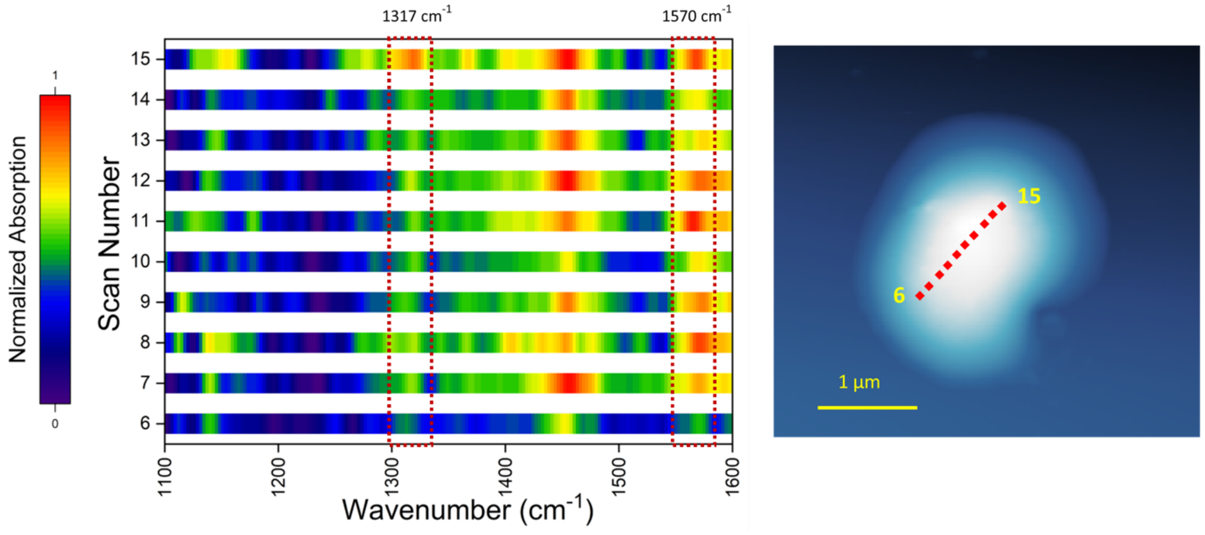


**Fig. S14.** NanoFTIR absorption spectra (heatmaps) of a GaQM(4) composite crystal taken across the highlighted region (scan 6 to 15) in the image. The highlighted peaks are the signature peaks for the metal hydroxyquinolate.

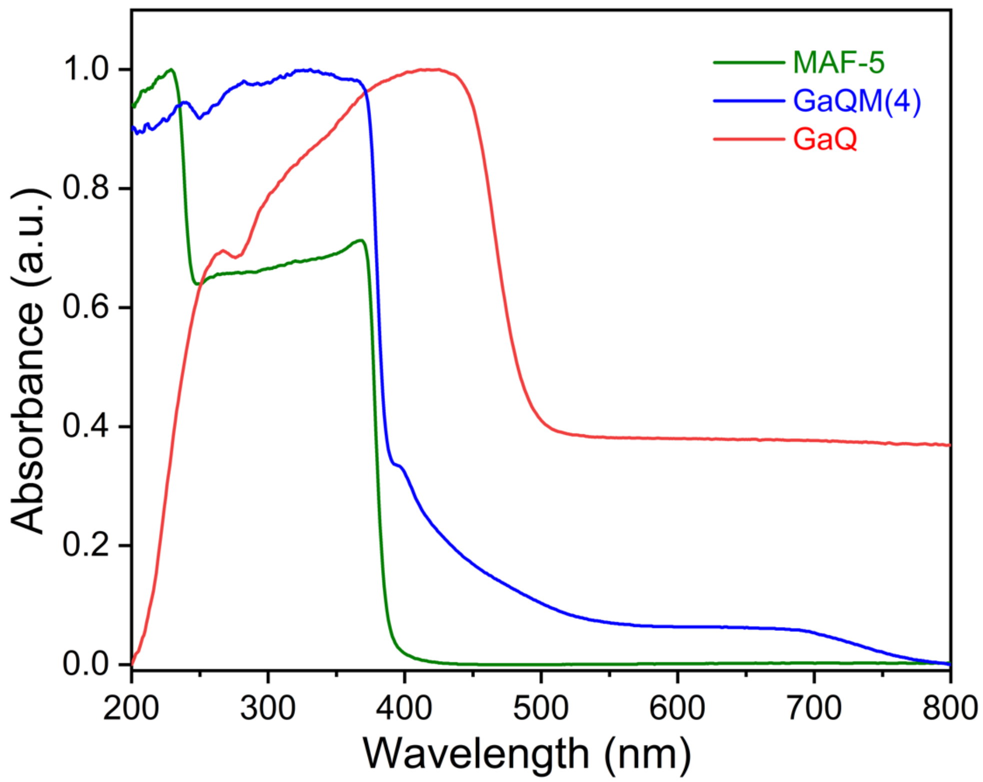


**Fig. S15.** UV-Vis DRS of the MAF-5 (host), GaQM(4) (composite) and GaQ (guest).

**Table S5.** PLQY of different luminescent materials.

| ***Materials*** | ***PLQY (%)*** |
|---|---|
| ZnQM(4) | 9.4 ± 1.4 |
| GaQM(4) | 11.2 ± 1.2 |
| InQM(4) | 7.9 ± 1.3 |

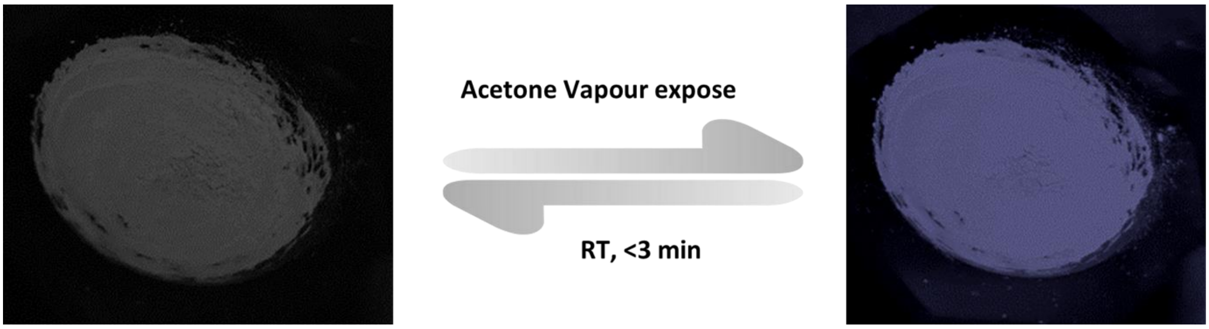


**Fig. S16.** Photos of the GaQM(4) composite under UV light (365 nm). The luminescent property of the material was found to be enhanced by the presence of acetone vapor and subsequently reverted to its initial state at room temperature (RT) within a timespan of three minutes.

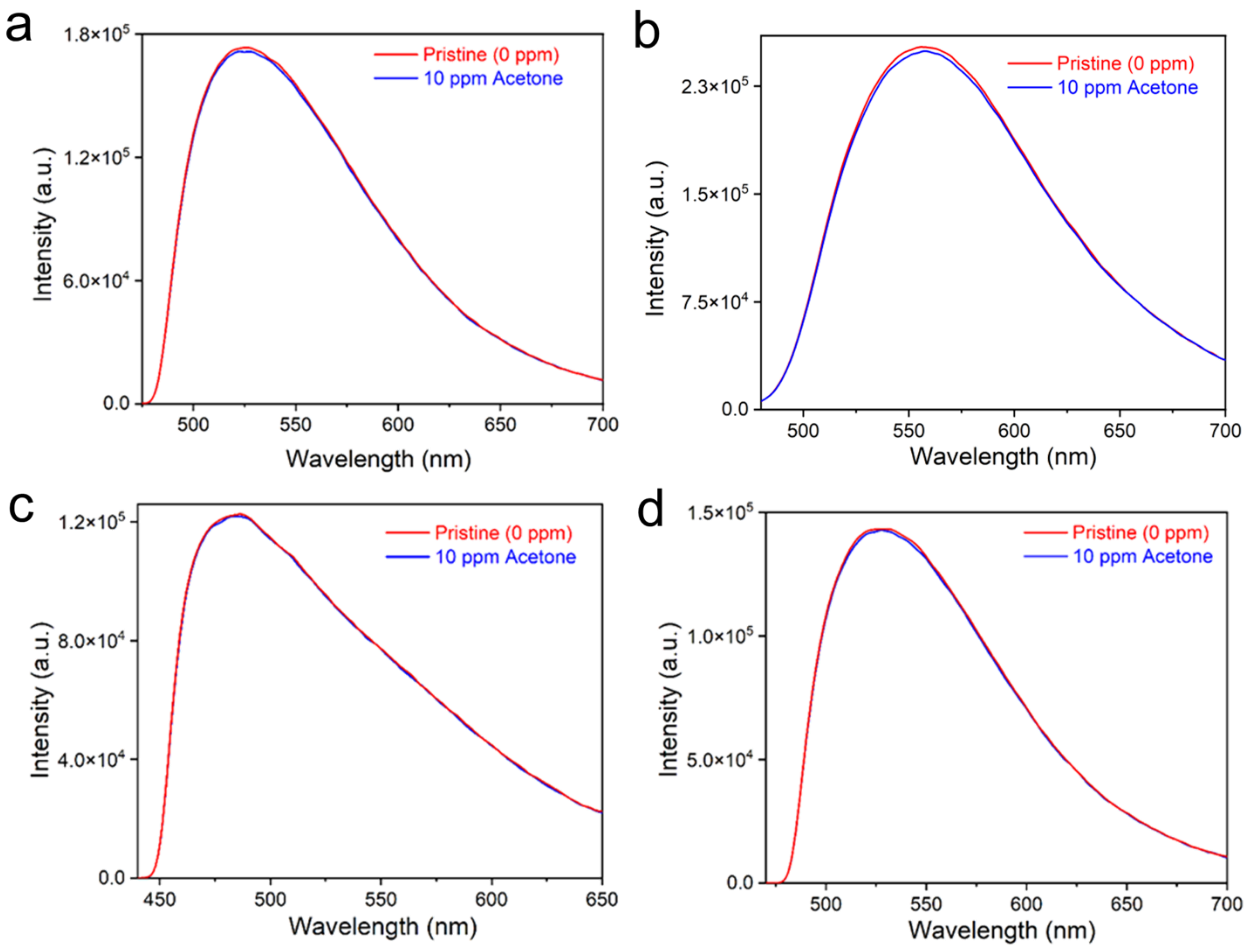


**Fig. S17.** Acetone vapor sensing by **(a)** GaQ powder, **(b)** ZnQ powder, **(c)** pristine MAF-5 powder, **(d)** physical mixing of GaQ and MAF-5 powder. The MAF-5 (host) and GaQ (guest) were physically mixed through a manual process of mechanical blending using a mortar and pestle. The materials did not exhibit turn on sensing in the presence of acetone vapor.

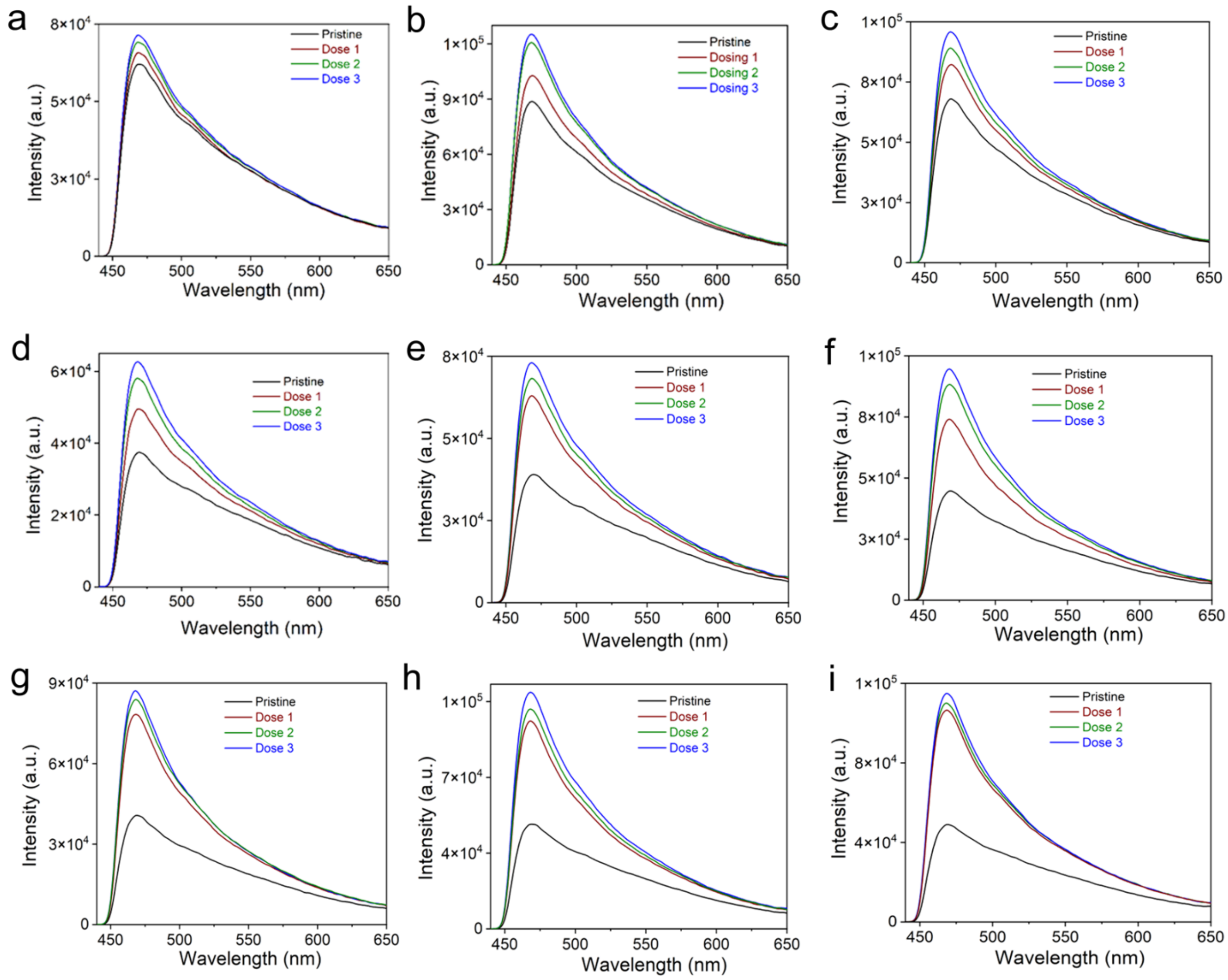


**Fig. S18.** Turn on sensing across different concentrations of acetone vapor by utilizing GaQM(4) powder. **(a)** 200 ppb, **(b)** 600 ppb, **(c)** 1.3 ppm, **(d)** 2.1 ppm, **(e)** 3 ppm, **(f)** 4 ppm, **(g)** 5 ppm, **(h)** 6 ppm, and **(i)** 10 ppm.

**Table S6.** Comparison of this work with other related luminescent MOF-based investigations for the detection of volatile acetone vapor.

| **Materials** | **Type** | **Detection type** | **Selectivity** | **Sensitivity** | **Reference** |
|---|---|---|---|---|---|
| *GaQM(4) Powder* | *MOF composite* | *Turn-on* | *Mixture of 7 VOCs* | *200 ppb* | *This work* |
| *GaQM(4) Fiber/Film* | *Membrane* | *Turn-on* | *Mixture of 7 VOCs* | *500 ppb* | *This work* |
| ZnQ@OX-1 | MOF Composite | Turn-off | NA | 50 ppm | *Adv. Mater.* **2017**, *29*, 1701463. |
| NR~ccZIF-8 | MOF film | Turn-off | NA | 60 ppm | *Adv. Opt. Mater.* **2020**, *8*, 2000961. |
| $[(CH_3)_2NH_2]_2$ $Cd_3(ptptc)_2\}$ | MOF | Turn-off | NA | 86 ppm | *Spectrochim. Acta, Part A* **2021**, *246*, 118962. |
| Octahedron dye @ZIF-8 | MOF composite | Turn-off | NA | Vapor (30 vol%) | *Small* **2016**, *12*, 3302-3308. |

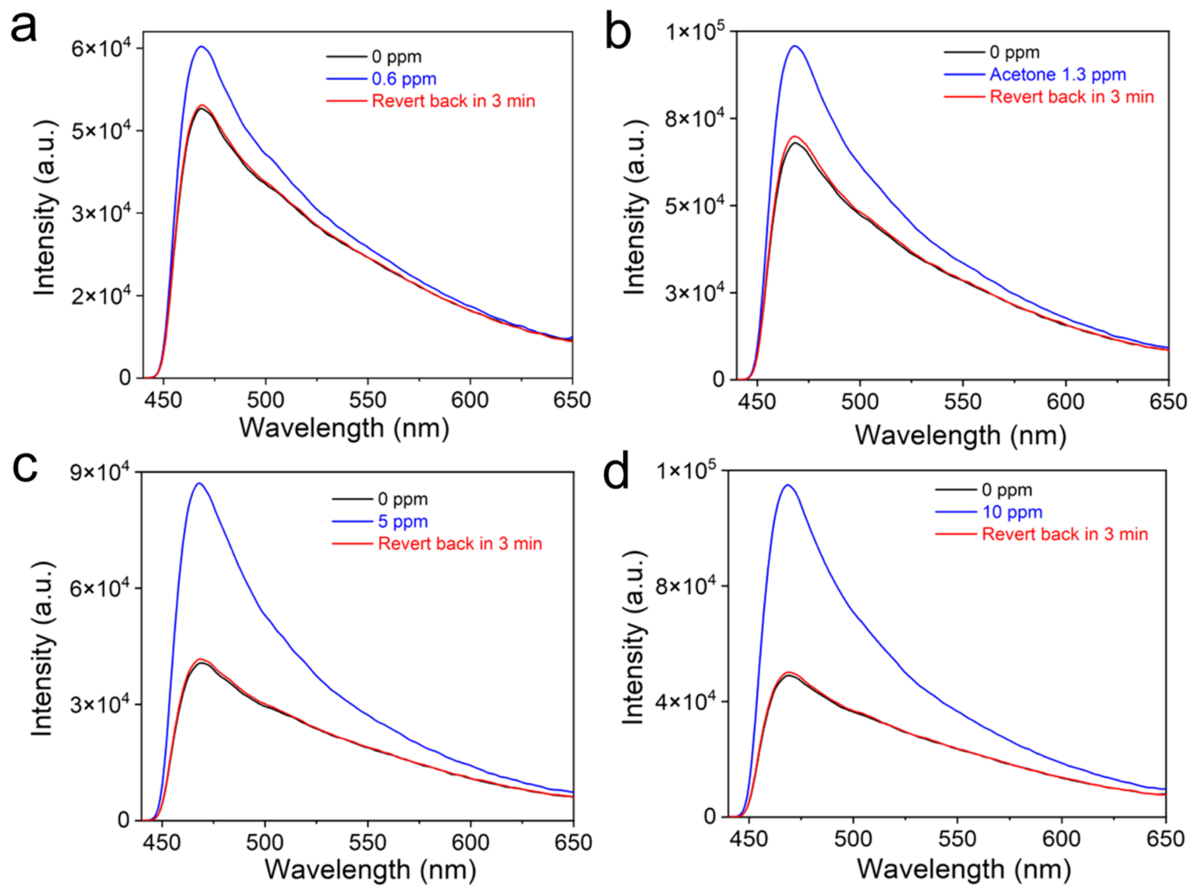


**Fig. S19.** Emission spectra demonstrating all the turn-on sensing across different concentrations of acetone vapor are reversible in nature. **(a)** 0.6 ppm, **(b)** 1.3 ppm **(c)** 5 ppm, and **(d)** 10 ppm.

**Table S7.** Acetone vapor selectivity in the presence of a mixture containing equal concentrations of six different VOCs including water vapor in sub-diabetic ranges.

| **Acetone Concentration** | **Intensity increment (Only Acetone)** | **Intensity increment DMF+ Hexane + DMA + Water + Methanol+$NH_3$+ varying quantities of Acetone (Mixture of 7 components)** |
|---|---|---|
| 0.2 ppm | ~ 9.4 ± 1.6% | ~ 9.8 ± 1.7 % |
| 0.4 ppm | ~ 15.3 ± 1.8 % | ~ 14.4 ± 1.6 % |
| 0.6 ppm | ~ 23.2 ± 2.1% | ~ 23.6 ± 1.9% |
| 1 ppm | ~ 36.2 ± 2.3% | ~ 36.9 ± 2.6 % |
| 2.1 ppm | ~ 67.7 ± 2.9 % | ~66.1 ± 3.4 % |
| 3 ppm | ~ 86.5 ± 3.3 % | ~ 85.4 ± 4.6 % |

**Table S8.** Comparison of this work with other related investigations for the detection of volatile acetone vapor.

| **Materials** | **Type** | **Detection type** | **Selectivity** | **Sensitivity** | **Reference** |
|---|---|---|---|---|---|
| *GaQ@MAF-5 Powder* | *MOF composite* | *Optical* | *Mixture of 7 VOCs* | *200 ppb* | *This work* |
| *GaQ@MAF-5 Fim/Fibre* | *Membrane* | *Optical* | *Mixture of 7 VOCs* | *1500 ppb/400 ppb* | *This work* |
| ZnQ@OX-1 | MOF Composite | Optical | NA | 50 ppm | *Adv. Mater.* **2017**, *29*, 1701463. |
| PtNCS@$SnO_2$ nanofiber | Nanoparticle | Electrical | NA | 1 ppm | *Nanoscale* **2018**, *10*, 13713. |
| $SnO_2$ nanosheet | 2D sheet | Electrical | NA | 200 ppb | *ACS Appl. Mater. Interfaces* **2020**, *12*, 51637-51644. |
| Octahedron dye @ZIF-8 | MOF composite | Optical | NA | Vapor (30 vol%) | *Small* **2016**, *12*, 3302-3308. |
| Cu-MOF | MOF | Electrical | NA | 200 ppb | *Sens. Actuators B: Chem.* **2021**, *329*, 129053 |
| $ZnFe_2O_4$/(Fe-ZnO) | Nanocomposite | Electrical | NA | 20 ppm | *Sens. Actuators B: Chem.* **2020**, *325*, 128783 |
| $In_2O_3/TiO_2$ | Nanowire | Electrical | NA | 10 ppm | *J. Alloys Compd.* **2017**, *696*, 655e662. |
| $In_2O_3$/Au | Hybrid structure | Electrical | NA | 50 ppm | *Sens. Actuators B: Chem.* **2012**, *161*, 178-183. |
| $In_2O_3$/Au | Nano rod | Electrical | NA | 0.1 ppm | *Sci. Rep.* **2015**, *5*, 10717-10814. |
| $In_2O_3$-Pt | Nanoparticles | Redox | NA | 10 ppb | *Sens. Actuators B: Chem.* **2016**, *230*, 697-705 (2016) |
| HKUST-1 | MOF | Mechanical | NA | 2930 ppm | ECS Trans. **2013**, *50*, 469 (2013) |

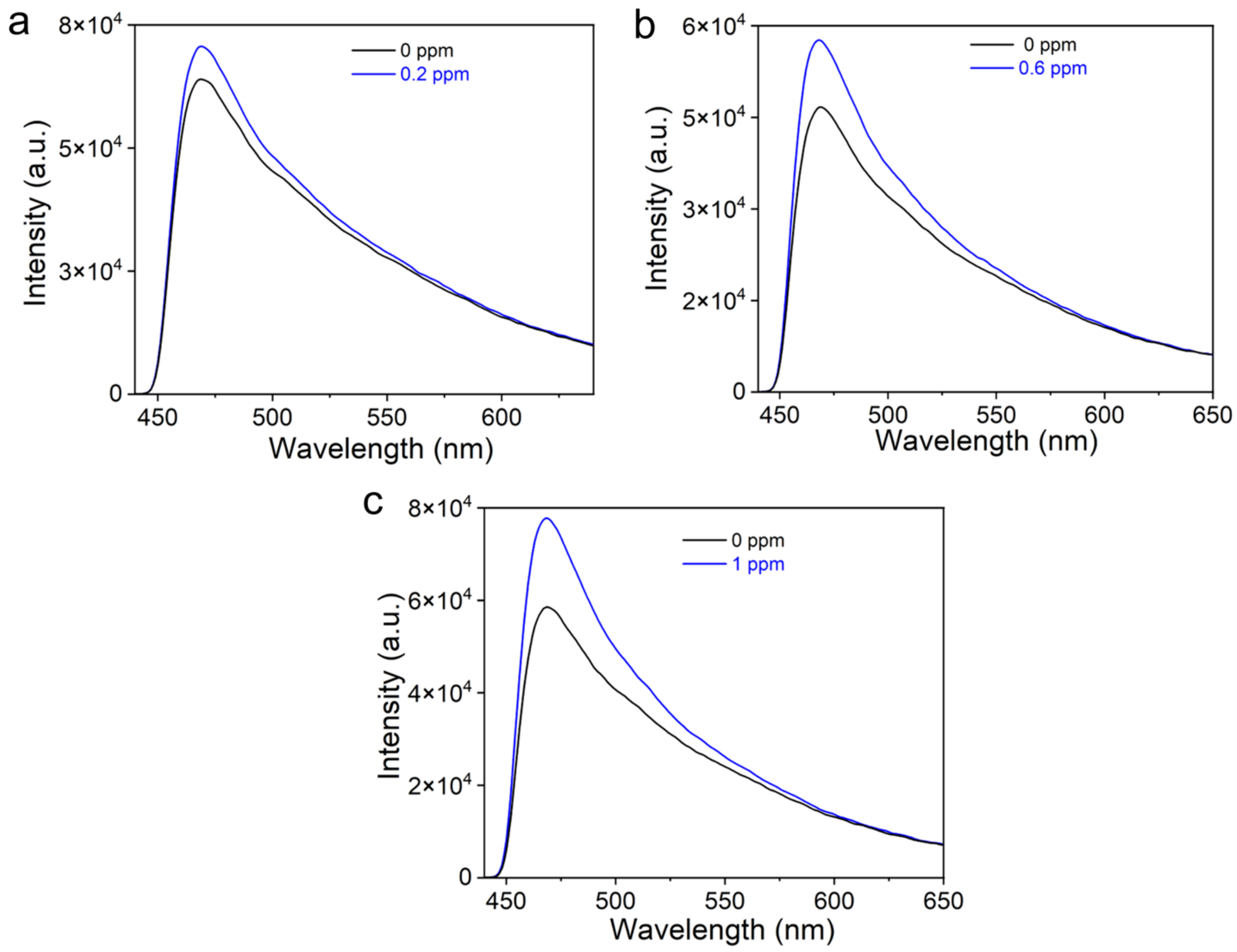


**Fig. S20.** Turn-on sensing of different concentrations of acetone vapor in the presence of a mixture containing 100 ppm water vapor, 100 ppm methanol vapor in the sub-diabetic range. **(a)** 0.2 ppm, **(b)** 0.6 ppm and **(c)** 1 ppm.

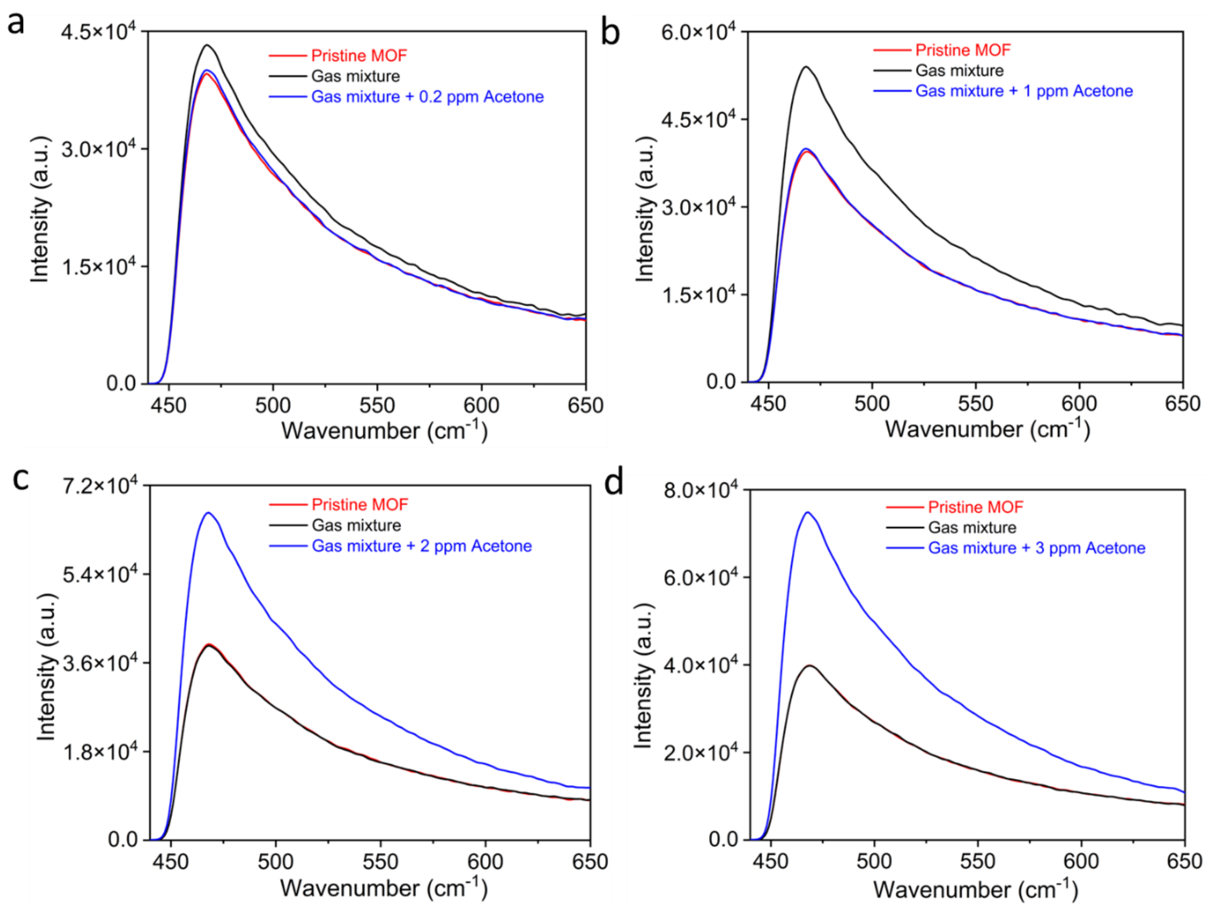


**Fig. S21.** Turn-on sensing of different concentrations of acetone vapor in the presence of a gas mixture comprised of 100 ppm water vapor, 100 ppm methanol vapor and excessive $N_2$, $O_2$ and $CO_2$. The concentrations of acetone used for dosing were as follows: **(a)** 0.2 ppm, **(b)** 1 ppm, **(c)** 2 ppm, and **(d)** 3 ppm.

**Table S9.** Acetone vapor selectivity of GaQM(4) powder was examined in the presence of a mixture containing mixture containing 100 ppm water vapor and 100 ppm methanol vapor.

| **Acetone Concentration** | **Intensity increment (Only Acetone)** | **Intensity increment 100 ppm Water + 100 ppm Methanol + varying quantities of Acetone (Mixture of three components)** |
|---|---|---|
| 0.2 ppm | ~ 9.4 ± 1.3% | ~8.8 ± 1.1% |
| 0.4 ppm | ~ 15.3 ± 1.9% | ~ 15.1 ± 1.8% |
| 0.6 ppm | ~ 23.2 ± 2.4% | ~ 22.2 ± 2.2% |
| 1 ppm | ~ 36.2 ± 2.8% | ~ 35.9 ± 2.6% |
| 2.1 ppm | ~ 67.7 ± 3.7% | ~66.3 ± 3.9% |
| 3 ppm | ~ 86.5 ± 4.1% | ~ 85.7 ± 4.4% |

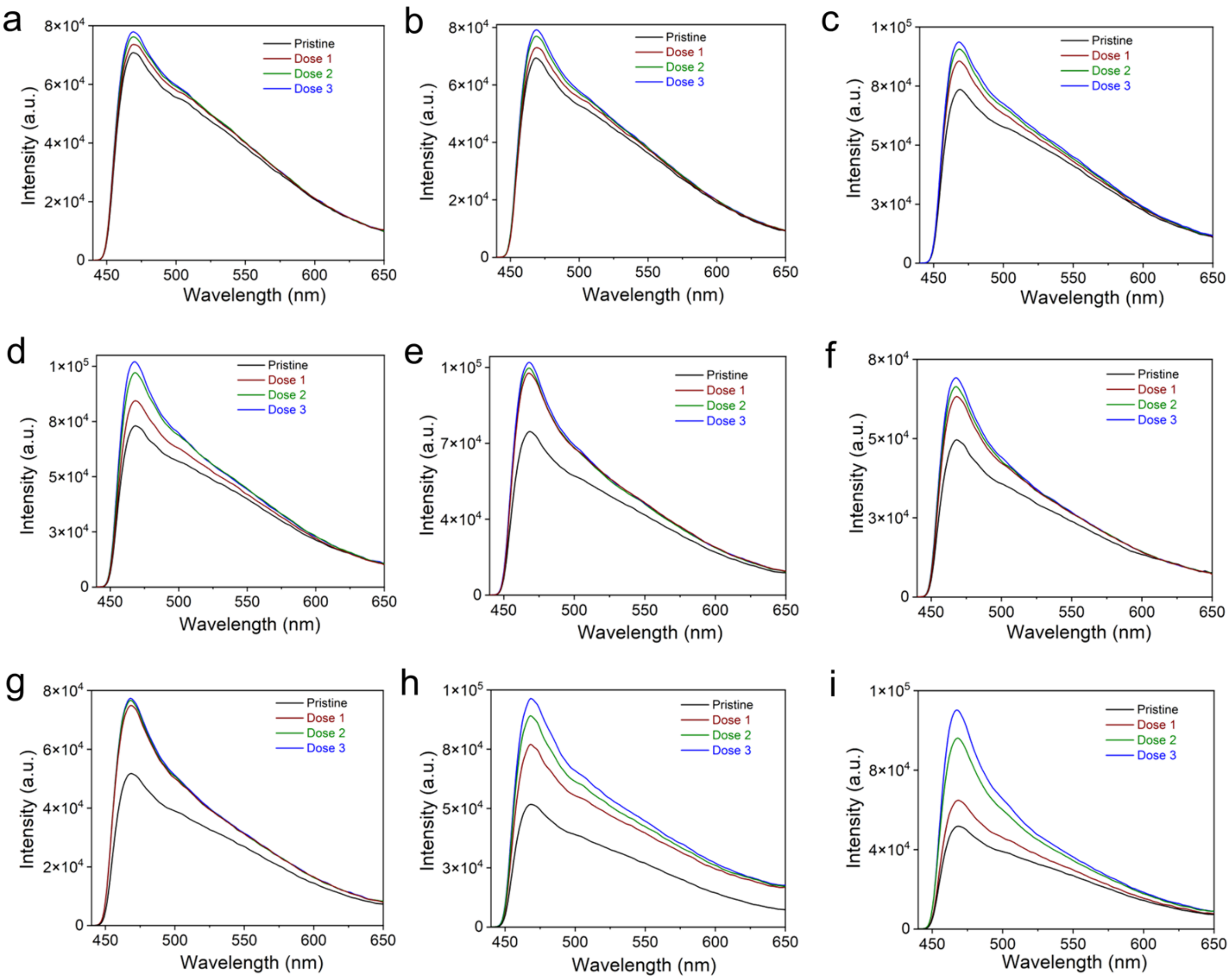


**Fig. S22.** Turn on sensing of different concentrations of acetone vapor by utilizing ZnQM(4) powder. **(a)** 1 ppm, **(b)** 2 ppm, **(c)** 3 ppm, **(d)** 4 ppm, **(e)** 6 ppm, **(f)** 8 ppm, **(g)** 12 ppm, **(h)** 20 ppm, and **(i)** 40 ppm.

**Table S10.** Acetone vapor selectivity of ZnQM(4) powder was examined in the presence of a mixture containing 100 ppm water vapor and 100 ppm methanol vapor.

| **Acetone Quantity in ppm** | **Intensity increment (Only Acetone)** | **Intensity increment (100 ppm water + 100 ppm Methanol + varying quantities of Acetone) (Mixture of three components)** |
|---|---|---|
| 1 ppm | ~ 11.1 % | ~ 10.7 % |
| 2 ppm | ~ 15.3 % | ~ 15.1 % |
| 3 ppm | ~23.6 % | ~23.6 % |
| 4 ppm | ~ 40.3 % | ~39.8 % |
| 6 ppm | ~ 48.1 % | ~47.4 % |
| 8 ppm | ~ 54.7 % | ~ 54.3 % |
| 12 ppm | ~ 68.6 % | ~ 68.1 % |
| 20 ppm | ~ 86.2 % | ~ 86.4 % |
| 40 ppm | ~ 114.1 % | ~ 113.8 % |

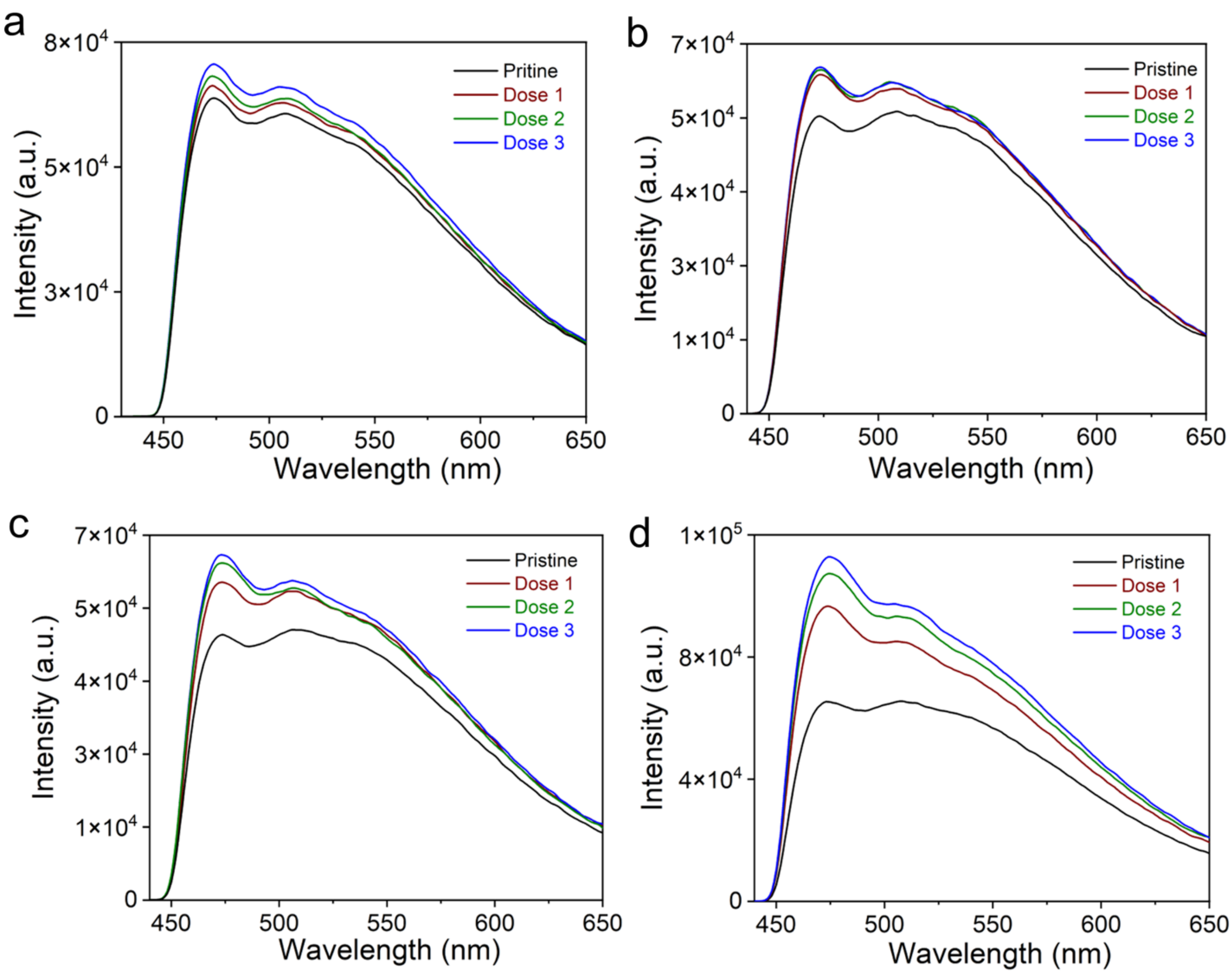


**Fig. S23.** Turn-on luminescent sensing of different concentrations of acetone vapor by utilizing InQM(4) powder. **(a)** 0.6 ppm, **(b)** 1 ppm, **(c)** 2 ppm, and **(d)** 4 ppm.

**Table S11.** Acetone vapor selectivity of InQM(4) powder was examined in the presence of a mixture containing 100 ppm water vapor and 100 ppm methanol vapor.

| **Acetone Quantity** | **Intensity increment (100 ppm water + 100 ppm Methanol + varying quantities of Acetone) (Mixture of three components)** |
|---|---|
| 0.6 ppm | ~ 10.4 % |
| 1 ppm | ~ 16.8 % |
| 2 ppm | ~ 30.1 % |
| 4 ppm | ~ 71.7 % |

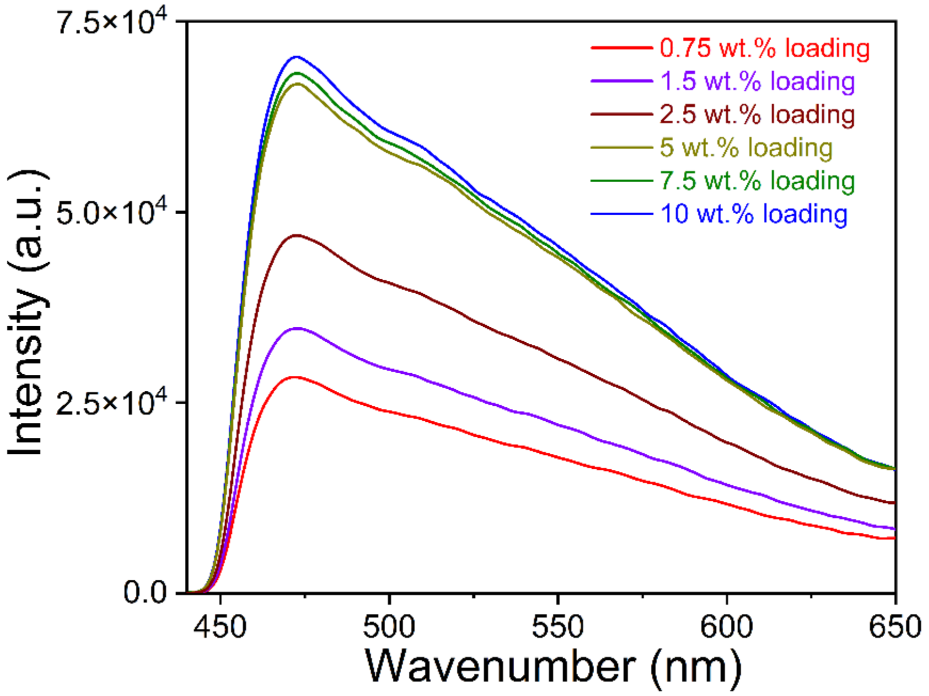


**Fig. S24.** Emission spectra of PVDF film containing different loadings of GaQM(4) as a filler in PVDF polymer matrices. The emission spectra exhibit that the emission intensity of the spectra experiences minimal increase beyond a filler loading of 5 wt.%.

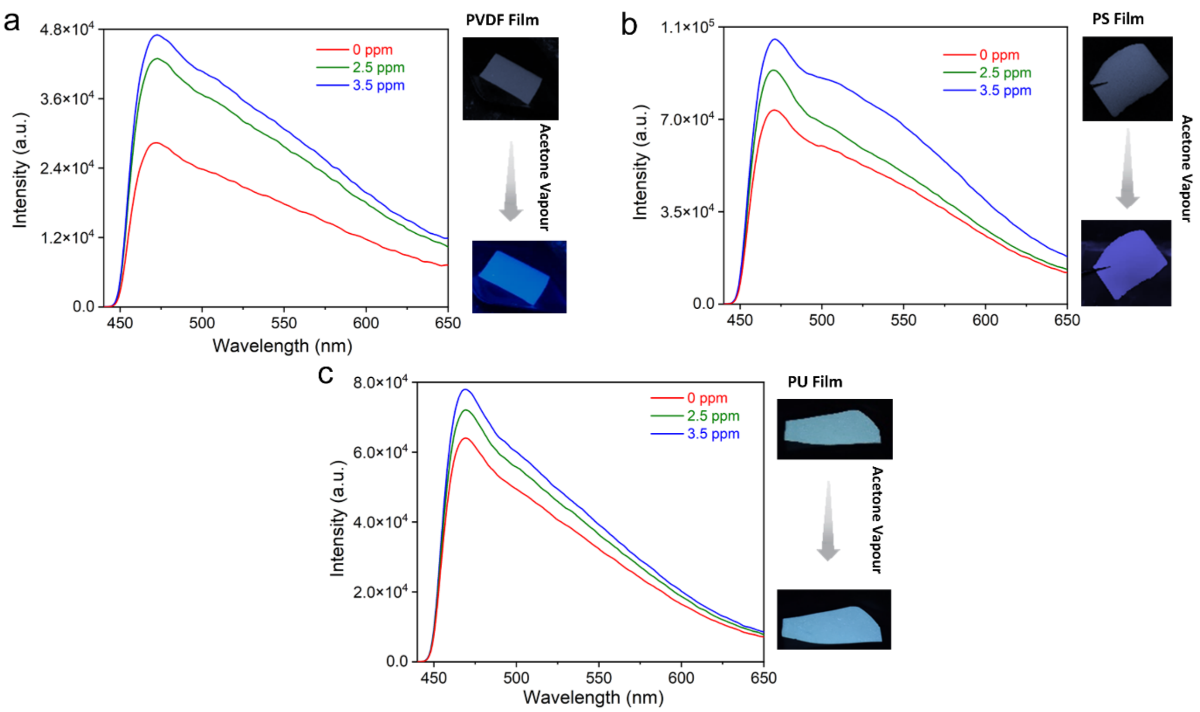


**Fig. S25.** Film acetone vapor sensing in two different concentrations of 2.5 ppm and 3.5 ppm. All the films are fabricated by blending grounded GaQM(4) powder and different polymer matrices where the filler loading was 5 wt.%. **(a)** PVDF, **(b)** PS and **(c)** PU.

**Table S12.** Acetone vapor selectivity of different luminescent films. All the films contain 2.5 wt.% of GaQM(4) as filler loading and all films have a thickness of approximately 160 microns.

| **Different Films** | **Intensity increment** (2.5 ppm Acetone) | **Intensity increment** (3.5 ppm Acetone) |
|---|---|---|
| PU | 12.5 ± 1.6 % | 21.8 ± 2.8 % |
| PS | 20.1 ± 1.1 % | 36.5 ± 1.7 % |
| PVDF | 50.7 ± 2.7 % | 72.8 ± 4.4 % |

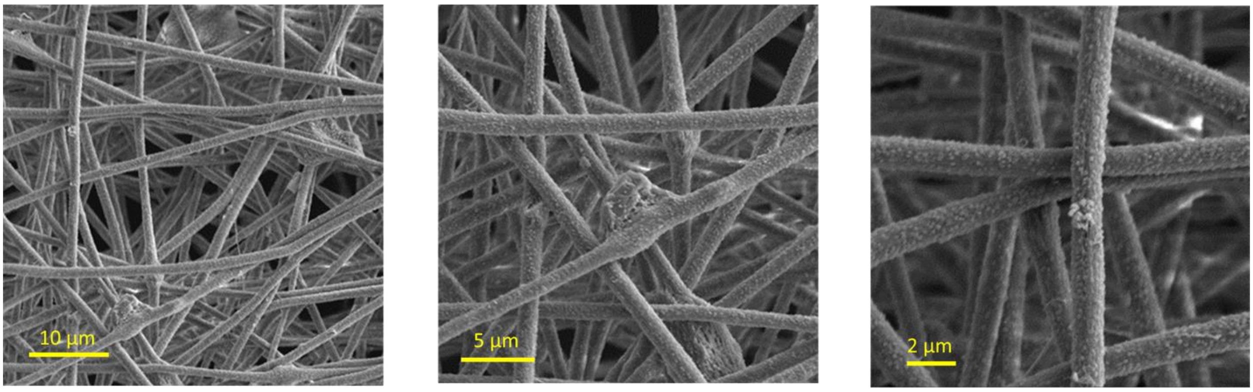


**Fig. S26.** FESEM micrographs of pristine PVDF fiber.

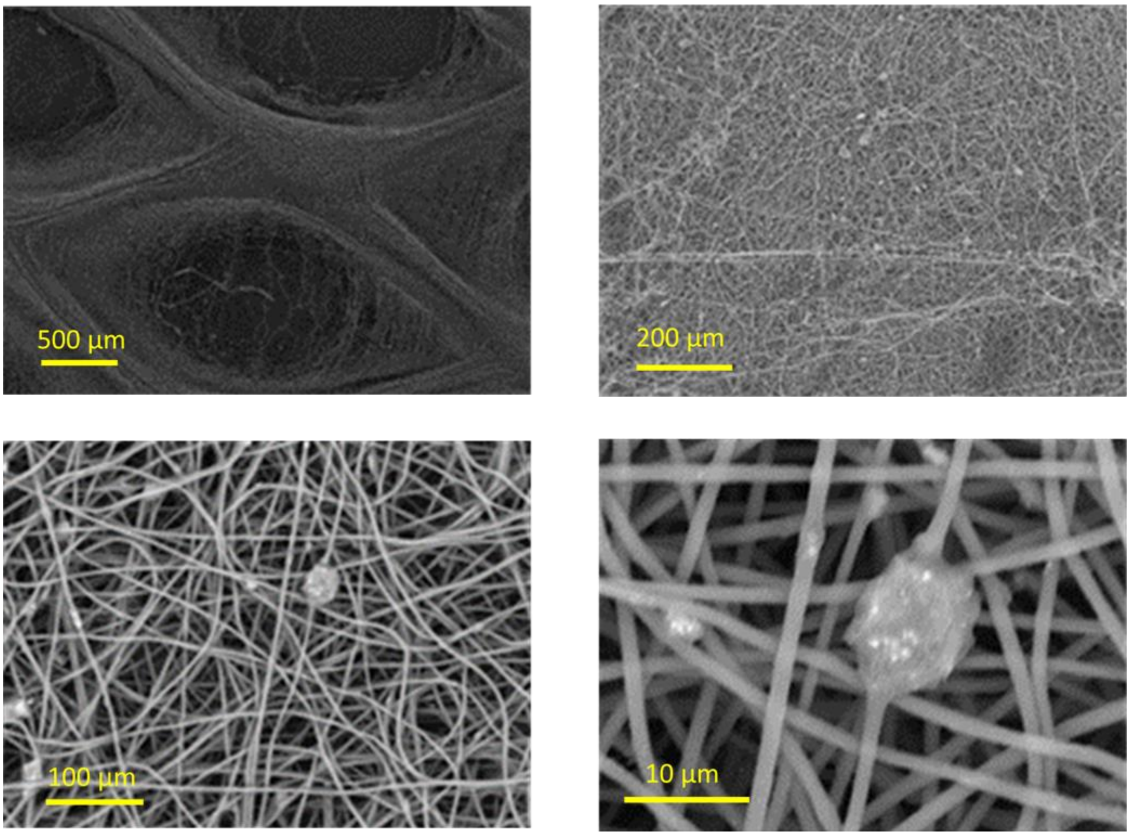


**Fig. S27.** FESEM micrographs of GaQM(4)/PVDF fibers.

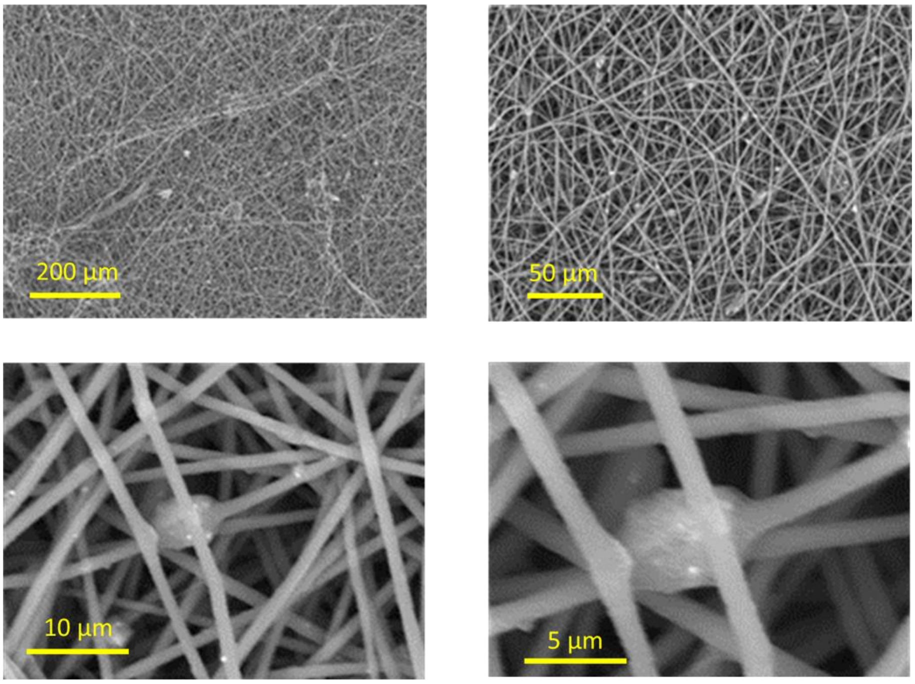


**Fig. S28.** FESEM micrographs of ZnQM(4)/PVDF fibers.

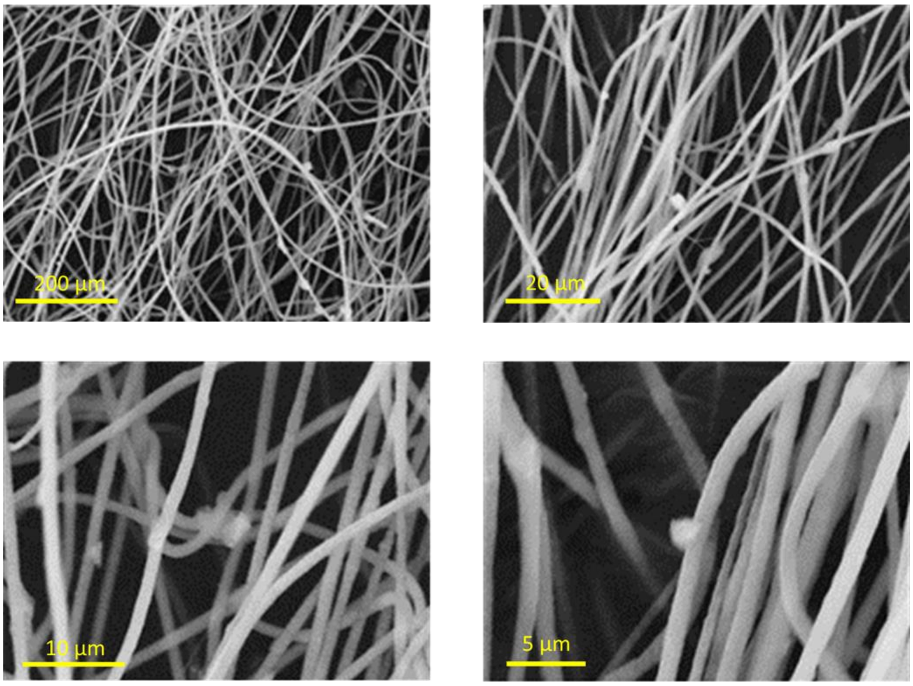


**Fig. S29.** SEM micrographs of InQM(4)/PVDF fibers.

**Table S13.** Acetone vapor sensing by luminescent GaQM(4)/PVDF fiber.

| **Acetone Concentrations (ppb)** | **Intensity increment** |
|---|---|
| 1500 | 22.3 ± 1.9 % |
| 2000 | 36.1 ± 2.3 % |
| 2500 | 48.2 ± 2.8 % |
| 3000 | 59.6 ± 3.4 % |
| 3500 | 71.4 ± 4.2 % |

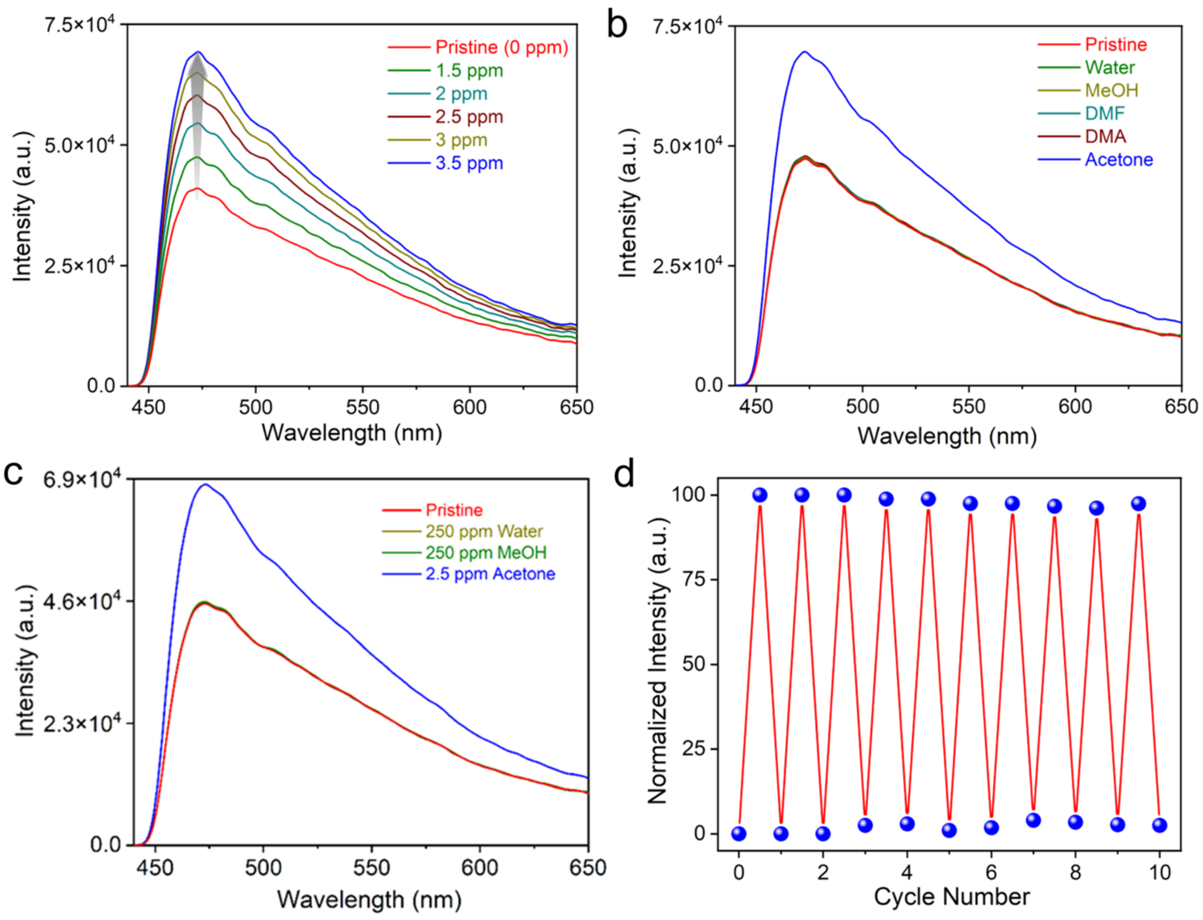


**Fig. S30.** Acetone vapor sensing by GaQM(4)/PVDF Fiber where filler GaQ(4) is fixed to 5 wt.%. **(a)** Turn-on emission spectra of GaQM(4)/PVDF fiber in the presence of various acetone vapor concentrations in sub-diabetes ranges. **(b)** Turn-on emission spectra of GaQM(4) for acetone selectivity testing in the presence of other VOCs. **(c)** Turn-on emission spectra of GaQM(4)/GMF composite for acetone selectivity test in presence of large excess (100 times larger) of water vapor and methanol vapor. **(d)** Turn-on emission spectra of GaQM(4)/PVDF fiber for acetone selectivity test in the presence of mixtures of other VOCs.

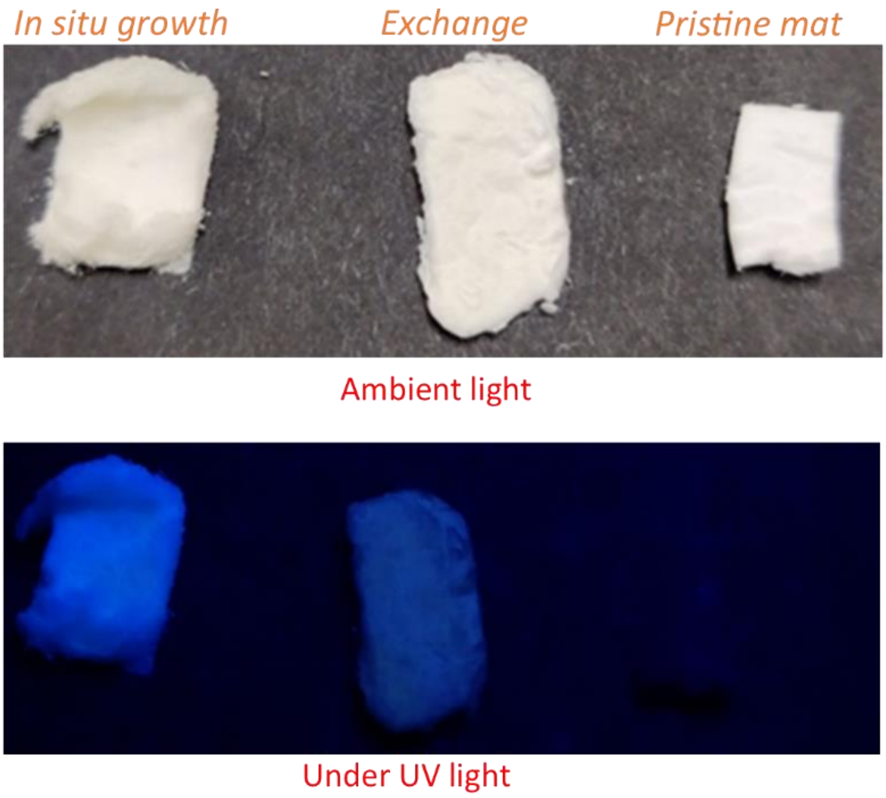


**Fig. S31.** Photos of the GaQM(4)/GMF composite under ambient light and under UV light (365 nm). The *in situ* growth refers to the incorporation of GaQM(4) through solvothermal synthesis into the pristine mat of GMF filter, while the exchange product refers to the GaQM(4) materials that are introduced into the pristine GMF filter through post-synthetic exchange.

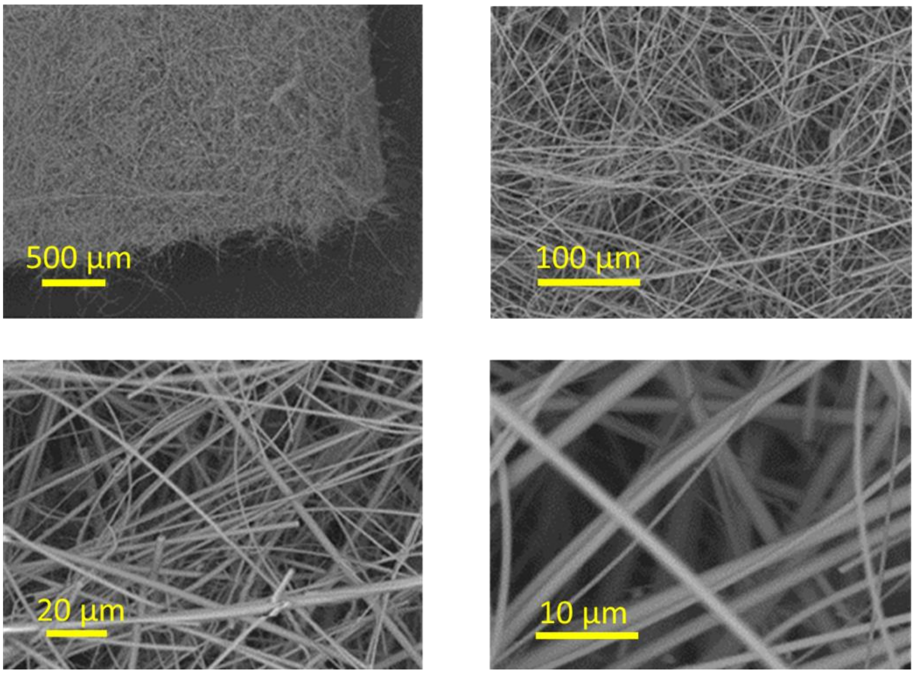


**Fig. S32.** SEM micrographs of pristine mat of glass microfiber (GMF).

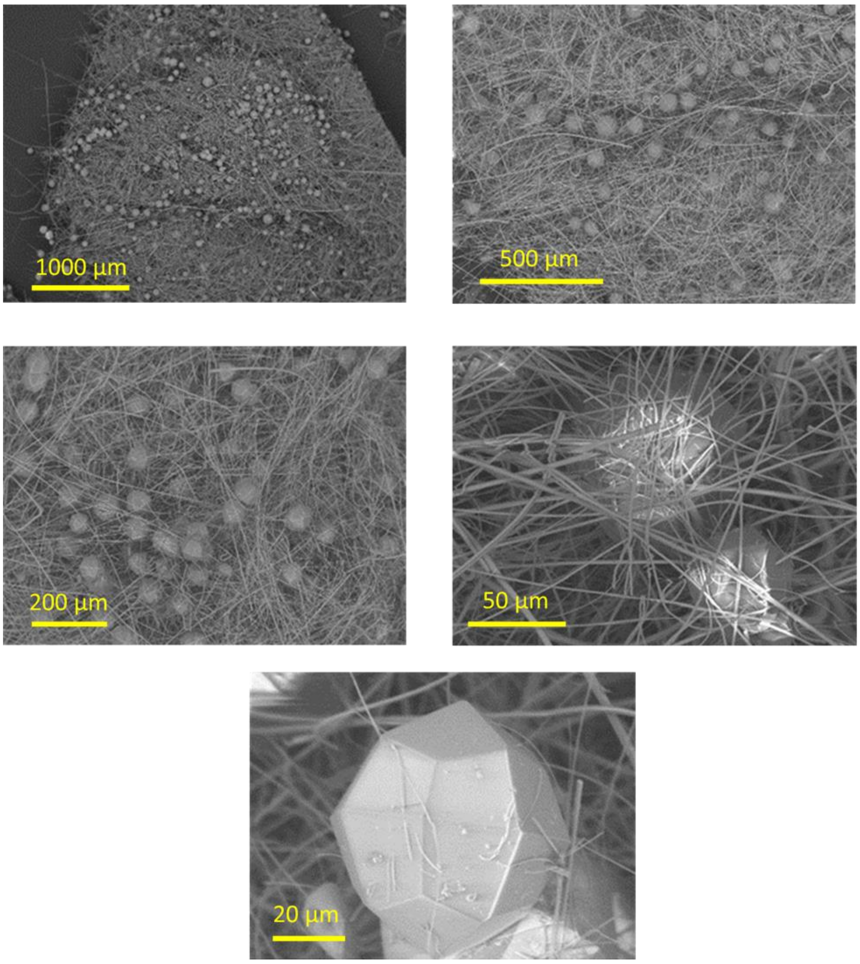


**Fig. S33.** SEM micrographs of GaQM(4)/GMF composite.

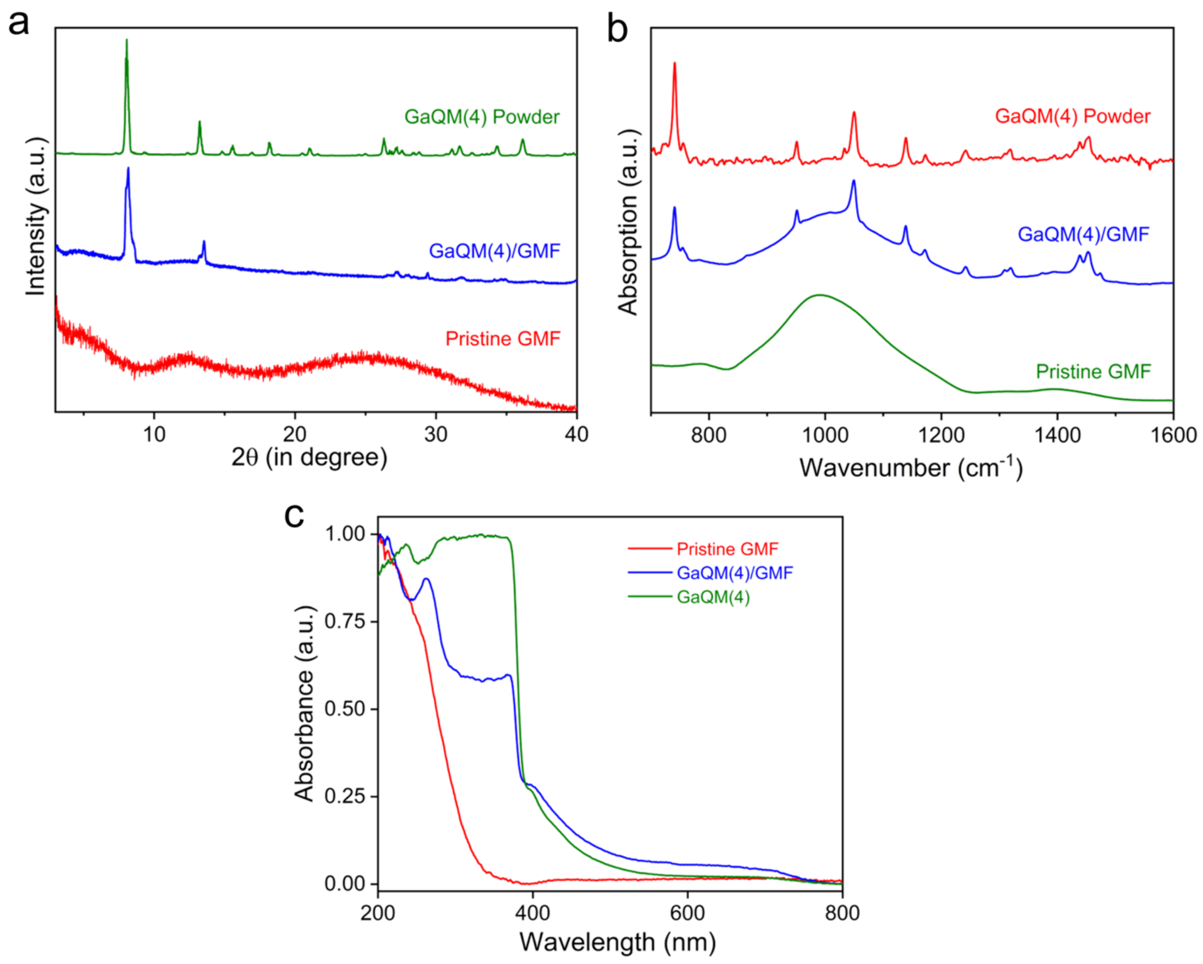


**Fig. S34.** Characterizations of GaQM(4)/GMF composite. **(a)** PXRD, **(b)** FTIR, **(c)** UV-Vis diffused reflectance spectroscopy. The presence of characteristic peaks for GaQM(4) in the GMF fiber from all the experiments confirms the successful embedment of GaQ(M) single crystals in the GMF mat.

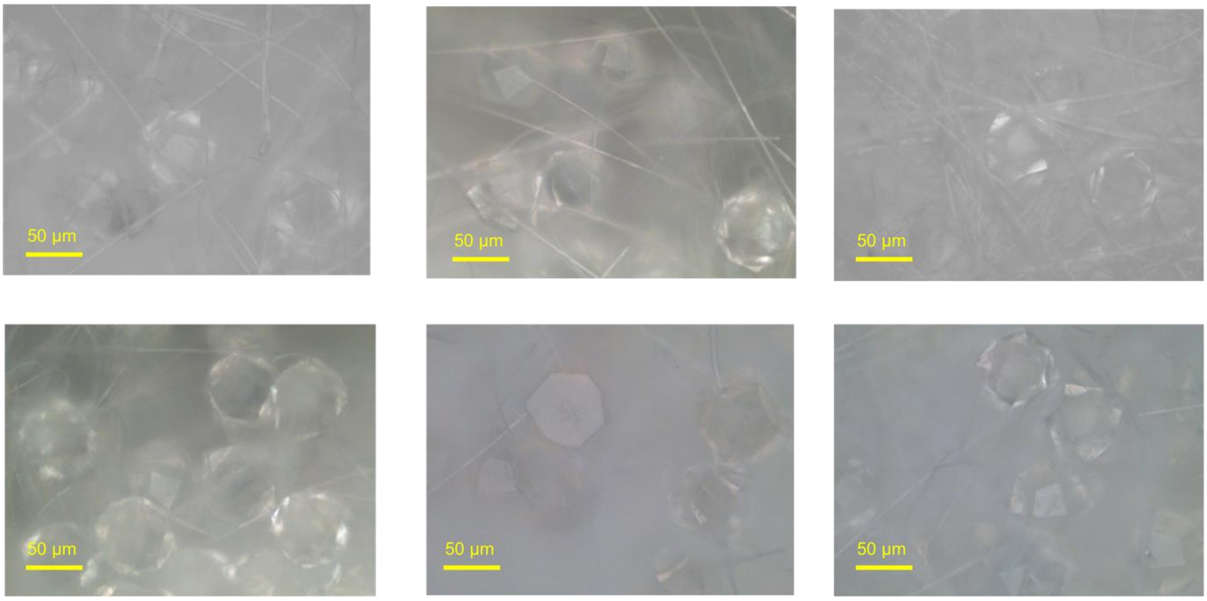


**Fig. S35.** Confocal Raman images of GaQM(4)/GMF composites in different regions.

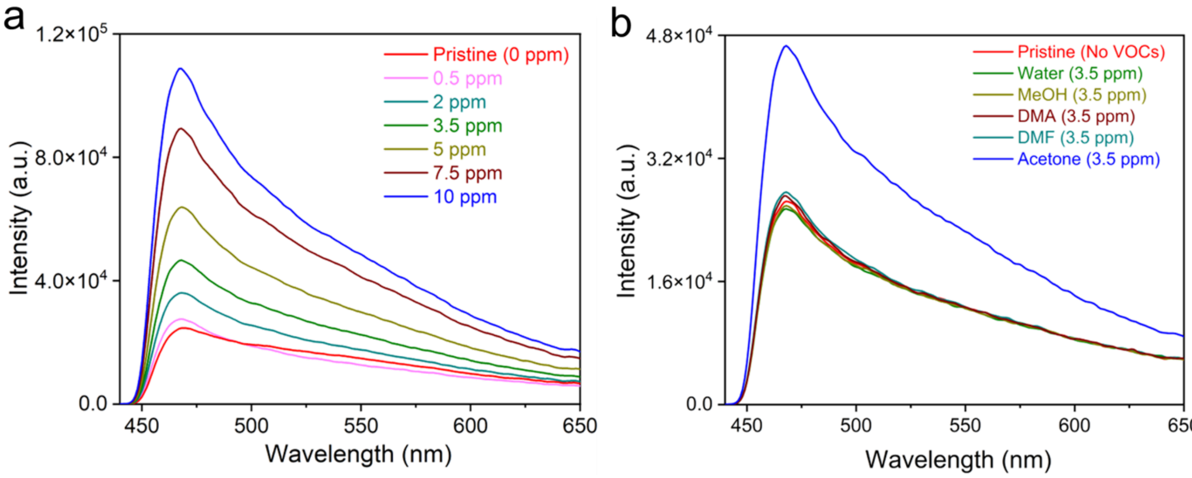


**Fig. S36. (a)** Turn-on emission spectra of GaQM(4)/GMF composite in the presence of various acetone vapor concentrations. **(b)** Turn-on emission spectra of GaQM(4)/GMF composite for acetone selectivity testing in the presence of a mixture of an equal concentrations (3.5 ppm each) of other VOCs. Here water, methanol, DMF, DMA and hexane were used 3.5 ppm concentration of each in the VOC mixtures.

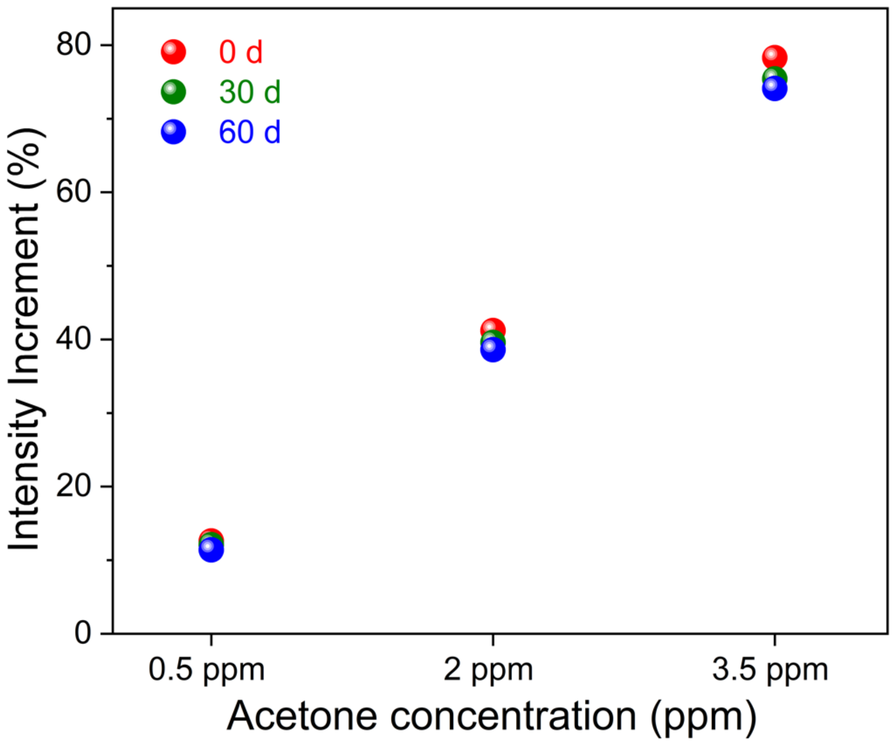


**Fig. S37.** Acetone vapor sensing by GaQM(4)/GMF composite within the sub-diabetic range subsequent to immersion in water for several days.

**Table S14.** Complete assignment of the vibrational modes of MAF-5.

Complete assignment of all the vibrational modes of MAF-5 between 0-190 $cm^{-1}$ <5.7 THz), 171-600 $cm^{-1}$ (~5.7-18 THz), 601-1200 $cm^{-1}$ (~18-36 THz), and 1201-4000 $cm^{-1}$ (~36-120 THz) based on the DFT simulations. Stretching: υ; Bending: δ; Scissoring: $\delta_s$ ; Rocking: ρ; wagging: ω; Twisting: T

Collective THz modes, 0-190 $cm^{-1}$

| ***Frequency*** | ***Intensity (a.u.)*** | ***Assignment*** |
|---|---|---|
| 25.85 | 2 | Et ρ, pore breathing, pore shearing |
| 87.85 | 1 | 4MR, 6 MR breathing, pore opening, shearing/ 4MR pore deformation |
| 94.25 | 8 | 4MR shearing, ($Zn-N_4$) distortion, 6 MR gate opening |
| 98.58 | 25 | Lattice vibration, (Zn-N) ρ, ($Zn-N_4$) deformation |
| 124.59 | 94 | (Zn-N) $\delta_s$, (Zn-N) v, (N-C-N) $v_s$ |
| 132.20 | 158 | (Zn-N) $v_s$, (Zn-N) $\delta_s$, lattice vibration |
| 138.64 | 49 | ($Zn-N_4$) distortion, $v_s$, $(C-C)_{Et}$ ρ, 6MR deformation, 4 MR breathing |
| 143.60 | 6 | Lattice vibration, 4MR distortion, 6MR shearing, Im ring $v_s$ |
| 147.94 | 31 | Collective vibration of framework, pore breathing |
| 181.32 | 1 | Im ring twisting, ($Zn-N_4$) distortion, $(C-C)_{Et}$ T |
| 187.74 | 540 | ($Zn-N_4$) deformation, (C-N-C) $v_s$, (Zn-N) $v_s$, 6MR, 4MR deformation, Im deformation |
| 190.16 | 4 | ($Zn-N_4$) deformation, Im ring deformation, 6MR aperture expansion |

Vibrational modes 191-600 $cm^{-1}$

| | | |
|---|---|---|
| 207.30 | 71 | ($Zn-N_4$) deformation, $(C-C)_{Et}$ ρ, 6MR expansion |
| 211.52 | 386 | (Zn-N) T, 6 MR gate opening |
| 214.13 | 161 | 4,8 MR shearing, ($Zn-N_4$) distortion, Im $v_s$ expansion |
| 229.25 | 25 | (Zn-N) distortion, (C-N-C) $v_s$ |
| 232.49 | 30 | ($Zn-N_4$) distortion, (Zn-N) T, (Zn-N) ω, (N-C-N) $v_s$ |
| 233.47 | 38 | (Zn-N) $v_s$, $(C-C)_{Et}$ ρ, |

| | | |
|---|---|---|
| 263.81 | 2 | (Zn-N) $\delta_s$, (Zn-N) $v_s$, (Et-C) $v_s$ |
| 267.18 | 233 | (Zn-N) $v_s$, $(C-C)_{Et}$ T, $(N-C-C)_{Im}$ $v_s$ |
| 269.01 | 14 | $(C-C)_{Et}$ T |
| 275.59 | 1928 | (Zn-N) $v_a$, 6MR deformation, (Zn-N) $\rho$, (C-N-C) $v_a$, $(C-C)_{Et}$ T |
| 286.56 | 603 | (Zn-N) $v_s$, (C-N-C) $v_s$, $(Zn-N_4)$ distortion, |
| 229.25 | 25 | (Zn-N) distortion, (C-N-C) $v_s$ |
| 232.49 | 30 | $(Zn-N_4)$ distortion, (Zn-N) T, (Zn-N) $\omega$, (N-C-N) $v_s$ |
| 233.47 | 38 | (Zn-N) $v_s$, $(C-C)_{Et}$ $\rho$, |
| 263.81 | 2 | (Zn-N) $\delta_s$, (Zn-N) $v_s$, (Et-C) $v_s$ |
| 267.18 | 233 | (Zn-N) $v_s$, $(C-C)_{Et}$ T, $(N-C-C)_{Im}$ $v_s$ |
| 269.01 | 14 | $(C-C)_{Et}$ T |
| 275.59 | 1928 | (Zn-N) $v_a$, 6MR deformation, (Zn-N) $\rho$, (C-N-C) $v_a$, $(C-C)_{Et}$ T |
| 286.56 | 603 | (Zn-N) $v_s$, (C-N-C) $v_s$, $Zn-N_4$ distortion, |
| 295.11 | 795 | (Zn-N) $v_s$, (C-Et) $v_s$, (C-N-C) $v_s$ |
| 404.59 | 1317 | Im ring deformation, (C-Et) v, (Zn-N) $v_a$, $(C-C)_{Et}$ $v_a$ |
| 418.69 | 149 | Im ring deformation |
| 444.05 | 669 | (Zn-N) $\rho$, $(C-C)_{Et}$ $v_s$, (N-C-N) $v_s$ |
| 446.81 | 2108 | Zn-n $v_s$, Im ring distortion, $(C-C)_{Et}$ T, 6 MR opening |

Vibrational modes 600-1200 $cm^{-1}$ (Ring deformations)

| | | |
|---|---|---|
| 671.62 | 130 | Out of plane deformation of imidazole ring |
| 673.89 | 1 | In plane deformation of imidazole ring |
| 674.30 | 165 | Out of plane deformation of imidazole ring |
| 674.90 | 164 | Out of plane deformation of imidazole ring |

| | | |
|---|---|---|
| 682.42 | 1 | Out of plane deformation of imidazole ring |
| 769.34 | 3719 | Out of plane deformation of imidazole ring, $(C\text{-}H)_{Im}$ ω, $(C\text{-}H)_{Et}$ ω |
| 776.13 | 87 | Out of plane deformation of imidazole ring, $(C\text{-}H)_{Im}$ ω |
| 784.21 | 77 | Out of plane deformation of imidazole ring, (C-Et) ρ |
| 786.76 | 251 | Out of plane deformation of imidazole ring |
| 790.39 | 6 | In plane deformation of imidazole ring, $(C\text{-}H)_{Im}$ ω |
| 805.04 | 67 | In plane deformation of imidazole ring, $(C\text{-}H)_{Im}$ ω, $(C\text{-}C)_{Et}$ T |
| 820.7 | 87 | Out of plane deformation of imidazole ring, $(C\text{-}H)_{Im}$ ω |
| 823.51 | 206 | In plane deformation of imidazole ring |
| 823.98 | 62 | Out of plane deformation of imidazole ring, $(C\text{-}H)_{Im}$ ω, $(C\text{-}H)_{Et}$ ρ |
| 860.79 | 14 | Out of plane deformation of imidazole ring, $(C\text{-}H)_{Im}$ ω |
| 875.08 | 119 | In plane deformation of imidazole ring, $(C\text{-}H)_{Im}$ ω, $(C\text{-}C)_{Et}$ T |
| 912.90 | 114 | Out of plane deformation of imidazole ring, $(C\text{-}H)_{Im}$ ω |
| 972.03 | 380 | In plane deformation of imidazole ring |
| 972.95 | 713 | In plane deformation of imidazole ring |
| 977.08 | 87 | In plane deformation of imidazole ring |
| 1003.42 | 1 | In plane deformation of imidazole ring |
| 1003.95 | 1 | In plane deformation of imidazole ring |
| 1005.79 | 293 | In plane deformation of imidazole ring |
| 1067.20 | 1 | In plane deformation of imidazole ring, (C-H) ρ |
| 1067.84 | 1 | In plane deformation of imidazole ring, (C-H) ρ |
| 1071.31 | 8 | In plane deformation of imidazole ring (small), $(C\text{-}H)_{Et}$ ρ |
| 1092.62 | 1246 | In plane deformation of imidazole ring, $(C\text{-}H)_{Et}$ ρ, $(C\text{-}C)_{Et}$ T |

| | | |
|---|---|---|
| 1097.24 | 3349 | In plane deformation of imidazole ring, $(C\text{-}H)_{Et}$ ρ |
| 1098.71 | 266 | In plane deformation of imidazole ring, $(C\text{-}C)_{Et}$ $v_s$ |
| 1114.82 | 49 | In plane deformation of imidazole ring, $(C\text{-}C)_{Et}$ $v_a$ |
| 1115.16 | 234 | In plane deformation of imidazole ring, $(C\text{-}C)_{Et}$ $v_a$, (C-C-N) $v_a$ |
| 1123.24 | 121 | $(C\text{-}H)_{Et}$ ρ |
| 1183.79 | 87 | In plane deformation of imidazole ring, $(C\text{-}H)_{Im}$ $\delta_s$ |
| 1184.86 | 592 | In plane deformation of imidazole ring, $(C\text{-}H)_{Im}$ $\delta_s$ |
| 1190.99 | 519 | In plane deformation of imidazole ring, $(C\text{-}H)_{Im}$ $\delta_s$ |
| 1193.25 | 1287 | In plane deformation of imidazole ring, $(C\text{-}H)_{Im}$ $\delta_s$ |
| 1199.62 | 1234 | In plane deformation of imidazole ring, $(C\text{-}H)_{Im}$ ρ |

Vibrational modes 1200-4000 $cm^{-1}$

| | | |
|---|---|---|
| 1246.64 | 525 | CN v, (Zn-N-C) v |
| 1249.80 | 153 | CN v, (C-N-C) v |
| 1250.75 | 5 | (C-N-C) v |
| 1286.76 | 17 | C-Et $v_a$, Et v |
| 1295.64 | 123 | (Et) v, $(CH)_{Et}$ ρ |
| 1297.63 | 110 | (Et) v, $(CH)_{Et}$ ρ |
| 1361.54 | 157 | (Zn-N-C) v, (Et) v |
| 1363.38 | 34 | CN v, $(CH)_{Et}$ ρ |
| 1363.80 | 545 | (C-Et) $v_s$, $(CH)_{Et}$ v, (Zn-N-C) v |
| 1396.39 | 18 | CN v, (C-Et) v |
| 1397.87 | 643 | CN v, CN $v_s$, $(CH)_{Et}$ ρ |
| 1428.98 | 172 | $(CH)_{Et}$ ω, $(C\text{-}C)_{Et}$ T |
| 1429.26 | 5 | $(C\text{-}CH_3)_{Et}$ v, $CH_3$ ω, (Zn-N) v (small) |

| | | |
|---|---|---|
| 1443.74 | 18 | $CH_3$ $\delta_s$, $(CH)_{CH3}$ v, (C-Et) v |
| 1496.16 | 1 | (N-C-N) v, $(CH_3)_{Et}$ $\delta_s$ |
| 1496.85 | 337 | (N-C-N) $v_s$, $(CH_3)_{Et}$ $\delta_s$ |
| 1503.22 | 131 | N-C-N vs, $(CH_2)_{Et}$ $\delta_s$, $(CH)_{Im}$ vs |
| 1507.77 | 1378 | (N-C-N) v, $(CH_2)$ $\delta_s$ |
| 1508.32 | 299 | Et v |
| 1510.34 | 300 | $(CH)_{Et}$ v |
| 1510.79 | 6 | $(CH)_{Et}$ v |
| 1512.27 | 173 | $(CH)_{Et}$ v |
| 1519.91 | 5978 | $(CH)_{Im}$ v, $(CH)_{Et}$ v |
| 1521.35 | 1661 | (N-C-N) v, $(CH)_{Et}$ v, $(CH)_{Im}$ v |
| 1539.18 | 361 | $(CH)_{Et}$ v |
| 1540.72 | 81 | In plane deformation of imidazole ring, CN v, $(C\text{-}H)_{Et}$ v, |
| 1543.33 | 645 | (N-C-N) v, $(C\text{-}C)_{Et}$ v |
| 1586.55 | 3 | $(C\text{-}C)_{Im}$ $v_s$ |
| 1587.82 | 10 | $(C\text{-}C)_{Im}$ v, (N-C-N) v |
| 1591.22 | 336 | $(C\text{-}C)_{Im}$ v, (N-C-N) v |
| 3058.20 | 617 | $(CH_2)_{Et}$ $v_s$ |

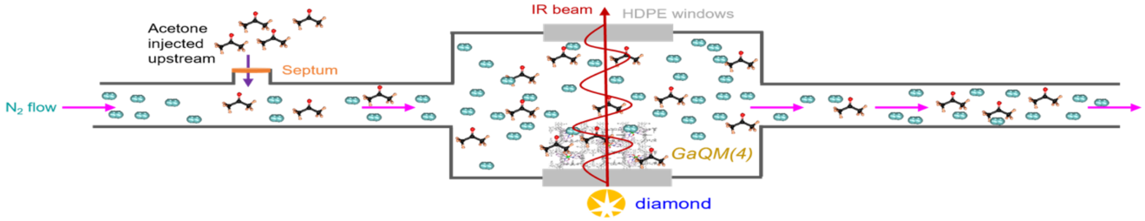


**Fig. S38.** Schematic of the synchrotron based FTIR gas dosing experiments.

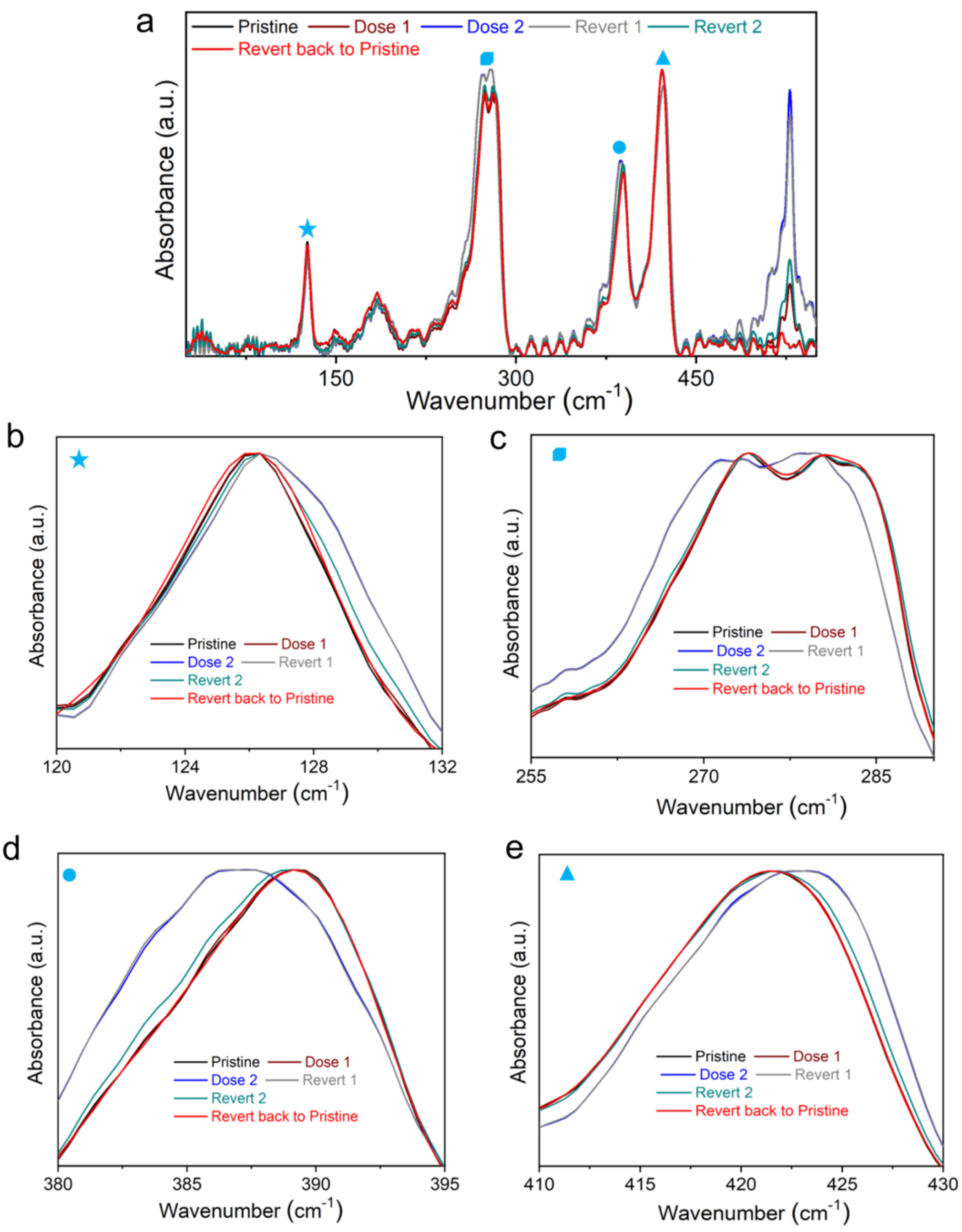


**Fig. S39.** SR-FTIR dosing spectra of GaQM(4) composite powder when subjected to 50 µL of acetone vapor. Dose 1 and Dose 2 are the exposure of the of acetone gas while revert 1 and 2 are the removal of the acetone vapor from the sample. **(a)** The signature bands are highlighted in the full range of spectrum in farIR region. Signature vibrational bands at **(b)** 125 $cm^{-1}$, **(c)** 278 $cm^{-1}$, **(d)** 389 $cm^{-1}$ and **(e)** 421 $cm^{-1}$ are indicative of the signature vibrations. The SR-FTIR demonstrates all the signature vibrational bands are shifted presence of acetone vapor demonstrates the strong interaction between acetone molecules with the framework.

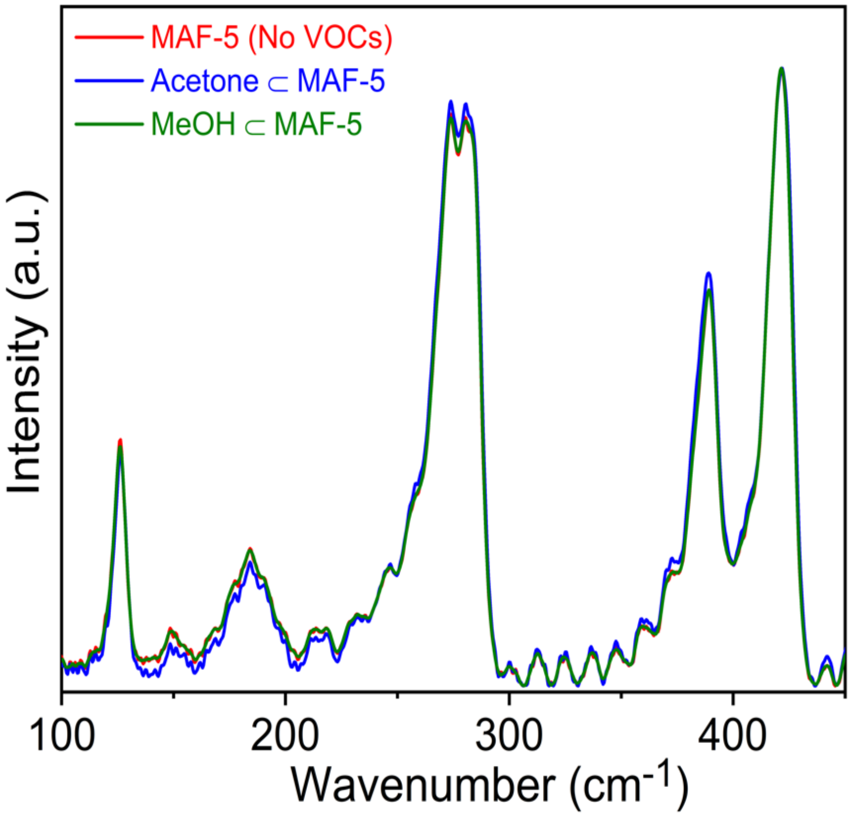


**Fig. S40.** SR-FTIR spectra of pristine MAF-5 powder after exposure to 50 μL of each acetone and methanol. The absence of shifts of any signature peaks of MAF-5 in the farIR region supported the lack of any supramolecular interactions between these VOCs with the framework.

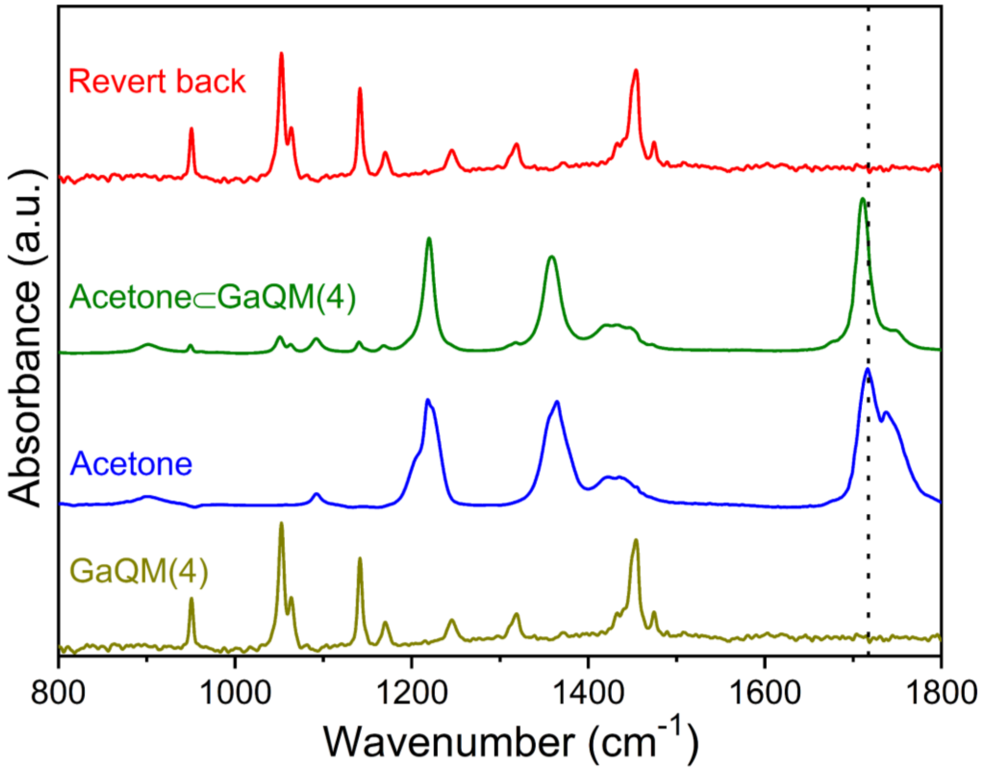


**Fig. S41.** FTIR spectra of the GaQM(4) composite powder demonstrating reversible interaction with acetone. The characteristic of acetone peak is highlighted with dotted line. The shifting of the signature band of carbonyl group indicates the interaction between acetone and the composite.

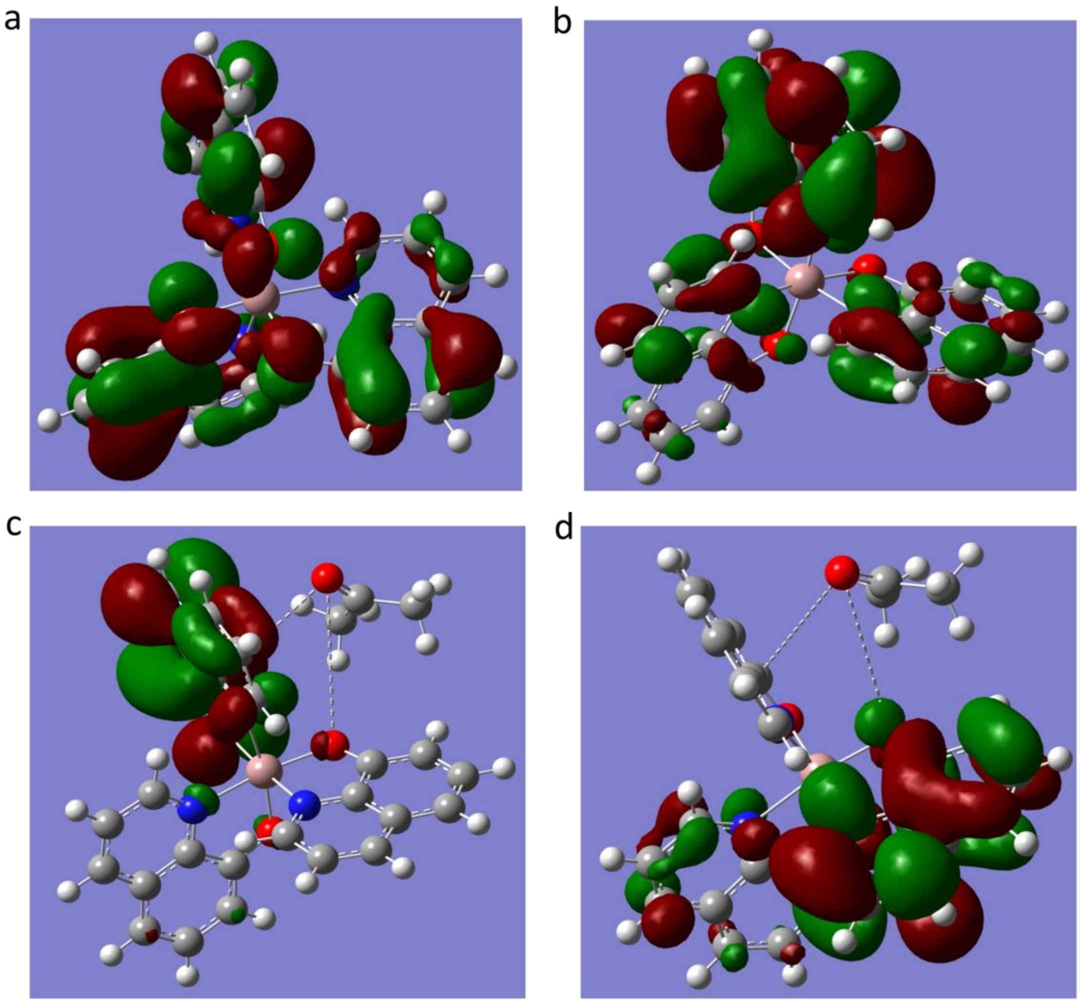


**Fig. S42.** Frontier molecular orbital images of highest occupied molecular orbital (HOMO) and lowest unoccupied molecular orbital (LUMO) of the bare GaQ complex and the Acetone⊂GaQ system. **(a)** HOMO of GaQ, **(b)** LUMO of GaQ, **(c)** HOMO of Acetone⊂GaQ and **(d)** LUMO of Acetone⊂GaQ. The frontier orbitals are distributed across HOMO and LUMO of bare GaQ quionolate linkers. In contrast, in the presence of acetone molecule (Acetone⊂GaQ), the frontier orbitals concentrated solely on HOMO of a single hydroxyquinolate linker. The acetone molecules also interact with the carbon atom of hydroxyquinolate linker where HOMO is concentrated. HOMO and LUMO calculated by using B3LYP/LANL2DZ basis set as implemented on Gaussian09.

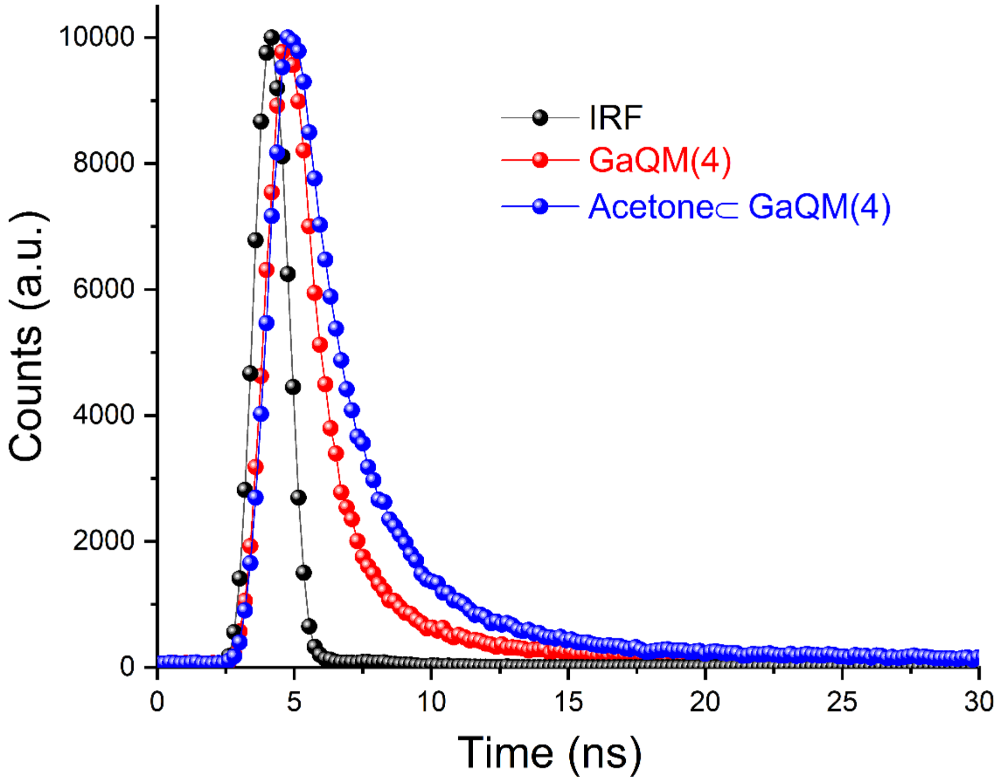


**Fig. S43.** Lifetime data of GaQM(4) powder and Acetone⊂GaQM(4) powder samples observed under 468 nm emission wavelength. IRF = instrument response function.

**Table S15.** PL decay parameters of before and during acetone exposed phase.

| **Compounds** | **$\tau_1$ (ns)** | **$A_1$** | **$\tau_2$ (ns)** | **$A_2$** | **$\tau_3$ (ns)** | **$A_3$** | **$\chi^2$** | **$\tau_{avg}$ (ns)** |
|---|---|---|---|---|---|---|---|---|
| GaQM(4) | 1.00 | 68.44 | 3.48 | 20.33 | 22.34 | 11.2 | 1.07 | 56.9 |
| Acetone⊂GaQM(4) | 1.65 | 60.87 | 4.22 | 2.99 | 24.19 | 13.14 | 1.08 | 63.5 |

**Table S16.** Calculations of radiative and nonradiative decay parameters. The radiative rate constant ($K_r$) and the nonradiative rate constant ($K_{nr}$) were estimated by using the following equations: PLQY = Kr × $\tau_{avg}$; $\tau_{avg} = 1/(K_r + K_{nr})$.

| **Compounds** | **PLQY** | **$\tau_{avg}$ (ns)** | **$K_r$ ($ns^{-1}$)** | **$K_{nr}$ ($ns^{-1}$)** | **$K_r/K_{nr}$** |
|---|---|---|---|---|---|
| GaQM(4) | 11.2 | 56.9 | 0.0019 | 0.0156 | 0.1218 |
| Acetone⊂GaQM(4) | 16.4 | 63.5 | 0.0026 | 0.0013 | 2 |

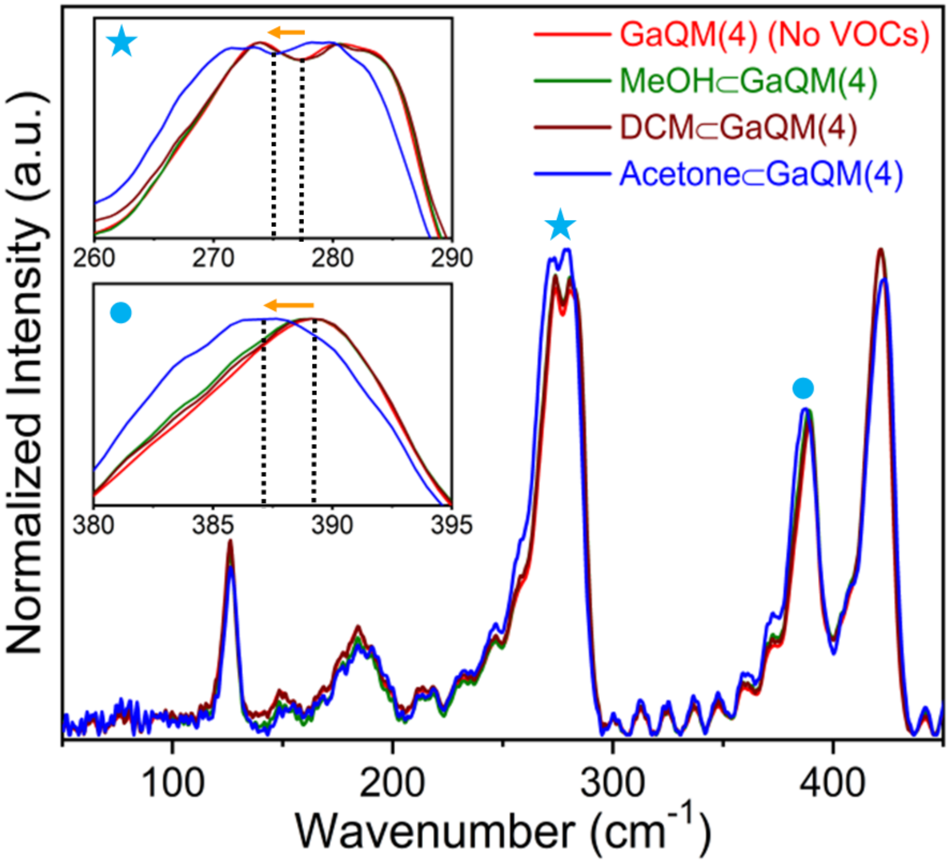


**Fig. S44.** Different gas dosing experiments with SR-FTIR of GaQM(4). Inset graphs show the red shifting of the highlighted parts of the figure, demonstrate acetone vapor selectivity in the presence of other VOCs where the concentration of each gas is kept fixed at 50 µL.

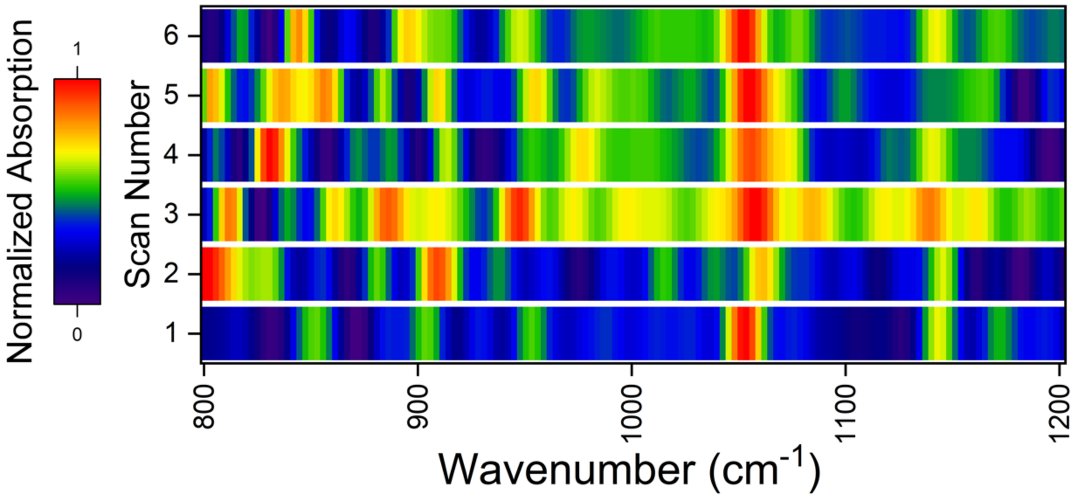


**Fig. S45.** NanoFTIR absorption spectra of a GaQM(4) crystals. The reversible displacement of this spectral band at 1053 $cm^{-1}$ indicates the acetone sensing behavior is reversible in nature at single crystal level.

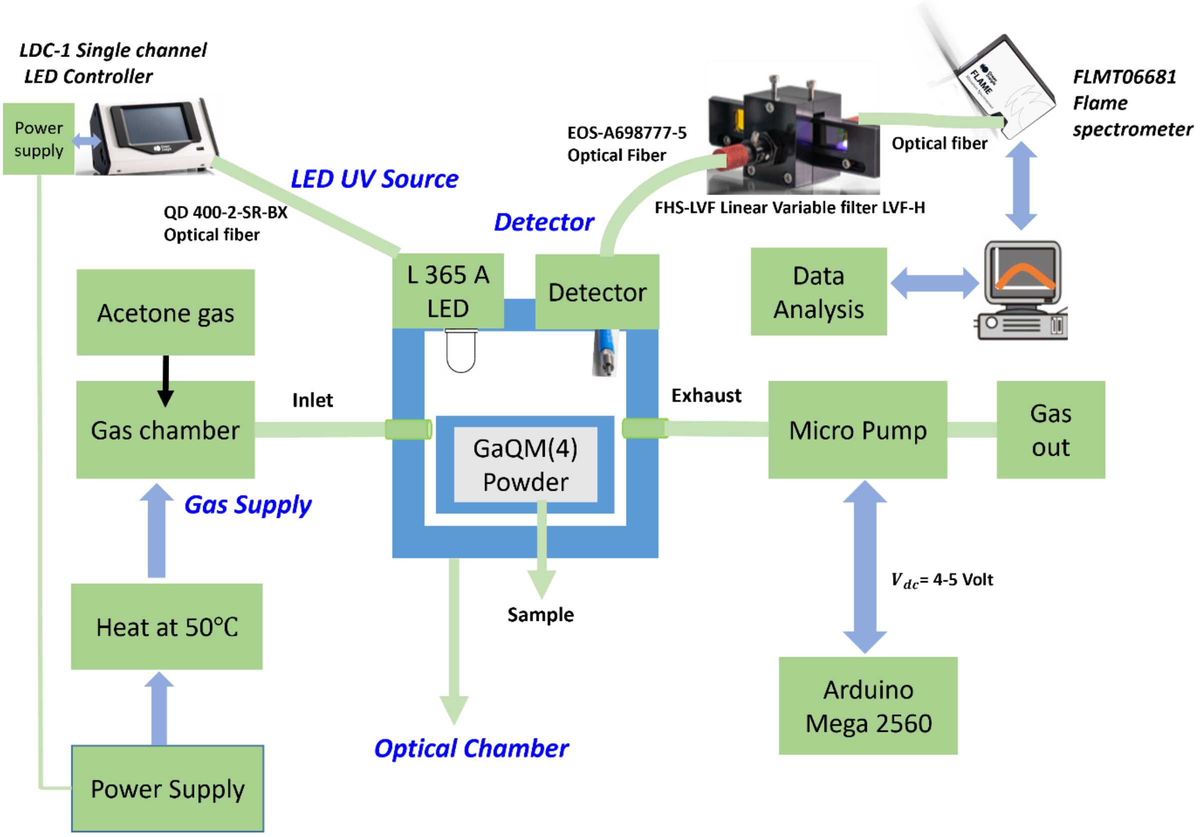


**Figure S46.** Scheme of the portable optical sensor device system for acetone detection by fluorescence approach.

**Table S17.** Acetone vapor sensing of GaQM(4) powder by portable optical sensor device.

| Acetone Concentrations (ppb) | Intensity increment |
|---|---|
| 1000 | 15.8 ± 1.7 % |
| 2000 | 26.03 ± 2.8 % |
| 3000 | 39.16 ± 3.4 % |
| 4000 | 53.63 ± 4.8 % |

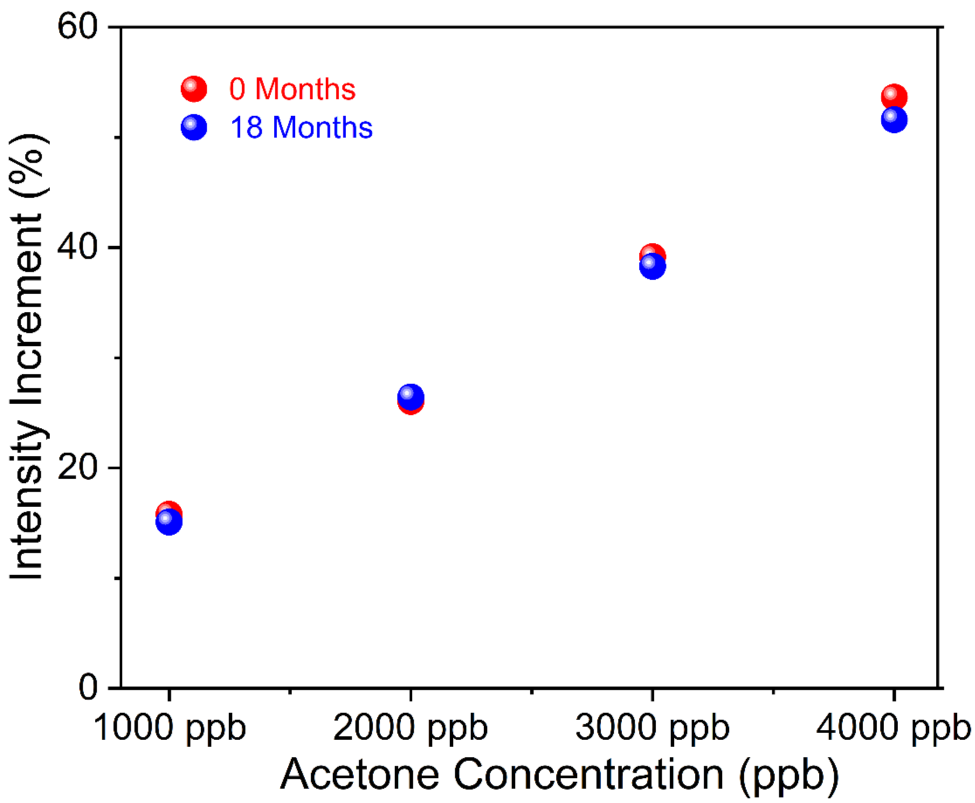


**Fig. S47.** Long-term stability of the portable optical sensor device for the detection of acetone vapor by GaQM(4) composite powder within the sub-diabetic range.

## References


[1] M. Basham, J. Filik, M. T. Wharmby, P. C. Y. Chang, B. El Kassaby, M. Gerring, J. Aishima, K. Levik, B. C. A. Pulford, I. Sikharulidze, D. Sneddon, M. Webber, S. S. Dhesi, F. Maccherozzi, O. Svensson, S. Brockhauser, G. Naray, A. W. Ashton, *J. Synchrotron Radiat.* **2015**, *22*, 853.

[2] J. Filik, A. W. Ashton, P. C. Y. Chang, P. A. Chater, S. J. Day, M. Drakopoulos, M. W. Gerring, M. L. Hart, O. V. Magdysyuk, S. Michalik, A. Smith, C. C. Tang, N. J. Terrill, M. T. Wharmby, H. Wilhelm, *J. Appl. Crystallogr.* **2017**, *50*, 959.

[3] A. A. Coelho, *J. Appl. Crystallogr.* **2018**, *51*, 210.

[4] S. Guo, H. Z. Li, Z. W. Wang, Z. Y. Zhu, S. H. Zhang, F. Wang, J. Zhang, *Inorg. Chem. Front.* **2022**, *9*, 2011.

[5] A. F. Moeslein, M. Gutierrez, K. Titov, L. Dona, B. Civalleri, M. D. Frogley, G. Cinque, S. Rudic, J. C. Tan, *Adv. Mater. Interfaces* **2022**, *10*, 2201401.

[6] L. Dona, J. G. Brandenburg, B. Civalleri, *J. Chem. Phys.* **2019**, *151*, 121101.

[7] R. Dovesi, A. Erba, R. Orlando, C. M. Zicovich-Wilson, B. Civalleri, L. Maschio, M. Rerat, S. Casassa, J. Baima, S. Salustro, B. Kirtman, *WIREs Comput. Mol. Sci.* **2018**, *8*, e1360.

[8] I. J. Bush, S. Tomic, B. G. Searle, G. Mallia, C. L. Bailey, B. Montanari, L. Bernasconi, J. M. Carr, N. M. Harrison, *Proc. R. Soc. A: Math. Phys. Eng. Sci.* **2011**, *467*, 2112.

[9] H. Kruse, S. Grimme, *J. Chem. Phys.* **2012**, *136*.

[10] J. G. Brandenburg, M. Alessio, B. Civalleri, M. F. Peintinger, T. Bredow, S. Grimme, *J. Phys. Chem. A* **2013**, *117*, 9282.

[11] S. Grimme, J. Antony, S. Ehrlich, H. Krieg, *J. Chem. Phys.* **2010**, *132*, 154104.

[12] S. Grimme, S. Ehrlich, L. Goerigk, *J. Comput. Chem.* **2011**, *32*, 1456.
